# Jatrophane Diterpenoids from *Euphorbia peplus* Linn. as Activators of Autophagy and Inhibitors of Tau Pathology

**DOI:** 10.3390/ijms24021088

**Published:** 2023-01-06

**Authors:** Ying Yan, Qi Zhou, Xiaoqian Ran, Qingyun Lu, Cuishan Zhang, Mingyou Peng, Lei Tang, Rongcan Luo, Yingtong Di, Xiaojiang Hao

**Affiliations:** 1 State Key Laboratory of Functions and Applications of Medicinal Plants & College of Pharmacy, Guizhou Provincial Engineering Technology Research Center for Chemical Drug R&D, Guizhou Medical University, Guiyang 550014, China; 2 State Key Laboratory of Phytochemistry and Plant Resources in West China, Kunming Institute of Botany, Chinese Academy of Sciences, Kunming 650201, China; 3 Kunming College of Life Science, University of Chinese Academy of Sciences, Kunming 650204, China; 4 Key Laboratory of Animal Models and Human Disease Mechanisms of the Chinese Academy of Sciences & Yunnan Province, and KIZ-CUHK Joint Laboratory of Bioresources and Molecular Research in Common Diseases, Kunming Institute of Zoology, Chinese Academy of Sciences, Kunming 650204, China

**Keywords:** euphjatrophane, *Euphorbia peplus*, jatrophane diterpenoid, autophagy, Tau pathology

## Abstract

Ten jatrophane diterpenoids were isolated from the whole plant *Euphorbia peplus* Linn. including seven new ones, named euphjatrophanes A-G (labeled compounds **1**, **2**, **4**–**8**). Their structures were elucidated with a combination of spectroscopic and single-crystal X-ray crystallography, enabling the identification of compounds **3**, **9**, and **10** as the previously published euphpepluones G, K, and L, respectively. All compounds were evaluated for their bioactivity with flow cytometry in assays of autophagic flux in HM Cherry-GFP-LC3 (human microglia cells stably expressing the tandem monomeric mCherry-GFP-tagged LC3) cells. Euphpepluone K (**9**) significantly activated autophagic flux, an effect that was verified with confocal analysis. Moreover, cellular assays showed that euphpepluone K (**9**) induced autophagy and inhibited Tau pathology.

## 1. Introduction

Alzheimer disease (AD), the most common type of dementia, affects 4–8% of elderly individuals and is characterized by the accumulation of extracellular senile plaques and neuron fibrillary tangles (NFTs) primarily composed of amyloid-β (Aβ) and hyperphosphorylated microtubule assisted protein Tau (MAPT), respectively [1]. Therefore, targeting A*β* and Tau clearance is a promising strategy for treating AD [2,3,4,5,6]. The autophagy and the ubiquitin-proteasome system pathways are the two major degradation systems that degrade abnormal proteins [7,8]. The three types of autophagy (including macro-autophagy, chaperone-mediated autophagy, and micro-autophagy) are mechanistically different from one another. Macroautophagic (henceforth referred to as autophagy) is important for the degradation of misfolded proteins in the maintenance of homeostasis, especially in long-lived cells such as neurons. Autophagy deficits have been implicated in the pathogenesis of AD [9,10,11,12,13,14], and induction of autophagy is a promising therapeutic strategy for AD its treatment [15,16].

The genus from *Euphorbia* (family Euphorbiaceae) is the third largest genus in flowering plants, comprising almost 2000 species [17]. In recent years, various classes of macrocyclic diterpenoids have been isolated and identified from this genus, including jatrophane, pepluane, and ingenane diterpenoids, et al. [18]. Recently, our group had found that a derivative of ingenane, 20-deoxygenol 5-angelate, could promote lysosomal biogenesis. In vivo studies had indicated that this compound could clear amyloid-β in the brain of mice, suggesting that it has the potential to be used in the treatment of neurodegenerative diseases [19]. Besides ingenane diterpenoids, jatrophane diterpenoids featuring a 5/12 bicyclic pentadecane skeleton are the other major chemical constituents from the genus of Euphorbia [20]. Further modifications in their bicyclic skeleton with different oxygenated substituents and stereochemical features have created structural diversity [21]. Moreover, some jatrophane diterpenoids had recently been reported to promote the biogenesis of lysosome, followed by an activation of autophagy [22,23]. Whether more efficient autophagy inducers from structurally diversified jatrophane diterpenoids could be found is an interesting project.

In our continual search for natural products as activator of autophagy, *Euphorbia peplus* Linn. attracted our attention. The *E. peplus* is a small annual weed indigenous to Europe and North Africa. Currently, it is distributed widely throughout the world and has been used in Chinese folk medicine to treat skin lesions, cancers, inflammatory diseases, asthma, and diabetes [24]. Phytochemical investigations on this species indicated the presence of a series of diterpenoids with jatrophane, ingenane, paraliane, segetane, and pepluaneskeletons [22,23,24,25,26,27]. Among them, jatrophane diterpenoids showed significant structural diversity, in which its C-3, C-5, C-7, C-9, C-11, C-14, and C-15 are often replaced by oxygen-containing groups. However, we still do not know which jatrophane diterpenoids within *E. peplus* exerts could activate autophagy. Herein, we reported the isolation, structural elucidation, and potential activity of activating autophagy of jatrophane diterpenoids from the title plant.

## 2. Results

**Figure 1:**
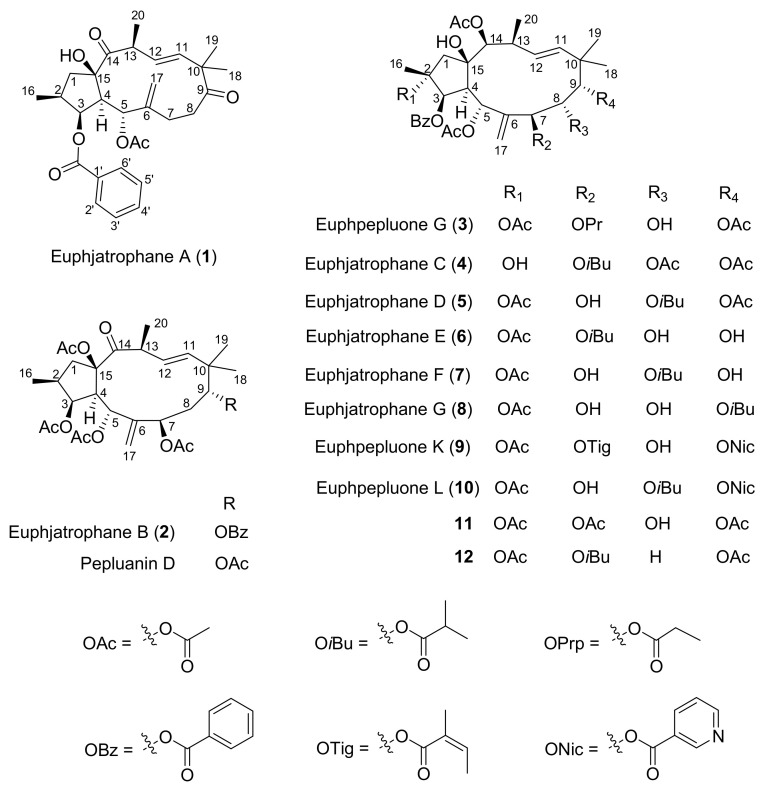
Structures of the jatrophane diterpenoids.

Ten jatrophane diterpenoids, including seven new ones, named euphjatrophanes A (**1**), B (**2**), C (**4**), D (**5**), E (**6**), F (**7**), and G (**8**). were isolated from *E. peplus* (Figure 1). Their structures were determined using a combination of spectroscopic methods and were further confirmed with single-crystal X-ray diffraction. Compounds **3**, **9**, and **10** were identified as the previously published euphpepluones G (**3**), K (**9**), and L (**10**), respectively. Compound **9** significantly activated autophagic flux. Moreover, cellular assays showed that euphpepluone K (**9**) induced autophagy and inhibited Tau pathology.

### 2.1. Structure Elucidation

Euphjatrophane A (**1**) was obtained as colorless crystals (MeOH), and exhibited a molecular formula of C_29_H_36_O_7_, according to the molecular ion peak at *m/z* 519.2346 [M + Na]^+^ (calculated for C_29_H_36_O_7_Na, 519.2353) determined with high-resolution electrospray ionization mass spectrometry (HRESIMS), corresponding to 12 indices of hydrogen deficiency. Its ^1^H and ^13^C-DEPT NMR spectra indicated the presence an acetyl group [*δ*_H_ 1.84 (s, H-2″); *δ*_C_ 169.2 and 20.7], a benzoyl group [*δ*_H_ 8.11 (d, *J* = 7.9 Hz, H-2′, H-6′), 7.56 (t, *J* = 7.3 Hz, H-4′) and 7.45 (t, *J* = 7.6 Hz, H-3′, H-5′); *δ*_C_ 166.0 (C-7′), 134.0 (C-4′), 129.9 × 2 (C-2′, C-6′), 129.7 (C-1′), and 128.5 × 2 (C-3′, C-5′)], an exocyclic double bond [*δ*_H_ 5.18 (s, H-17a) and 4.82 (s, H-17b); *δ*_C_ 142.4 (C-6) and 114.8 (C-17)], and a *trans*-disubstituted double bond [ *δ*_H_ 5.63 (d, *J* = 16.2 Hz, H-11) and 5.62 (d, *J* = 16.2 Hz, H-12); *δ*_C_ 135.7 (C-11) and 134.1 (C-12)] (Table 1). Further signals in the ^1^H NMR spectrum of euphjatrophane A (**1**) for the resonances of one exchangeable proton [*δ*_H_ 4.28 (s, OH-15)], two oxymethine protons [*δ*_H_ 5.84 (t, *J* = 3.5 Hz, H-3) and 5.74 (d, *J* = 10.2 Hz, H-5)], and four methyl groups [*δ*_H_ 1.05 (d, *J* = 6.4 Hz, H-16), 1.22 (s, H-18), 1.21 (s, H-19), and 1.36 (d, *J* = 6.5 Hz, H-20)] were observed. The ^13^C NMR spectrum displayed signals attributable to two ketocarbonyl groups, four methyls, three methylenes, three methines (two oxygenated), one quaternary carbon, four olefinic carbons, and one oxygenated tertiary carbon, A in addition to the carbon signals assigned to the benzoyl and the acetyl groups. Among the 12 indices of hydrogen deficiency, 10 were attributed to one benzoyl group, one acetyl group, two carbonyls groups, and two double bonds. The remaining two DOUs were assumed to be due to the presence of the bicyclic system of euphjatrophane A (**1**). Therefore, by deduction, it was identified as a jatrophane polyester.

**Figure 2:**
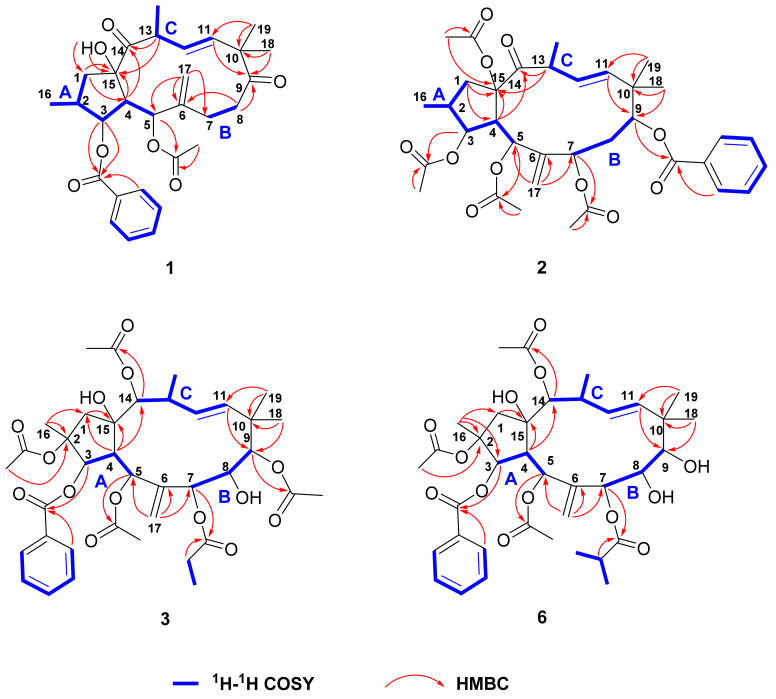
Key HMBC correlations of euphjatrophanes A (**1**), B (**2**), E (**6**), and euphpepluone G (**3**).

Correlation analysis of the ^1^H-^1^H COSY and HSQC spectra of euphjatrophane A (**1**) revealed three fragments in its structural skeleton: C-1/C-2(Me-16)/C-3/C-4/C-5 (fragment A), C-7/C-8 (fragment B), and C-11/C-12/C-13/Me-20 (fragment C) (Figure 2). The connectivity of these fragments was further determined with HMBC correlations (Figure 2). The HMBC correlations from H-1 to C-15, from OH-15 to C-1, and from H-4 to C-15 indicated the presence of a five-membered ring. In addition, the HMBC correlations from H-4 and H-13 to C-14 suggested that fragments A and C are connected through C-14 and C-15. Fragments B and C could be interconnected through *gem*-dimethyl and carbonyl groups, as deduced with the HMBC correlations from H_3_-18 and H_3_-19 to C-9, C-10, and C-11, as well as from H_2_-8 to C-9. The cross peaks of H_2_-17/C-5, C-6, and C-7 in the HMBC spectrum indicated that fragments A and B are connected through the exocyclic double bond C6/C-17. Finally, cross peaks of H-3/the benzoyl carbonyl (*δ*_C_ 166.0), H-5/the acetyl carbonyl (*δ*_C_ 169.2), OH-15/C-15 in HMBC spectrum could located the benzoyloxy, acetoxy, and hydroxyl groups at C-3, C-5, and C-15, respectively. The structure of euphjatrophane A (**1**) is shown in Figure 2. 

**Figure 3:**
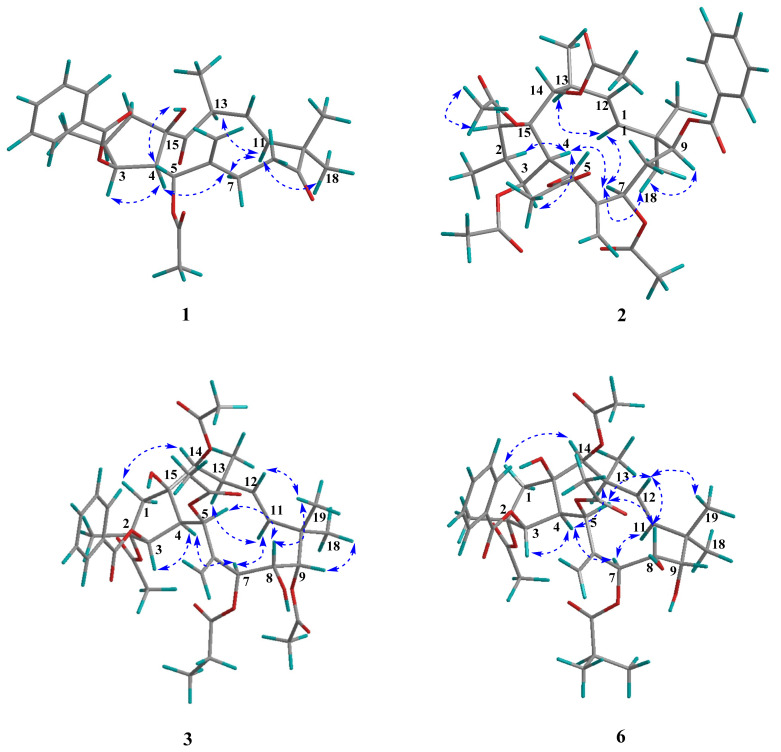
Key ROESY correlations of euphjatrophanes A (**1**), B (**2**), E (**6**), and euphpepluone G (**3**).

**Figure 4:**
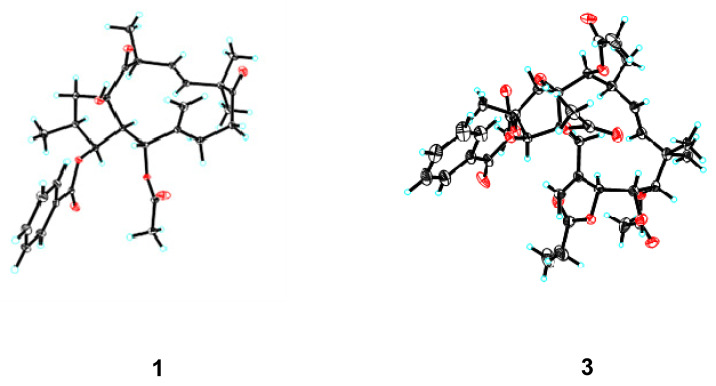
X-ray ORTEP drawings of euphjatrophane A (**1**) and euphpepluone G (**3**).

The stereochemical assignment of euphjatrophane A (**1**) was established with analysis of its coupling constants and ROESY correlations and X-ray crystallography (Figure 3). The large ^3^*J*_4,5_ value (10.2 Hz) of euphjatrophane A (**1**) suggested an endotype conformer with an exomethylene perpendicular to the main plane of the molecule. The geometry of Δ^11^ was assigned as *E* because of the large coupling constant between H-11 and H-12 (*J* = 16.2 Hz). With respect to the flexibility of its 12-membered ring, compound 1′s 3D-structure was ultimately established in the solid state with X-ray diffraction analysis, unambiguously allowing the identification of its absolute configuration as 2S,3S,4R,5R,13S,15R with Cu K*α* radiation [CCDC: 2224640, Flack parameter: −0.05(3)] (Figure 4). We then analyzed the stereo-structure of 1 in solution using its ROESY data. The ROESY interactions of H-3/H-4, H-4/H-7, H-4/H-13, H-11/H-13, H-11/Me-18, and H-13/H-1*α* suggested that these protons and the Me-18 were co-facial and *α*-oriented. Moreover, the ROESY correlations of H-1*β*/OH-15 and H-12/Me-20 indicated their β-orientation. The stereo-structure of euphjatrophane A (**1**) in solution constructed with ROESY correlation matched well with that observed in the solid state. The 1D, 2D, UV, IR and HRESIMS spectrums of compound **1** are shown in Figures S1–S10. 

Euphjatrophane B (**2**) was isolated as a white powder and was assigned a molecular formula of C_35_H_44_O_11_, as deduced from its (+)-HRESIMS (*m/z* 663.2787 [M + Na]^+^, calculated for C_35_H_44_O_11_Na, 663.2776). The ^1^H and ^13^C NMR NMR data (Table 1) of **2** showed a high similarity to pepluanin D [28], except that one benzoyl group (*δ*_H_ 7.47, 7.56, 8.07; *δ*_C_ 165.4, 133.2, 129.8, 129.6, 128.7) occurring in euphjatrophane B (**2**) instead of one acetyl in the latter (Table 1). A combined analysis of the HSQC, HMBC, and ^1^H-^1^H COSY spectra allowed us to identify euphjatrophane B (**2**) as shown and to locate the four acetyloxy groups at C-3, C-5, C-7, and C-15, and one benzoyloxy group at C-9, respectively. In addition, the relative configuration of euphjatrophane B (**2**) was established as shown on the basis of the ^13^C NMR shifts and NOE data (Figure 3). The 1D, 2D, UV, IR and HRESIMS spectrums of compound **2** are shown in Figures S11–S20. 

**Figure 5:**
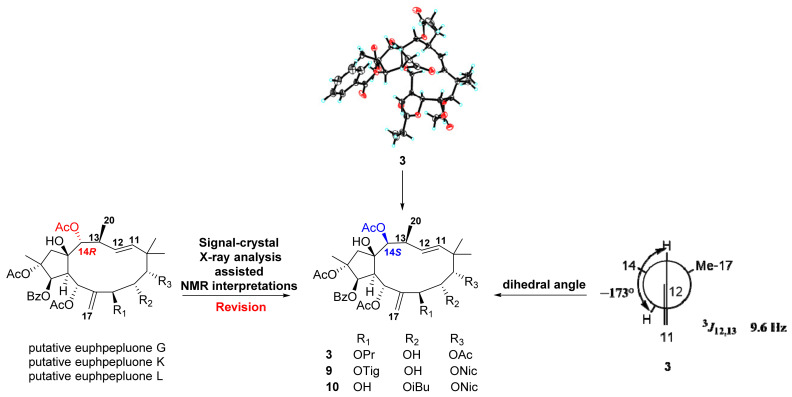
Newman projections along the C-12/C-13 bond of euphpepluone G (**3**), putative euphpepluones G, K, and L and corresponding revised structures of compounds **3**, **9**, and **10**.

Euphpepluone G (**3**) was obtained as a white powder. The identification of its molecular formula as C_38_H_50_O_14_ was based on its HRESIMS ion at *m/z* 753.3094 [M + Na]^+^ (calculated for 753.3093). The ^1^H and ^13^C NMR data of compound 3 were close to those of the isolated known compound 2,5,7,9,14-pentaacetoxy-3-benzoyloxy-8,15-dihydroxyjatropha-6(17), 11*E*-diene (**11**) [29], except for the absence of the resonances from one acetoxy group and the presence of one propionyloxy group (*δ*_H_ 1.17, 2.36; *δ*_C_ 171.9, 27.5, 9.0) (Table 2). The HMBC correlations from H-7 (*δ*_H_ 5.24) to a carbonyl carbon (*δ*_C_ 172.0) revealed that the propionyloxy group was located at C-7. The relative configuration of compound **3** was determined to be the same as that of euphpepluone G (**3**) based on their similar ^1^H, ^13^C NMR data, and ROESY correlations. A subsequent single-crystal X-ray diffraction study by Cu K*α* radiation [CCDC: 2224638, Flack parameter: −0.10(18)] allowed the full assignment of the absolute configuration of compound **3** as 2R,3R,4S,5R,7S,8S,9S,13S,14S,15R (Figure 4). Furthermore, comparison of the NMR data of euphpepluone G (**3**) (recorded in CDCl_3_) with those of euphpepluone G indicated that they matched very well, suggesting that euphpepluone G could be misassigned [30]. In the original paper, *β*-orientation of H-14 was determined with the ROESY correlation to be between H-14 and its neighboring Me-20. However, in the crystal structure of compound **3**, H-14 is in a quasi-equational position, dictating outside its 12-membered ring; thus, the ROESY correlation of H-14/Me-20 is not suitable to determine the relative configuration of H-14. Considering the coupling constant of 9.6 Hz between H-12 and H-13 in euphpepluone G (**3**), the dihedral angle between H-12 and H-13 was approximately 173° in Merck molecular force field (MMFF) modeling (Figure 5). Therefore, H-12 and H-13 are in antiperiplanar orientation. Its ROESY correlations with H-5 and H-14 indicated that H-14 was α-oriented, a finding that is also in excellent agreement with the crystal result of compound **3**. Thus, we are confident that the structure of euphpepluone G should be revised as that of compound **3**. The 1D, 2D, UV, IR and HRESIMS spectrums of compound **3** are shown in Figures S21–S30. 

Euphjatrophane C (**4**) was also obtained as an amorphous white solid and had given the molecular formula C_43_H_66_O_13_, based on its HRESIMS ion at *m/z* 767.3265 [M + Na]^+^ (calculated for C_43_H_66_O_13_Na, 767.3249), This formula is the same as the known compound 2,5,9,14-Tetraacetoxy-3- benzoyloxy-8,15-dihydroxy-7-isobutyroyloxyjatropha-6(17),11E-diene (**12**) [20]. The ^1^H and ^13^C NMR data (Table 2) of euphjatrophane C (**4**) were similar to those of the latter, except that one acetoxy group substitution (*δ*_H_ 2.04; *δ*_C_ 169.8, 20.8) at C-2 in the latter was switched to C-8 in euphjatrophane C (**4**). This finding was further supported by the HMBC cross-peak from H-8 (*δ*_H_ 4.97) to the carbonyl carbon of the acetoxy group (*δ*_C_ 169.8). In addition, the relative configuration at the remaining chiral centers of euphjatrophane C (**4**) would be analogous to those of the latter on the basis of ^13^C NMR shifts and NOE data. The 1D, 2D, UV, IR and HRESIMS spectrums of compound **4** are shown in Figures S31–S40. 

Euphjatrophane D (**5**) was also obtained as an amorphous white solid and had given the molecular formula C_39_H_52_O_14_, based on its HRESIMS ion at *m/z* 767.3246 [M + Na]^+^ (calculated for C_39_H_52_O_14_Na, 767.3249). The ^1^H and ^13^C NMR data of euphjatrophane D (**5**) were closely related to those of the isolated euphpepluone G (**3**), except for one isobutyryloxy group (*δ*_H_ 2.50, 1.05, 1.12; *δ*_C_ 174.8, 34.0, 18.8, 19.2) occurring in euphjatrophane D (**5**) instead of the hydroxyl group in euphpepluone G (**3**) (Table 3). We used 2D NMR analysis, including the HSQC, HMBC, and ^1^H-^1^H COSY spectra, to locate the four acetyloxy groups at C-2, C-5, C-7, and C-14, one benzoyloxy group at C-3, and one isobutyryloxy group at C-8, respectively. Furthermore, the relative configurations at the chiral carbons in euphjatrophane D (**5**) was elucidated to be same as those of euphpepluone G (**3**) according to the ROESY data of euphjatrophane D (**5**) and the similarity of the ^13^C NMR chemical shifts of euphjatrophane D (**5**) to those of euphpepluone G (**3**). The 1D, 2D, UV, IR and HRESIMS spectrums of compound **5** are shown in Figures S41–S50. 

Euphjatrophanes E,-G (**6**–**8**) possessed the same molecular formula as C_37_H_50_O_13_. Analysis of their NMR data indicated that euphjatrophanes E-G (**6**–**8**) possess the same jatrophane skeleton, but with different substituent positions of only one isobutyryloxy group (Table 3 and Table 4). HMBC correlations between H-7 (*δ*_H_ 5.40) and the carbonyl group of the isobutyryloxy group (*δ*_C_ 174.8) in euphjatrophane E (**6**), between H-8 (*δ*_H_ 5.09) and the carbonyl group of the isobutyryloxy group (*δ*_C_ 175.3) in euphjatrophane F (**7**), and between H-9 (*δ*_H_ 4.70) and the carbonyl (*δ*_C_ 178.2) of the isobutyryloxy group in euphjatrophane G (**8**) indicated that the isobutyryloxy group was located at C-7 in **6**, C-8 in **7**, and C-9 in **8**, respectively. The remaining relative configurations of euphjatrophanes E-G (**6**–**8**) were the same as those of euphpepluone G (**3**) on the basis of very similar ^13^C NMR shifts and NOE data. The 1D, 2D, UV, IR and HRESIMS spectrums of compound **6**–**8** are shown in Figures S51–S80. 

Compounds **9** and **10** were identified as euphpepluones K and L (Table S1) [30], respectively, given that their identical NMR and MS data were identical to those of the latter published in the literature, OAc-14 was assigned *α*-orientation (Table 4). Because the relative configuration of OAc-14 was revised from α to β in 3, we scrupulously reexamined the 2D NMR data of compounds **9** and **10**. Specifically, the ROESY correlation of H-13 with H-14 and H-5 in compounds **9** and **10** indicated that H-14 was α-oriented. Thus, the structures of euphpepluones K and L (**9** and **10**) should be revised as shown (Figure 5). The 1D, 2D, UV, IR and HRESIMS spectrums of compound **9** and **10** are shown in Figures S91–S100. 

### 2.2. Bioactivity of the Compounds towards Autophagic Flux

**Figure 6:**
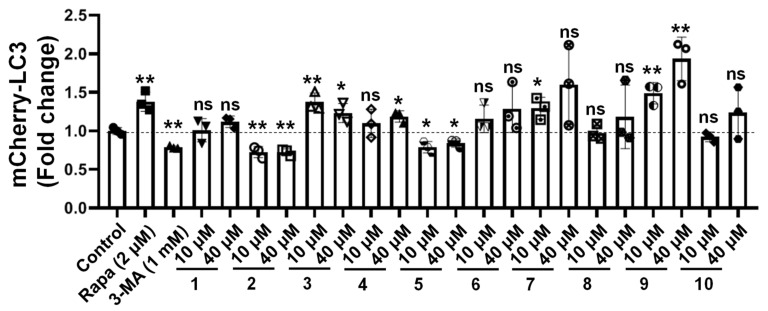
The bioactivity of the compounds towards autophagic flux by flow cytometry analysis. Euphjatrophane A (**1**), euphjatrophane B (**2**), euphpepluone G (**3**), euphjatrophane C (**4**), euphjatrophane D (**5**), euphjatrophane E (**6**), euphjatrophane F (**7**), euphjatrophane G (**8**), euphpepluone K (**9**), and euphpepluone L (**10**). 3-Methyladenine (3-MA; 500 μM) and rapamycin (Rapa, 2 μM) were used as the positive controls. The results are presented as the mean ± SD (n = 3): *, *p* < 0.05; **, *p* < 0.01; ns, not significant; compared to control. Bars represent mean ± SD.

**Figure 7:**
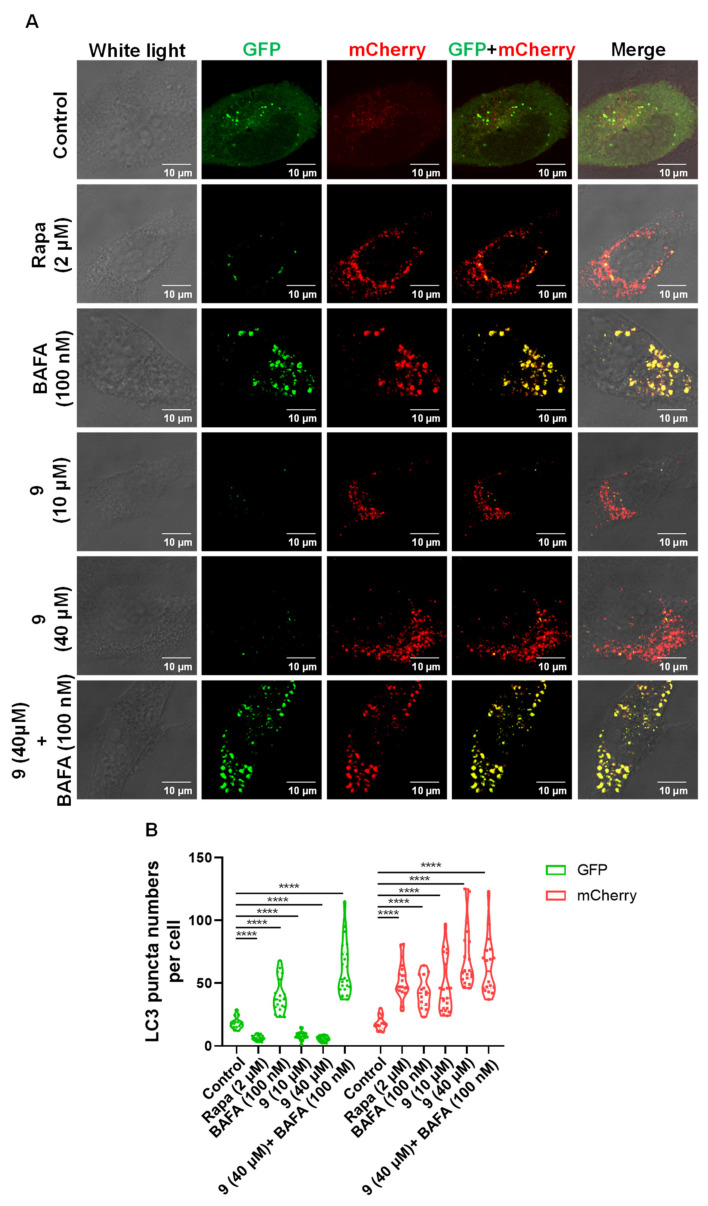
Euphpepluone K (**9**) increases autophagic flux. (**A**) Representative images of HM mCherry-GFP-LC3 cells with treatment of different concentrations of euphpepluone K (**9**), rapamycin (Rapa, 2 μM) or/and bafilomycin A1(BAFA1, 100 nM) Scale bars, 10 μm. (**B**) Quantification of the LC3 puncta in C, GFP for the autophagosome, and mCherry for the autolysosome. Data shown are representative of three experiments. ****, *p* < 0.0001; one-way ANOVA with the Dunnett’s post hoc test. Bars represent mean ± SD.

To test whether these compounds could enhance the autophagy-lysosomal system, we created a human microglia cell line that stably expressed a triple fusion protein (red fluorescent protein (mCherry), green fluorescent protein (GFP), and the autophagosome marker LC3) in a human microglia cell (HM-mCherry-GFP-LC3) that can directly reflect the strength of autophagic flux. We used Rapamycin (Rapa, an inducer of autophagy) and 3-Methyladenine (3-MA, an inhibitor of autophagy) as positive controls. Unexpectedly, we found that euphpepluones G and K (**3** and **9**) significantly increased autophagic flux, whereas euphjatrophanes B and D (**2** and **5**) significantly decreased, and euphjatrophanes A, C, E, F, G and euphpepluone G (**1**, **4**, **6**, **7**, **8**, and **10**) did not influence (Figure 6). Because euphpepluone K (**9**) had the most promising activity in the induction of autophagic flux, we conducted in-depth research using confocal analysis. Again, we found that euphpepluone K (**9**) significantly increased autophagic flux. The increased flux was characterized by an increased number of red puncta and a decreased number of green puncta in the euphpepluone K (**9**)-treated HM mCherry-GFP-LC3 cells (Figure 7), as determined with a laser scanning confocal microscope. This effect could be abolished when the cells were pretreated with bafilomycin A1 (BAFA1) (Figure 7), an inhibitor of the vacuolar (V)-type H+ -translocating ATPase that results in blockage of autophagosome-lysosome fusion [31]. These results suggest that euphpepluone K (**9**) is a positive regulator of autophagy. 

**Figure 8:**
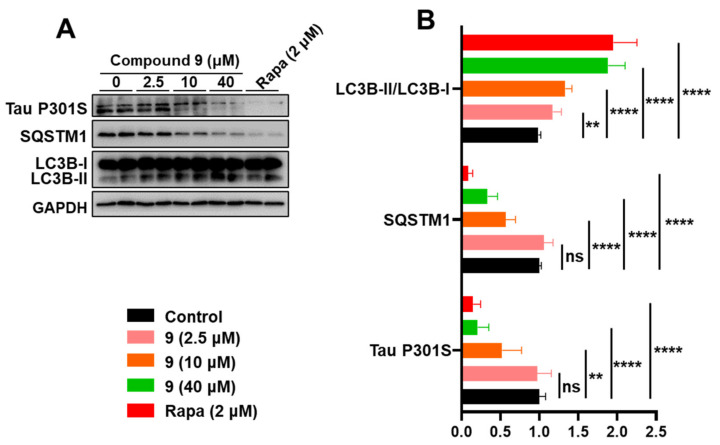
Euphpepluone K (**9**) activates autophagy and inhibits Tau pathology. (**A**,**B**) Western blotting analysis of autophagy markers LC3B-II/LC3B-I and SQSTM1, and Tau P301S in cell lysates from U251-MAPT cells treated with euphpepluone K (**9**) (0, 2.5, 10 and 40 μM), or rapamycin (Rapa, 2μM). A representative western blotting result (**A**) and quantification of respective protein levels (**B**) based on three independent experiments. Relative protein abundance was normalized to GAPDH. ns, not significant; **, *p* < 0.01, ****, *p* < 0.0001; one-way ANOVA with the Dunnett’s post-hoc test. Bars represent mean ± SD.

To test whether euphpepluone K (**9**) has potential biological activity against Tau pathology, cellular analysis was conducted by using a cellular AD model created by the authors: human glioma U251 cells stably expressing the human MAPT mutant (MAPT p.P301S) (U251-MAPT P301S cells). We used Rapa as a positive control in this assay. We found that euphpepluone K (**9**) activates autophagy and inhibits Tau pathology, as indicated by the increased LC3B-II/LC3B-I and decreased SQSTM1 and Tau P301S (Figure 8). 

## 3. Discussion

Jatrophane diterpenoids occur exclusively in the Euphorbiaceae family. Its C-3, C-5, C-7, C-9, C-11, C-14, and C-15 are often replaced with oxygen-containing groups to form jatrophane polyesters, which exhibit rich structural diversity resulting from the difference in the numbers, quantities and locations of the substituents. Structurally, the 12-membered ring is generally highly flexible and can adopt different conformations depending on the substitution pattern. The perpendicular conformation I [in euphjatrophane A (**1**)] and parallel conformation II [in euphjatrophanes B-G (**2**, **4**–**8**), euphpepluones G, K and L (**3**, **9**, and **10**)] were subclassified according to the orientation of the 6,17-exomethylene group. However, it is noteworthy that all signals in the spectra of 1–10 appeared sharp; moreover, the crystal structures of euphjatrophane A (**1**) and euphpepluone G (**3**) were in excellent agreement with the experimental results. This reflected the determination that the NOE effect between H-14 and H-13 could determine the relative configuration of H-14.

Autophagy is a conserved intracellular degradation pathway that delivers unwanted or damaged organelles and misfolded proteins to lysosomes to be broken down. Autophagy is essential for cell survival and metabolism, and protein and organelle quality control [30,31,32]. Because autophagy defects play an important role in the pathogenesis of AD, upregulation of autophagy has been suggested to be beneficial in preventing this disease [33,34,35]. Pharmacological strategies to activate autophagy based on screens for autophagy inducers from small-molecule libraries or FDA-approved drug pools have been studied recently [36,37,38]. Many natural compounds have pharmacological and clinical properties and they compose a natural reservoir of small and large molecules for drug discovery. 

Euphorbia diterpenoids (such as phorbol esters, Mezerein, and Ingenol) are well-known PKC modulators [39]. All simulate the effects of diacylglycerol (DAG) through interacting with the C1 domain of protein kinase C (PKC), an effect that is followed by the regulation of vital cellular processes, including proliferation, survival, motility, and apoptosis via the phosphoinositide signaling pathway, which is also supported by their docking results. Recently, our work demonstrated that PKC modulators could activate autophagy via the promotion of the biogenesis of lysosome. Since jatrophane diterpenoids also belong to Euphorbia diterpenoids, we hypothesized that these jatrophane diterpenoids might also interact with the C1B domain of PKC to activate autophagy. Therefore, we docked jatrophane diterpenoids 4–10 with the C1B domain from PKCδ. As a result, the most efficient autophagy inducer, euphpepluone K (**9**), could not enter the same regulation region of the C1B as PKC modulators phorbol easters, mezerein, and ingenol. Its mechanism to induce autophagy remains to be elucidated.

The current study had several limitations. First, although the jatrophane diterpenoids were dramatically structurally diverse in E. peplus, the amount of each was very low, hampering further evaluation of their functions in animal models. Second, we tested only the effect of euphpepluone K (**9**) on autophagy and Tau pathology. We observed significant autophagy activation and Tau pathology inhibition, but the effect on Tau pathology directly regulated by autophagy remains to be further explained. A focused study to test the mechanism by which euphpepluone K (**9**) activates autophagy and inhibits Tau pathology in vivo and in vitro should be completed in the future.

In conclusion, we have reported on ten jatrophane diterpenoids from E. peplus, including seven new ones. The configurations at C-14 of euphpepluones G, K, and L were revised from 14R to 14S. We determine that euphpepluone K (**9**) induced autophagy and inhibited Tau pathology based on the following evidence: (1) euphpepluone K (**9**) promoted autophagy in HM mCherry-GFP-LC3 cells, as determined with flow cytometry and confocal analysis, and (2) autophagy was increased and Tau P301S was decreased in U251-MAPT P301S cells after treatment with euphpepluone K (**9**). Further well-designed in vitro and in vivo studies in animal models of AD are needed to determine whether euphpepluone K (**9**) could be used to treat autophagy-related AD [30,35,36]. 

## 4. Materials and Methods

### 4.1. General Experimental

Melting points were measured using a Yuhua X-4 digital microdisplay melting point apparatus. X-ray data were collected using a Bruker APEX DUO instrument. Optical rotation was determined on a Horiba SEPA-300 polarimeter (Horiba, Tokyo, Japan). UV spectroscopic data were measured on a Shimadzu-210A double-beam spectrophotometer. IR spectra of samples in KBr discs were recorded on a Bruker-Tensor-27 spectrometer with KBr pellets (Bruker, Germany). ECD spectra were recorded with an Applied Photophysics Chirascan spectrometer (Applied Photophysics, UK). 1D and 2D NMR spectra were recorded on Bruker AM-400, Bruker DRX-500, and Bruker Avance III 600 spectrometer (Karlsruhe, Germany) with TMS as an internal standard. HREIMS was performed with an API QSTAR time-of-flight spectrometer. HPLC preparation was performed on an Agilent 1200 series instrument equipped with a quaternary pump, vacuum degasser, autosampler, thermostatted column compartment, and a diode array detector plus a Waters X-Bridge C_18_ (4.6 × 250 mm) column. The detection wavelengths were set to 210 nm and 254 nm. Fractions were monitored with thin-layer chromatography (TLC, HSGF254, Yantai Jiangyou silica Gel Development Co., Ltd. Yantai, China) and were visualized by spraying with Vanillin chromogenic agent. Silica gel (90–150 μm, Qingdao Marine Chemical Ltd. Qingdao, China), Sephadex LH-20 gel (40–70 μm, GE Healthcare, Sweden). and Lichroprep RP-C18 gel (40–63μm, Merck, Darmstadt, Germany). TLC spots were visualized under UV light after dipping into 5% H_2_SO_4_ in EtOH followed by heating.

### 4.2. Plant Material 

The whole plant parts of *E. peplus* were collected in July 2020 from Kunming Botanical Garden, Yunnan Province, People’s Republic of China (location: 102° 44′ E, 25° 07′ N, at an altitude of 1800–2000 m). The plant was identified by Prof. Hu Shi-Jun (Kunming Institute of Botany, Chinese Academy of Sciences). A voucher specimen (no. kep-09-13) has been deposited in the herbarium of the Kunming Institute of Botany, Chinese Academy of Science.

### 4.3. Extraction and Isolation 

The air-dried whole plants of *E. peplus* (150 kg) were powdered and extracted with methanol (total 900 L) at room temperature thrice. The crude extract (3.0 Kg) acquired under reduced pressure, was applied to a silica gel column using petroleum (PE)/ethyl acetate (EtOAc) (100:1–0:1, *v/v*) to obtain four fractions (F1–F4). Fraction F2 (800 g) was separated using middle chromatogram isolated gel (MCI gel) (CH_3_OH–H_2_O, 4:6–9:1) to obtain fifteen fractions (F2-1–F2-15). The F2-5 (50 g) was applied to another silica gel CC (550 g, 200–300 mesh using a gradient of PE–EtOAc 20:1–1:1) to afford F2-5H (700 mg), which was further treated with Sephadex LH-20 (MeOH/CH_2_C_l2_ 50:50) to obtain seven subfractions of F2-5H1–F2-5H7. Fraction F2-5H5 (40 mg) was purified with silica gel column chromatography, using PE–EtOAc (20:1 to 1:1, *v/v*) to obtain F2-5H5a (28 mg). The F2-5H5a was purified by semi-preparative HPLC (60% CH_3_CN in water) to afford euphjatrophane A (**1**) (4.0 mg) and euphjatrophane D (**5**) (10.0 mg). F2-5P (330 mg) was purified with Sephadex LH-20 and eluted with MeOH to yield five fractions of F2-5P1–F2-5-P5. The F2-5P5 (43 mg) was purified with semi-preparative HPLC (55% CH_3_CN in water) to afford euphjatrophane E (**6**) (10.0 mg) and euphjatrophane F (**7**) (7.8 mg). Fraction F2-5P3 (178 mg) was purified with silica gel column chromatography, using petroleum dichloromethane/ethyl acetate (20:1 to 1:1, *v/v*) to obtain F2-5P3a–F2-5P3e, F2-5P3b was purified with semi-preparative HPLC (57% CH_3_CN in water) to afford euphjatrophane B (**2**) (12.0 mg) and euphjatrophane C (**4**) (8.2 mg). The F2-5P3d was purified with semi-preparative HPLC (63% CH_3_CN in water) to obtain euphpepluone G (**3**) (10.0 mg), euphjatrophane G (**8**) (8.0 mg), euphpepluone K (**9**) (4.7 mg), and euphpepluone L (**10**) (4.0 mg).

#### 4.3.1. Euphjatrophane A (**1**) 

Colorless prismatic crystals (MeOH); mp 148–149 °C; [α${]}_{D}^{24}$ + 191.7 (*c* 0.09, MeOH); UV (MeOH) λ_max_ (log ε) 195 (4.82), 228 (4.07); CD (MeOH): λ(Δ*ε*) = 204 (−8.8), 217 (−22.7), 237 (−0.7), 297 (14.0); IR (KBr) *ν*_max_ 3464, 2984, 1737, 1715, 1695, 1371, 1280, 1116, 1027, 714 cm^−1^; ^1^H and ^13^C NMR data, see Table 1; HRESIMS *m/z* 519.2346 [M + Na]^+^ (calcd for C_29_H_36_O_7_Na, 519.2353).

#### 4.3.2. Euphjatrophane B (**2**)

White amorphous powder; [α${]}_{D}^{24}$ − 9.0 (*c* 0.14, MeOH); UV (MeOH) λ_max_ (log ε) 195 (4.82), 229 (4.25); CD (MeOH): λ(Δ*ε*) = 201 (35.1), 216 (5.3), 229 (12.2); IR (KBr) *ν*_max_ 3435, 2967, 1737, 1373, 1263, 1237, 1110, 1037, 713 cm^−1^; ^1^H and ^13^C NMR data, see Table 1; HRESIMS *m/z* 663.2787 [M + Na]^+^ (calcd for C_35_H_44_O_11_Na, 663.2776).

#### 4.3.3. Euphpepluone G (**3**)

Colorless cube crystal (MeOH); mp 145–146 °C; [α${]}_{D}^{24}$ + 23.4 (*c* 0.09, MeOH); UV (MeOH) λ_max_ (log ε) 195 (4.66), 230 (4.03); CD (MeOH): λ(Δ*ε*) = 195 (−19.6), 204 (2.1), 226 (−1.7); IR (KBr) *ν*_max_ 3467, 2961, 1728, 1451, 1377, 1246, 1110, 1025, 714 cm^−1^; ^1^H and ^13^C NMR data, see Table 1; HRESIMS *m/z* 753.3094 [M + Na]^+^ (calcd for C_38_H_50_O_14_Na, 753.3093). 

#### 4.3.4. Euphjatrophane C (**4**)

White amorphous powder; [α${]}_{D}^{24}$ + 24.7 (*c* 0.09, MeOH); UV (MeOH) λ_max_ (log ε) 195 (4.99), 230 (4.38); CD (MeOH): λ(Δ*ε*) = 195 (−52.4), 205 (3.1), 220 (−4.0); IR (KBr) *ν*_max_ 3464, 2972, 1748, 1375, 1245, 1029, 713 cm^−1^; ^1^H and ^13^C NMR data, see Table 1; HRESIMS *m/z* 767.3265 [M + Na]^+^ (calcd for C_39_H_52_O_14_Na, 767.3249).

#### 4.3.5. Euphjatrophane D (**5**)

White amorphous powder; [α${]}_{D}^{24}$ − 17.3 (*c* 0.1, MeOH); UV (MeOH) λ_max_ (log ε) 195 (4.87), 230 (4.28); CD (MeOH): λ(Δ*ε*) = 195 (−46.8), 205 (2.1), 225 (4.8); IR (KBr) *ν*_max_ 3435, 2969, 1731, 1376, 1235, 1113, 1026, 713 cm^−1^; ^1^H and ^13^C NMR data, see Table 2; HRESIMS *m/z* 767.3246 [M + Na]^+^ (calcd for C_39_H_52_O_14_Na, 767.3249).

#### 4.3.6. Euphjatrophane E (**6**)

White amorphous powder; [α${]}_{D}^{24}$ − 2.7 (*c* 0.11, MeOH); UV (MeOH) λ_max_ (log ε) 195 (4.70), 230 (4.13); CD (MeOH): λ(Δ*ε*) = 195 (−32.5), 205 (1.2), 225 (−3.7); IR (KBr) *ν*_max_ 3436, 2967, 1731, 1377, 1238, 1114, 1026, 713 cm^−1^; ^1^H and ^13^C NMR data, see Table 2; HRESIMS *m/z* 725.3148 [M + Na]^+^ (calcd for C_37_H_50_O_13_Na, 725.3144).

#### 4.3.7. Euphjatrophane F (**7**)

White amorphous powder; [α${]}_{D}^{24}$ + 0.5 (*c* 0.09, MeOH); UV (MeOH) λ_max_ (log ε) 195 (4.68), 230 (4.04); CD (MeOH): λ(Δ*ε*) = 195 (−30.0), 205 (1.7), 226 (−2.8); IR (KBr) *ν*_max_ 3436, 2965, 1730, 1631, 1377, 1238, 1026, and 713 cm^−1^; ^1^H and ^13^C NMR data, see Table 2; HRESIMS *m/z* 725.3149 [M + Na]^+^ (calcd for C_37_H_50_O_13_Na, 725.3144).

#### 4.3.8. Euphjatrophane G (**8**)

White amorphous powder; [α${]}_{D}^{24}$ + 20.4 (*c* 0.11, MeOH); UV (MeOH) λ_max_ (log ε) 195 (4.87), 230 (4.25); CD (MeOH): λ(Δ*ε*) = 195 (−29.2), 205 (2.7), 228 (−2.7); IR (KBr) *ν*_max_ 3436, 2968, 1727, 1376, 1238, 1116, 1026, 713 cm^−1^; ^1^H and ^13^C NMR data, see Table 3; HRESIMS *m/z* 725.3134 [M + Na]^+^ (calcd for C_37_H_50_O_13_Na, 725.3144).

#### 4.3.9. Euphpepluone K (**9**)

White amorphous powder; [α${]}_{D}^{24}$ + 102.5 (*c* 0.15, MeOH); UV (MeOH) λ_max_ (log ε) 195 (4.96), 224 (4.51); CD (MeOH): λ(Δ*ε*) = 201 (24.4), 219 (−8.9), 234 (15.9); IR (KBr) *ν*_max_ 3436, 2969, 1454, 1727, 1376, 1276, 1234, 1113, 1023, 712 cm^−1^; ^1^H and ^13^C NMR data, see Table 3; HRESIMS *m/z* 820.3546 [M + H]^+^ (calcd for C_44_H_53_NO_14_, 820.3539).

#### 4.3.10. Euphpepluone L (**10**)

White amorphous powder; [α${]}_{D}^{24}$ + 78.1 (*c* 0.11, MeOH); UV (MeOH) λ_max_ (log ε) 195 (4.95), 226 (4.40); CD (MeOH): λ(Δ*ε*) = 195 (−2.6), 203 (10.5), 218 (10.5); IR (KBr) *ν*_max_ 3432, 2970, 1729, 1451, 1377, 1275, 1235, 1114, 1024, 712 cm^−1^; ^1^H and ^13^C NMR data, see Table 3; HRESIMS *m/z* 808.3538 [M + H]^+^ (calcd for C_43_H_53_NO_14_, 808.3539).

### 4.4. Flow Cytometry Analysis

The bioactivity of all the compounds towards autophagic flux were evaluated using HM mCherry-GFP-LC3 cells using a published assay [16]. In brief, the HM mCherry-GFP-LC3 cells were cultured in 12-well plates for 24 h in Dulbecco’s modified Eagle medium (DMEM) supplemented with 10% fetal bovine serum (Gibco-BRL, 10099–141) in a 37 °C incubator with 5% CO_2_ at 95% humidity. The compounds were then applied directly to the culture medium, resulting in final concentrations of (10 μM and 40 μM). Twenty-four hours after treatment, the cells were harvested and fixed using 4% paraformaldehyde (PFA), followed by a flow cytometry test to determine whether autophagic flux was altered. The data were analyzed with FlowJo software (FLOWJO, LLC). 

### 4.5. Tandem mCherry-GFP Fluorescence Microscopy

HM mCherry-GFP-LC3 cells were cultured in glass bottom cell culture dishes (801001, NEST) in DMEM medium supplemented with 10% fetal bovine serum for 24 h in a 37 °C incubator with 5% CO_2_ at 95% humidity. Then, the Rapamycin (Rapa, 2 μM; InvivoGen, trl-rap), Bafilomycin A1 (BAFA1, 100 nM; InvivoGen, trl-baf) and/or compound **9** (10 μM and 40 μM) then added directly to the culture medium. For evaluating tandem fluorescent LC3 puncta, images of the cells were captured with an Olympus FluoView™ 1000 confocal microscope (Olympus, America) 24 h after drug treatment as described in our previous study [29].

### 4.6. Construction of U251 Cells with Stable Expression of the Mutant MAPT (MAPT P301S) Gene

The coding region of the MAPT gene with a Flag tag was cloned into a pLVX vector (pLVX-MAPT) of the Lenti-X Tet-On Advanced Inducible Expression System. Mutated MAPT P301S was introduced into the pLVX vector with the site-directed mutagenesis PCR method by Wuhan Miaoling Biotechnology Co. (Wuhan, China).

The U251 cells were introduced from the Kunming Cell Bank, Kunming Institute of Zoology, Chinese Academy of Sciences. U251 cells were cultured in RPMI 1640 supplemented with 10% fetal bovine serum. A U251 cell line with stable expression of the mutant MAPT P301S (U251-MAPT P301S) was constructed according to the instructions of the Lenti-X Tet-On Advanced Inducible Expression System and following previously reported methods [40]. Briefly, the response lentivirus system was composed of mutant pLVX-MAPT constructs (purchased from Wuhan Miaoling Biotechnology Co.), packaging plasmid psPAX2 (Addgene, UK, 12260), and envelope plasmid PMD2.G (Addgene, UK, 12259), whereas the regulator lentivirus system was composed of pLVX-Tet-On-Advanced vector, psPAX2, and PMD2.G. The lentivirus supernatant produced from HEK293T cells was used to infect U251 cells with a ratio of 4:1 for the response lentivirus and the regulator lentivirus. Infected U251 cells were selected in a growth medium with 1 μg/mL puromycin.

### 4.7. Western Blot

Western blot assays were performed according to the methods in our previous published studies [12,16,41]. In brief, cell lysates of U251-MAPT P301S cells were prepared, and protein concentration was determined with the BCA protein assay kit (Beyotime Institute of Biotechnology, P0012). A total of 20 μg of protein of each sample was separated with 12% sodium dodecyl sulfate-polyacrylamide gel electrophoresis (SDS-PAGE) and transferred to a polyvinylidene difluoride (PVDF) membrane (BioRad, L1620177 Rev D). The PVDF membrane was then soaked in 5% (*w/v*) skim milk at room temperature for 2 h and incubated with primary antibody (SQSTM1, 1:1000, Elabscience, EAP3350; LC3B, 1:1000, Cell Signaling Technology, 3868; Tau, 1:1000, Abcam, ab80579; ACTB, 1:1000, Beijing Zhong Shan-Golden Bridge Biological Technology CO., Ltd., TA-09) overnight at 4 °C. The membrane was washed three times the next day with TBST (Tris-buffered saline [Servicebio, G0001] supplemented with 0.1% Tween 20 [Sigma, P1379]) for 5 min each, wash followed by incubation with the secondary antibody. Secondary peroxidase-labeled affinity-purified goat anti-mouse (474-1806, KPL) and goat anti-rabbit antibodies (474-1516, KPL) were the secondary antibodies. The epitope was visualized using an ECL western blot detection kit (Millipore, WBKLS0500). ImageJ software (National Institutes of Health, Bethesda, MD, USA) was used to evaluate the densitometry of the target protein, as we described our previous study [16]. The western blot for ACTB included a loading control to measure the densitometry of Tau and SQSTM1. The LC3B-II densitometric signal was determined with the ratio of LC3B-II to LC3B-I before normalizing to ACTB.

### 4.8. Statistical Analysis

Data analyses were performed using GraphPad Prism v6.0. One-way analysis of variance (ANOVA) with Dunnett’s *post-hoc* test was used for quantifying difference between the treated group and the control group. Comparisons of the relative protein levels of SQSTM1, Tau P301S and the ratio of LC3B-II:LC3B-I proteins from cells with different treatments were conducted by one-way ANOVA with the Dunnett’s *post-hoc* test. All data are presented as mean ± standard deviation (SD). All tests were two-tailed. A *p*-value of <0.05 was considered statistically significant.

## Figures and Tables

**Table 1:** ^1^H and ^13^C NMR Data of euphjatrophane A (**1**) and euphjatrophane B (**2**) in CDCl_3._.

	Euphjatrophane A (1)		Euphjatrophane B (2)	
Position	*δ*_H_ (*J* in Hz)	*δ*_C_	*δ*_H_ (*J* in Hz)	*δ*_C_
1α	2.41 dd (14.2, 8.4)	46.8	2.99 dd (14.0, 7.6)	46.4
1β	1.93 dd (14.2, 12.2)		1.65 dd (14.0, 12.4)	
2	2.32 m	38.7	2.26 m	38.3
3	5.84 t (3.6)	78.0	5.42 t (3.0)	76.6
4	2.76 dd (10.2, 3.6)	50.5	2.78 br d (3.0)	53.0
5	5.75 d (10.2)	72.9	5.81 br s	68.5
6		142.4		147.4
7α	2.09 m	25.0	4.78 br d (6.8)	69.4
7β	1.87 m			
8α	2.57 m	34.1	2.18 m	33.5
8β	2.23 m		2.05 m	
9		211.6	5.04 m	75.4
10	5.63 d (16.2)	50.4		40.7
11	5.62 d (16.2)	135.7	5.93 d (16.2)	137.9
12	3.45 m	130.9	5.70 dd (16.2, 9.0)	129.9
13		44.2	3.52 m	42.9
14		212.2		212.7
15	4.28 s	84.7		93.0
16	1.05 d (6.4)	14.0	0.88 d (6.6)	13.4
17a	5.18 s	114.8	5.01 m	109.6
17b	4.84 s			
18	1.22 s	23.5	1.13 s	26.2
19	1.21 s	23.7	1.07 s	24.1
20	1.37 d (6.6)	20.9	1.19 d (6.8)	19.5
OAc-2				
C=O				
OAc-3				
C=O				169.8
			2.11 s	21.2
OAc-5				
C=O		169.2		169.6
	1.84 s	20.7	2.13 s	21.3
OAc-7				
C=O				170.2
			1.37 s	20.2
OAc-8				
C=O				
OAc-9				
C=O				
OAc-14				
C=O				
OAc-15				
C=O				170.5
			2.13 s	21.3
OBz-3				
C=O		166.0		
1′		129.7		
2′,6′	8.11 d (7.8)	129.9		
3′,5′	7.45 t (7.6)	128.5		
4′	7.56 t (7.2)	134.0		
OBz-9				
C=O				165.4
1′				129.8
2′,6′			7.99 d (7.8)	129.6
3′,5′			7.46 t (7.8)	128.7
4′			7.56 t (7.4)	133.2
OPrp-7				
C=O				
1″				
2″				
OiBu-7				
C=O				
1″				
2″				
3″				
OH-15	4.31 s			

**Table 2:** ^1^H and ^13^C NMR Data of euphpepluone G (**3**) and euphjatrophane C (**4**) in CDCl_3._.

	Euphpepluone G (3)		Euphjatrophane C (4)	
Position	*δ*_H_ (*J* in Hz)	*δ*_C_	*δ*_H_ (*J* in Hz)	*δ*_C_
1α	2.67 d (15.0)	50.2	2.24 d (15.0)	53.3
1β	2.15 d (15.0)		2.14 d (15.0)	
2		88.4		78.5
3	5.87 d (5.8)	80.4	5.48 d (3.8)	82.4
4	3.36 dd (5.8, 3.6)	44.6	3.37 t (3.6)	44.1
5	5.75 d (3.6)	71.8	5.83 d (3.2)	71.2
6		144		144.1
7α	5.24 s	68.4	5.60 s	67.9
7β				
8α	4.03 d (11.0)	69.9	4.97 s	80.5
8β				
9	4.72 s	86.0	5.23s	70.5
10		40.0		40.7
11	5.92 d (16.0)	133.9	5.83 d (16.0)	133.9
12	5.58 dd (16.0, 9.6)	131.0	5.70 dd (16.0, 9.6)	131.6
13	2.68 m	37.5	2.83 m	36.6
14	5.10 s	79.3	5.13 s	79.7
15		84.0		83.5
16	1.51 s	22.6	1.34 s	24.3
17a	4.87 s	109.4	4.95 s	110.2
17b	4.50 s		4.57 s	
18	1.01 s	27.2	0.92 s	26.3
19	1.28 s	23.2	1.33 s	23.8
20	1.14 d (7.2)	23.6	1.15 d (7.0)	23.4
OAc-2				
C=O		170.7		
	2.15 s	22.3		
OAc-3				
C=O				
OAc-5				
C=O		168.5		168.3
	2.00 s	21.1	1.80 s	20.6
OAc-7				
C=O				
OAc-8				
C=O				169.7
			2.04 s	20.6
OAc-9				
C=O		171.9		169.7,
	2.05 s	20.6	2.02 s	20.6
OAc-14				
C=O		170.4		171.0
	2.08 s	20.5	2.10 s	20.8
OAc-15				
C=O				
OBz-3				
C=O		164.9		165.2
1′		130.0		130.0
2′,6′	8.07 d (8.4)	129.7	8.04 d (8.4)	129.6
3′,5′	7.43 t (7.8)	128.5	7.43 t (7.8)	128.5
4′	7.57 t (7.4)	133.2	7.56 t (7.4)	133.3
OBz-9				
C=O				
1′				
2′,6′				
3′,5′				
4′				
OPrp-7				
C=O		171.9		
1″	2.36 m	27.5		
2″	1.17 t (7.6)	9.0		
OiBu-7				
C=O				176.3
1″			2.66 m	34.1
2″			1.28 d (7.0)	19.3
3″			1.24 d (7.0)	18.5

**Table 3:** ^1^H and ^13^C NMR Data of euphjatrophanes D, E, and F (**5**–**7**) in CDCl_3._.

	Euphjatrophane D (5)		Euphjatrophane E (6)		Euphjatrophane F (7)	
Position	*δ*_H_ (*J* in Hz)	*δ*_C_	*δ*_H_ (*J* in Hz)	*δ*_C_	*δ*_H_ (*J* in Hz)	*δ*_C_
1α	2.70 d (14.2)	49.1	2.61 d (14.6)	50.7	2.67 d (14.6)	49.5
1β	2.07 d (14.2)		2.18 d (14.6)		2.10 d (14.6)	
2		88.7		88.1		88.6
3	5.74 d (5.8)	79.9	5.82 d (5.6)	80.2	5.76 d (5.8)	79.7
4	3.51 m	44.3	3.29 dd (5.6, 3.8)	44.6	3.40 m	44.5
5	5.78, br d (1.8)	72.1	5.73 br d (3.2)	72.0	5.79 br d (3.2)	71.9
6		146.4		144.7		145.3
7	4.33 s	67.9	5.40 s	67.8	4.48 br d (5.0)	68.3
8	5.14 s	71.7	3.96 br d (5.5)	70.7	5.09 s	72.3
9	4.84 s	80.8	3.62 s	84.0	3.61 s	82.2
10		40.4		40.7		41.2
11	5.87 d (16.0)	134.5	5.91 d (16.2)	134.5	5.86 d (16.0)	135.3
12	5.62 dd (16.0, 9.4)	131.2	5.52 dd (16.2, 9.4)	129.9	5.54 dd (16.0, 9.6)	130.2
13	2.61 m	37.7	2.62 m	37.4	2.58 m	37.6
14	5.08 s	79.6	5.08 s	79.6	5.06 s	79.9
15		83.9		83.8		83.7
16	1.50 s	23.3	1.53 s	22.1	1.50 s	22.6
17a	4.93 s	109.2	4.94 s	109.1	5.05 s	109.3
17b	4.53 s		4.52 s		4.54 s	
18	1.01 s	26.9	1.07 s	26.9	1.12 s	26.4
19	1.36 s	23.8	1.19 s	23.8	1.21 s	24.0
20	1.13 d (6.8)	23.3	1.12 d (7.2)	23.6	1.14 d (6.2)	23.5
OAc-2						
C=O		170.6,		170.7		170.9
	2.14 s	22.3	2.14 s	22.3	2.13 s	22.3
OAc-5						
C=O		168.0,		168.7		168.0
	1.93 s	20.9	1.96 s	20.0	1.93 s	20.9
OAc-9						
C=O		170.8,				
	2.11 s	20.5				
OAc-14						
C=O		170.8,		170.4		171.0
	2.17 s	21.0	2.07 s	20.5	2.10 s	20.6
OBz-3						
C=O		165.3		164.8		165.5
1′		129.8		130.0		129.7
2′,6′	8.11 d (8.0)	129.7	8.08 d (8.2)	129.7	8.10 d (8.4)	129.8
3′,5′	7.46 t (7.4)	128.5	7.44 t (7.8)	128.5	7.45 t (7.8)	128.5
4′	7.59 t (7.4)	133.4	7.57 t (7.4)	133.2	7.59 t (7.4)	133.5
OiBu-7						
C=O				174.8		
1″			2.63 m	34.0		
2″			1.20 d (7.0)	18.8		
3″			1.23 d (7.0)	19.0		
OiBu-8						
C=O		174.8				175.3
1″	2.50 m	34.0			2.49 m	34.0
2″	1.05 d (7.0)	18.8			1.07 d (7.0)	18.8
3″	1.12 d (7.0)	19.2			1.03 d (7.0)	19.4

**Table 4:** ^1^H and ^13^C NMR Data of euphjatrophanes G, euphpepluones K and L (**8**–**10**) in CDCl_3._.

	Euphjatrophane G (8)		Euphpepluone K (9)		Euphpepluone L (10)	
Position	*δ*_H_ (*J* in Hz)	*δ*_C_	*δ*_H_ (*J* in Hz)	*δ*_C_	*δ*_H_ (*J* in Hz)	*δ*_C_
1a	2.71 d (14.0)	48.9	2.84 d (14.4)	49.8	2.76 d (14.0)	48.8
1b	2.00 d (14.0)		2.10 d (14.4)		1.98 d (14.0)	
2		88.8		88.5		88.8
3	5.67 d (6.2)	80.2	5.98 (6.0)	80.9	5.70 d (6.6)	80.0
4	3.70 m	44.3	3.70 m	44.9	3.94 m	44.4
5	5.74 d (4.2)	72.5	5.81 br d (3.4)	72.0	5.81 br d (3.8)	72.3
6		147.4		143.8		146.5
7	4.11 d (8.0)	67.0	5.37 s	68.8	4.52 d (8.8)	67.4
8	3.94 d (8.0)	70.5	4.20 d (10.8)	70.3	5.21 s	71.8
9	4.70 s	84.8	5.06 s	86.8	5.07 s	82.2
10		40.1		40.3		40.8
11	5.94 d (16.0)	134.6	6.15 d (16.0)	133.7	6.14 d (16.0)	134.6
12	5.52 dd (16.0, 9.8)	130.5	5.67 dd (16.0, 9.8)	131.6	5.67 dd (16.0, 9.8)	131.4
13	2.62 m	37.9	2.88 m	37.4	2.73 m	38.1
14	5.08 s	69.6	5.15 s	79.4	5.12 s	79.6
15		84.1		84.3		84.2
16	1.47 s	24.0	1.48 s	23.4	1.46 s	24.5
17a	4.91 s	108.2	4.78 s	109.2	4.81 s	108.5
17b	4.70 s		4.49 s		4.41 s	
18	1.04 s	27.2	1.09 s	27.3	1.07s	27.3
19	1.28 s	23.3	1.38 s	23.2	1.44 s	23.6
20	1.12 d (7.2)	23.3	1.18 d (7.2)	23.6	1.14 d (7.0)	23.3
OAc-2						
C=O		170.9,		170.9		171.6
	2.09 s	22.2	2.17 s	22.4	2.23 s	22.5
OAc-5						
C=O		168.6		168.6		167.9
	2.08 s	21.2	2.05 s	21.1	2.07 s	20.5
OAc-14						
C=O		170.4,		170.4		170.6
	2.07 s	20.5	2.11 s	20.5	2.11 s	21.0
OBz-3						
C=O		165.6		165.0		165.5
1′		129.7		130.0		129.7
2′,6′	8.09 d (8.4)	129.8	8.05 d (8.4)	129.7	8.07 d (8.4)	129.7
3′,5′	7.45 t (7.8)	128.5	7.41 t (7.8)	128.4	7.42 t (7.8)	128.4
4′	7.59 t (7.4)	133.4	7.55 t (7.4)	133.2	7.56 t (7.4)	133.4
OTig-8						
C=O				164.2		
1″				126.1		
2″			5.75 m	141.7		
3″			1.68 d (7.2)	15.8		
4″			1.52 m	19.5		
OiBu-9/8		OiBu-9				OiBu-8
C=O		178.2				174.9
1″	2.67 m	34.4			2.53 m	34.0
2″	1.27 d (7.0)	18.7			1.05 d (7.0)	18.8
3″	1.25 d (7.0)	18.8			1.15 d (7.0)	19.3
ONic-9						
C=O				166.0		164.9
2″			9.23 s	151.4	9.39 s	151.3
3″				124.9		126.3
4″			8.29 d (8.0)	137.4	8.41 d (8.0)	137.4
5″			7.34 dd (8.0, 4.8)	123.1	7.39 dd (8.0, 4.8)	123.3
6″			8.76 d (4.8)	153.7	8.78 d (4.8)	153.4

## Data Availability

The data underlying this study are available in the published article and its online Supporting Information.

## Supplementary Materials

The following supporting information can be downloaded at: https://www.mdpi.com/article/10.3390/ijms24021088/s1.
