# Supplementary material for: Jatrophane Diterpenoids from Euphorbia peplus Linn. as Activators of Autophagy and Inhibitors of Tau Pathology
Source: Int J Mol Sci. 2023 Jan 6;24(2):1088. doi: 10.3390/ijms24021088 (PMC9863522; doi:10.3390/ijms24021088)
Supplement: Supplementary file 1 [file ijms-24-01088-s001.zip › ijms-2059894-supplementary.pdf]

Supporting Information

Jatrophane diterpenoids from *Euphorbia peplus* Linn. as activators of autophagy and inhibit Tau pathology

Ying Yan [1,†], Qi Zhou [2,†], Xiaoqian Ran [3,4], Qingyun Lu [2], Cuishan Zhang [2], Mingyou Peng [1], Lei Tang [1], Rongcan Luo [3,4,*], Yingtong Di [2,4,*], Xiaojiang Hao [1,4]

[1] State Key Laboratory of Functions and Applications of Medicinal Plants & College of Pharmacy, Guizhou Provincial Engineering Technology Research Center for Chemical Drug R&D, Guizhou Medical University, Guiyang 550014, China

[2] State Key Laboratory of Phytochemistry and Plant Resources in West China, Kunming Institute of Botany, Chinese Academy of Sciences, Kunming 650201, Yunnan, P. R. China

[3] Key Laboratory of Animal Models and Human Disease Mechanisms of the Chinese Academy of Sciences & Yunnan Province, and KIZ-CUHK Joint Laboratory of Bioresources and Molecular Research in Common Diseases, Kunming Institute of Zoology, Chinese Academy of Sciences, Kunming 650204, Yunnan, China

[4] Kunming College of Life Science, University of Chinese Academy of Sciences, Kunming, Yunnan 650204, China

*Correspondence: luorongcan@mail.kiz.ac.cn (R. Luo), diyt@mail. kib.ac.cn (Y. Di).
† These authors contributed equally to this work.

Contents

S1. X-ray Crystallographic Analysis of Compound **1**. 6

S2. X-ray Crystallographic Analysis of Compound **3**. 6

Figure S1. ^{1}H NMR spectrum of euphjatrophane A (**1**)...7

Figure S2. ^{13}C NMR spectrum of euphjatrophane A (**1**)..8

Figure S3. ^{1}H-^{1}H COSY spectrum of euphjatrophane A (**1**) 8

Figure S4. HSQC spectrum of euphjatrophane A (**1**) 9

Figure S5. HMBC spectrum of euphjatrophane A (**1**) 9

Figure S6. ROESY spectrum of euphjatrophane A (**1**)...10

Figure S7. CD spectrum of euphjatrophane A (**1**)...10

Figure S8. UV spectrum of euphjatrophane A (**1**)...11

Figure S9. IR spectrum of euphjatrophane A (**1**) 11

Figure S10. (+)-HRESIMS spectrum of euphjatrophane A (**1**) 12

Figure S11. ^{1}H NMR spectrum of euphjatrophane B (**2**) 13

Figure S12. ^{13}C NMR spectrum of euphjatrophane B (**2**) 13

Figure S13. ^{1}H-^{1}H COSY spectrum of euphjatrophane B (**2**)...14

Figure S14. HSQC spectrum of euphjatrophane B (**2**) 14

Figure S15. HMBC spectrum of euphjatrophane B (**2**) 15

Figure S16. ROESY spectrum of euphjatrophane B (**2**) 15

Figure S17. CD spectrum of euphjatrophane B (**2**) 16

Figure S18. UV spectrum of euphjatrophane B (**2**)...16

Figure S19. IR spectrum of euphjatrophane B (**2**)...17

Figure S20. (+)-HRESIMS spectrum of euphjatrophane B (**2**)..17

Figure S21. ^{1}H NMR spectrum of euphpepluone G (**3**)..18

Figure S22. ^{13}C NMR spectrum of euphpepluone G (**3**)...18

Figure S23. ^{1}H-^{1}H COSY spectrum of euphpepluone G (**3**) 19

Figure S24. HSQC spectrum of euphpepluone G (**3**) 19

Figure S25. HMBC spectrum of euphpepluone G (**3**) 20

Figure S26.ROESY spectrum of euphpepluone G (**3**)..20

Figure S27. CD spectrum of euphpepluone G (**3**) 21

Figure S28. UV spectrum of euphpepluone G (**3**)........21

Figure S29. IR spectrum of euphpepluone G (**3**)........22

Figure S30. (+)-HRESIMS spectrum of euphpepluone G (**3**)........22

Figure S31. ^{1}H NMR spectrum of euphjatrophane C (**4**)........23

Figure S32. ^{13}C NMR spectrum of euphjatrophane C (**4**)........23

Figure S33. ^{1}H-^{1}H COSY spectrum of euphjatrophane C (**4**)........24

Figure S34. HSQC spectrum of euphjatrophane C (**4**)........24

Figure S35. HMBC spectrum of euphjatrophane C (**4**)........25

Figure S36. ROESY spectrum of euphjatrophane C (**4**)........25

Figure S37. CD spectrum of euphjatrophane C (**4**)........26

Figure S38. UV spectrum of euphjatrophane C (**4**)........26

Figure S39. IR spectrum of euphjatrophane C (**4**)........27

Figure S40. (+)-HRESIMS spectrum of euphjatrophane C (**4**)........27

Figure S41. ^{1}H NMR spectrum of euphjatrophane D (**5**)........28

Figure S42. ^{13}C NMR spectrum of euphjatrophane D (**5**)........28

Figure S43. ^{1}H-^{1}H COSY spectrum of euphjatrophane D (**5**)........29

Figure S44. HSQC spectrum of euphjatrophane D (**5**)........29

Figure S45. HMBC spectrum of euphjatrophane D (**5**)........30

Figure S46. ROESY spectrum of euphjatrophane D (**5**)........30

Figure S47. CD spectrum of euphjatrophane D (**5**)........31

Figure S48. UV spectrum of euphjatrophane D (**5**)........31

Figure S49. IR spectrum of euphjatrophane D (**5**)........32

Figure S50. (+)-HRESIMS spectrum of euphjatrophane D (**5**)........32

Figure S51. ^{1}H NMR spectrum of euphjatrophane E (**6**)........33

Figure S52. ^{13}C NMR spectrum of euphjatrophane E (**6**)........33

Figure S53. ^{1}H-^{1}H COSY spectrum of euphjatrophane E (**6**)........34

Figure S54. HSQC spectrum of euphjatrophane E (**6**)........34

Figure S55. HMBC spectrum of euphjatrophane E (**6**)........35

Figure S56. ROESY spectrum of euphjatrophane E (**6**)........35

Figure S57. CD spectrum of euphjatrophane E (**6**)........36

Figure S58. UV spectrum of euphjatrophane E (**6**)..36
Figure S60. (+)-HRESIMS spectrum of euphjatrophane E (**6**).......................................37
Figure S61. ^{1}H NMR spectrum of euphjatrophane F (**7**)..38
Figure S62. ^{13}C NMR spectrum of euphjatrophane F (**7**)...38
Figure S63. ^{1}H-^{1}H COSY spectrum of euphjatrophane F (**7**)...39
Figure S64. HSQC spectrum of euphjatrophane F (**7**)..39
Figure S65. HMBC spectrum of euphjatrophane F (**7**)..40
Figure S66. ROESY spectrum of euphjatrophane F (**7**)..40
Figure S67. CD spectrum of euphjatrophane F (**7**)..41
Figure S68. UV spectrum of euphjatrophane F (**7**)..41
Figure S69. IR spectrum of euphjatrophane F (**7**)...42
Figure S70. (+)-HRESIMS spectrum of euphjatrophane F (**7**).......................................42
Figure S71. ^{1}H NMR spectrum of euphjatrophane G (**8**)...43
Figure S72. ^{13}C NMR spectrum of euphjatrophane G (**8**)..43
Figure S73. ^{1}H-^{1}H COSY spectrum of euphjatrophane G (**8**)..44
Figure S74. HSQC spectrum of euphjatrophane G (**8**)...44
Figure S75. HMBC spectrum of euphjatrophane G (**8**)...45
Figure S76. ROESY spectrum of euphjatrophane G (**8**)...45
Figure S77. CD spectrum of euphjatrophane G (**8**)...46
Figure S78. UV spectrum of euphjatrophane G (**8**)...46
Figure S79. IR spectrum of euphjatrophane G (**8**)..47
Figure S80. (+)-HRESIMS spectrum of euphjatrophane G (**8**)......................................47
Figure S81. ^{1}H NMR spectrum of euphpepluone K (**9**)..48
Figure S82. ^{13}C NMR spectrum of euphpepluone K (**9**)...48
Figure S83. ^{1}H-^{1}H COSY spectrum of euphpepluone K (**9**)...49
Figure S84. HSQC spectrum of euphpepluone K (**9**)...49
Figure S85. HMBC spectrum of euphpepluone K (**9**)...50
Figure S86. NOESY spectrum of euphpepluone K (**9**)..50
Figure S87. CD spectrum of euphpepluone K (**9**)...51
Figure S88. UV spectrum of euphpepluone K (**9**)...51

Figure S89. IR spectrum of euphpepluone K (**9**) 52
Figure S90. (+)-HRESIMS spectrum of euphpepluone K (**9**) 52
Figure S91. ^{1}H NMR spectrum of euphpepluone L (**10**) 53
Figure S92. ^{13}C NMR spectrum of euphpepluone L (**10**) 53
Figure S93. ^{1}H-^{1}H COSY spectrum of euphpepluone L (**10**) 54
Figure S94. HSQC spectrum of euphpepluone L (**10**) 54
Figure S95. HMBC spectrum of euphpepluone L (**10**) 55
Figure S96. ROESY spectrum of euphpepluone L (**10**) 55
Figure S97. CD spectrum of euphpepluone L (**10**) 56
Figure S98. UV spectrum of euphpepluone L (**10**) 56
Figure S99. IR spectrum of euphpepluone L (**10**) 57
Figure S100. (+)-HRESIMS spectrum of euphpepluone L (**10**) 57
Table S1. ^{1}H and ^{13}C NMR Data of Euphpepluones G, K, and L in $CDCl_3$^{a} 58

S1. X-ray Crystallographic Analysis of Compound **1**.

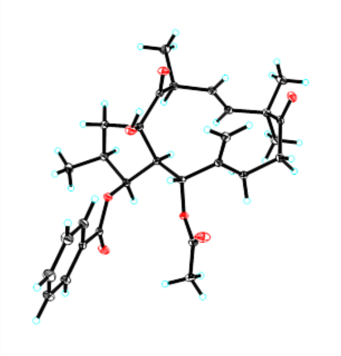

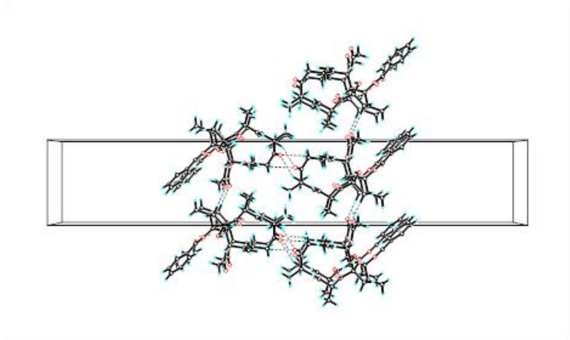

The crystal structure and absolute configuration of **1** were determined by single crystal X-ray diffraction analysis. Crystal data for compound **1**: $C_{29}H_{36}O_7$, M = 496.58, a = 6.36920(10) Å, b = 8.7069(2) Å, c = 48.6471(11) Å, α = 90°, β = 90°, γ = 90°, V = 2697.77(10) Å3, T = 100. (2) K, space group P212121, Z = 4, μ(Cu Kα) = 0.706 mm^{-1}, 43259 reflections measured, 5322 independent reflections (Rint = 0.0351). The final R1 values were 0.0258 (I > 2σ(I)). The final wR(F2) values were 0.0662 (I > 2σ(I)). The final R1 values were 0.0262 (all data). The final wR(F2) values were 0.0666 (all data). The goodness of fit on F2 was 1.029. Flack parameter = 0.05(3). CCDC-2224640 (**1**), contain the supplementary crystallographic data. These data can be obtained free of charge from the Cambridge Crystallographic Data Centre (http://www.ccdc.cam.ac.uk/).

S2. X-ray Crystallographic Analysis of Compound **3**.

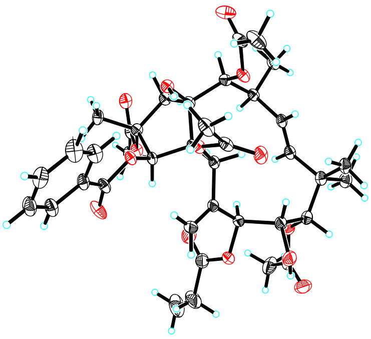

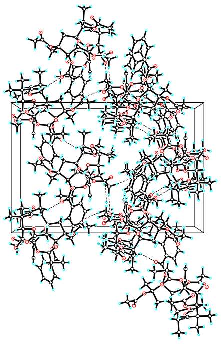

The crystal structure and absolute configuration of **3** were determined by single crystal X-ray diffraction analysis. Crystal data for compound **3**: $C_{38}H_{50}O_{14}$, M = 730.78, a = 11.7516(5) Å, b = 15.0592(7) Å, c = 22.2397(10) Å, α = 90°, β = 90°, γ = 90°, V = 3935.8(3) Å3, T = 100. (2) K, space group P212121, Z = 4, μ(Cu Kα) = 0.783 mm^{-1}, 38614 reflections measured, 7782 independent

reflections (Rint = 0.1520). The final R1 values were 0.0621 (I > 2σ(I)). The final wR(F2) values were 0.1548 (I > 2σ(I)). The final R1 values were 0.0891 (all data). The final wR(F2) values were 0.1790 (all data). The goodness of fit on F2 was 1.046. Flack parameter = -0.10(18). CCDC-2224638 (**3**), contain the supplementary crystallographic data. These data can be obtained free of charge from the Cambridge Crystallographic Data Centre (http://www.ccdc.cam.ac.uk/).

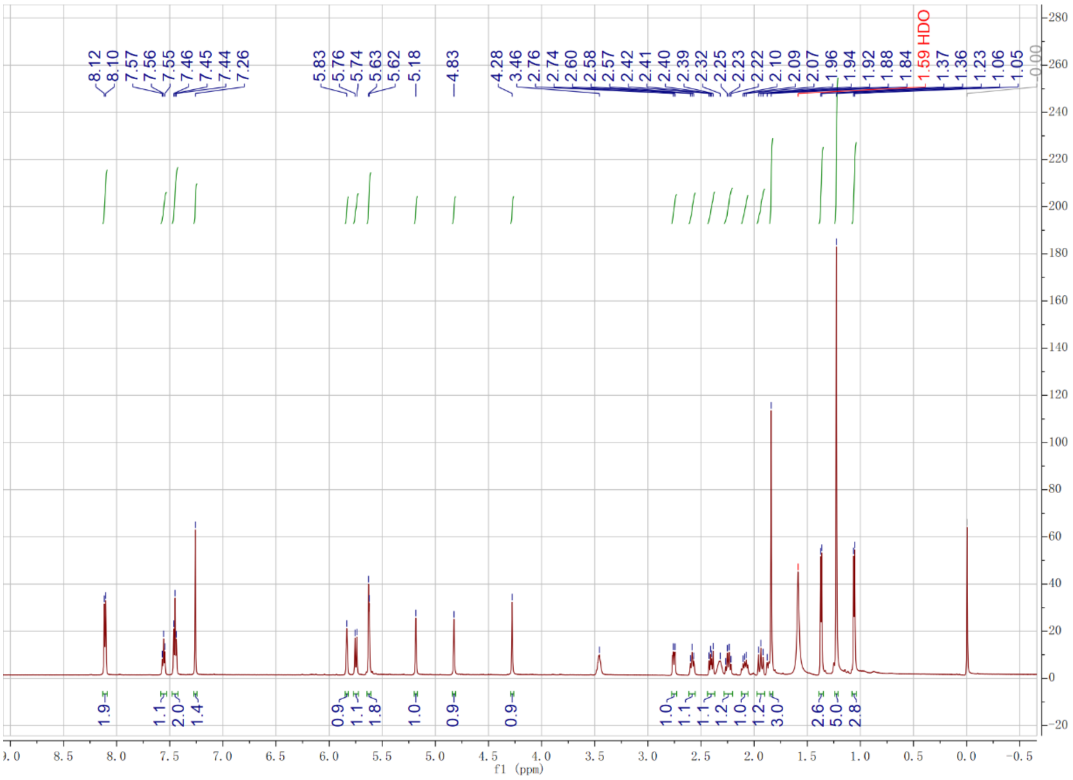

Figure S1. ^{1}H NMR spectrum of euphjatrophane A (**1**)

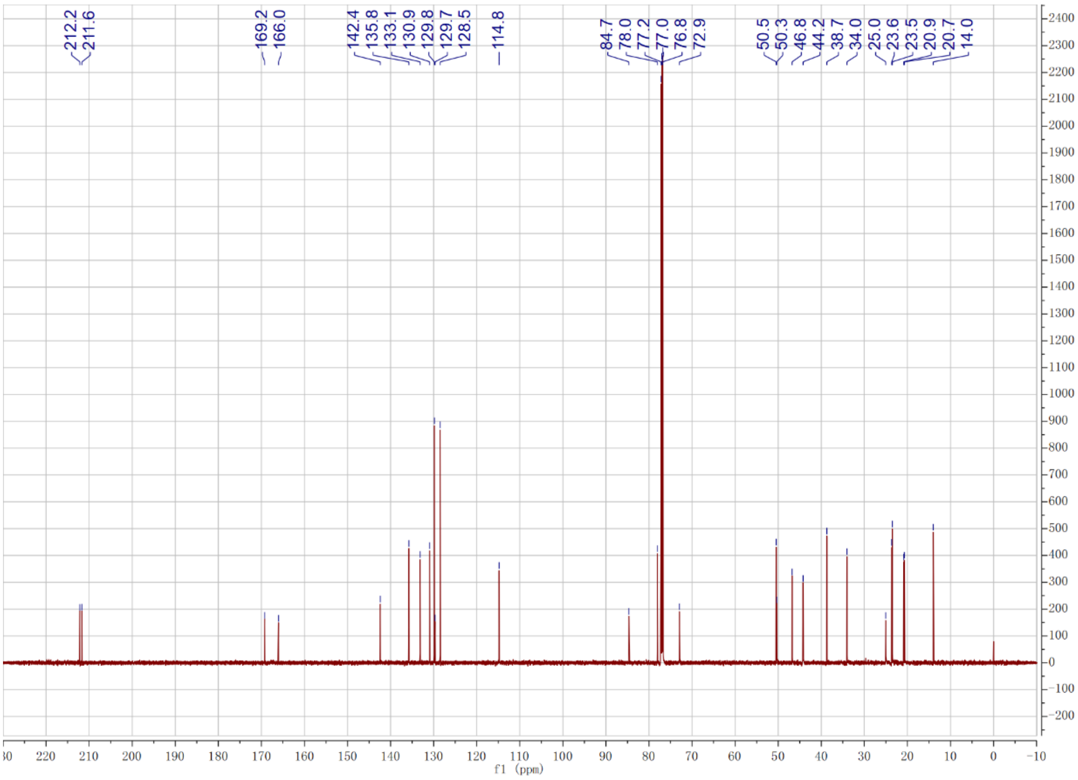

Figure S2. ^{13}C NMR spectrum of euphjatrophane A (**1**)

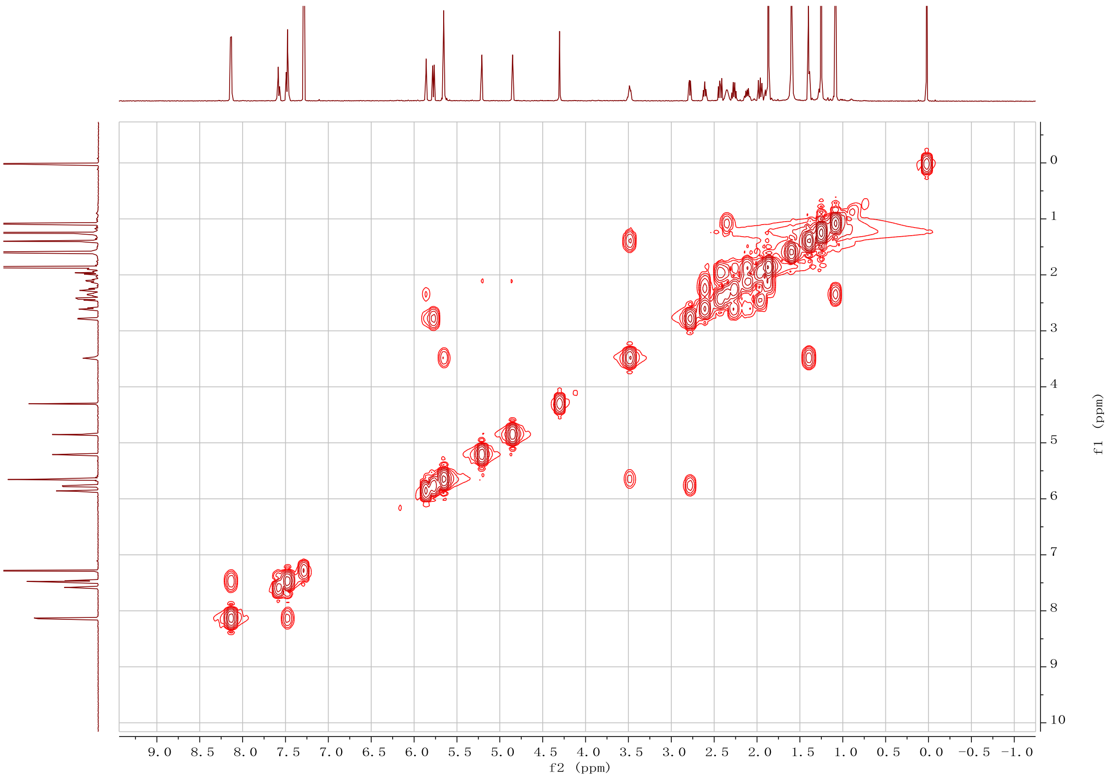

Figure S3. ^{1}H-^{1}H COSY spectrum of euphjatrophane A (**1**)

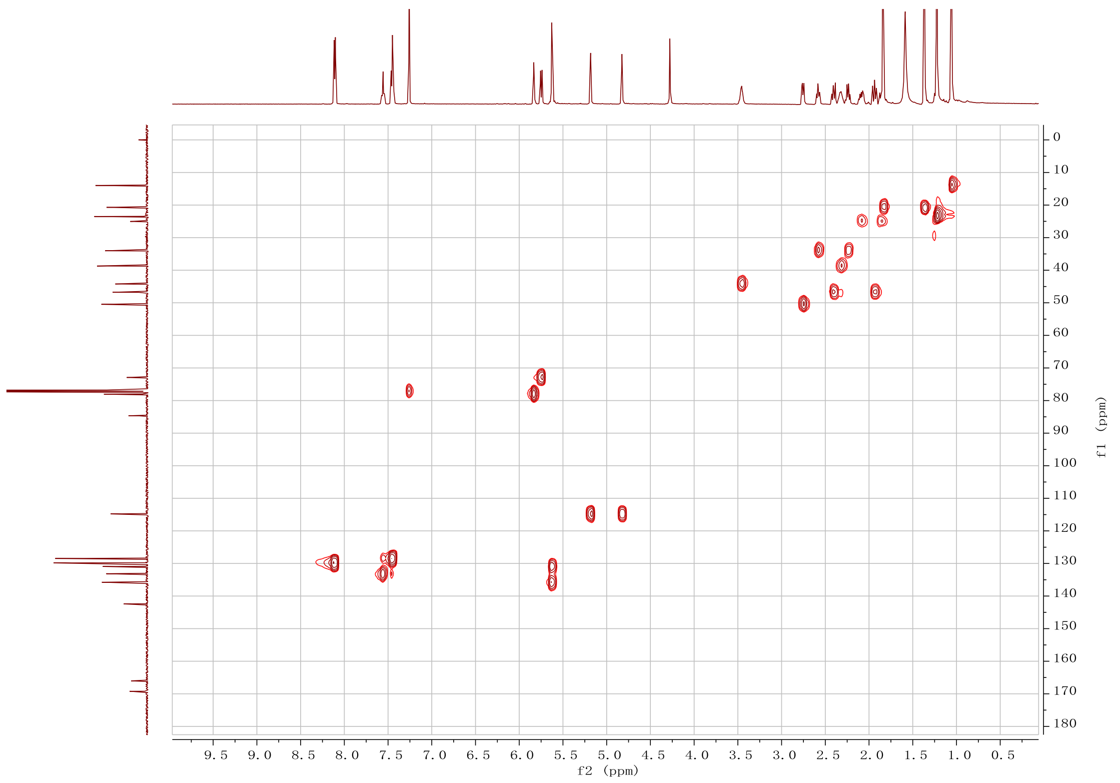

Figure S4. HSQC spectrum of euphjatrophane A (**1**)

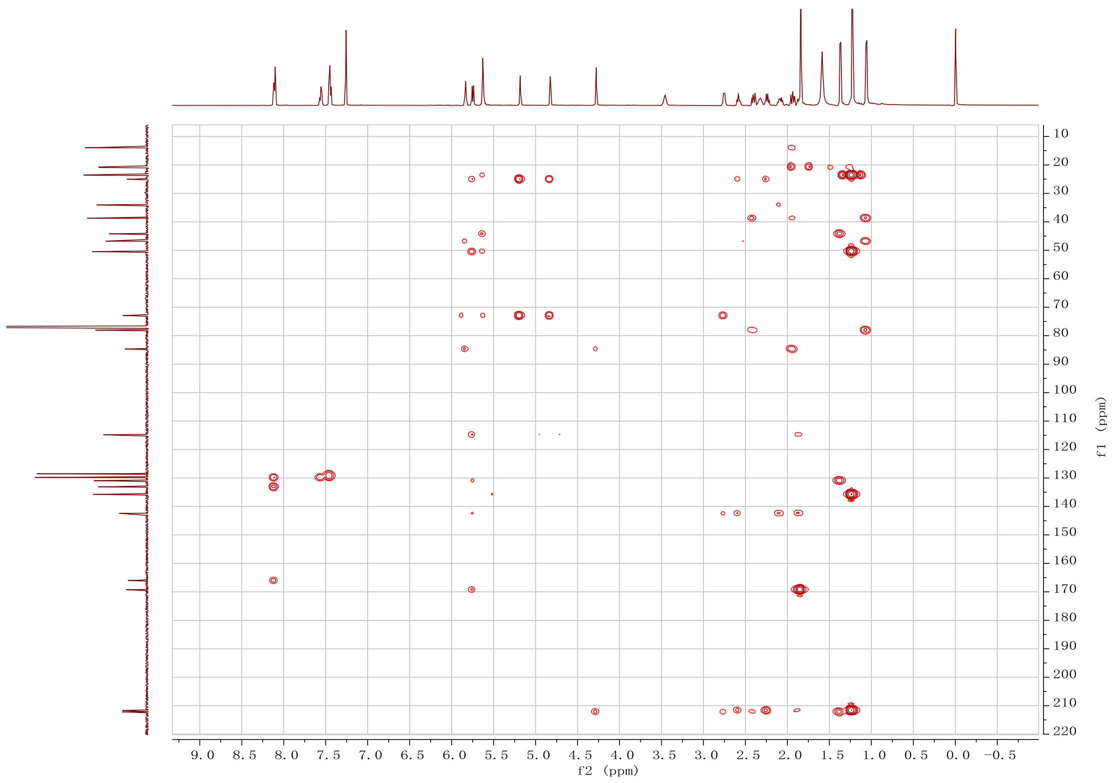

Figure S5. HMBC spectrum of euphjatrophane A (**1**)

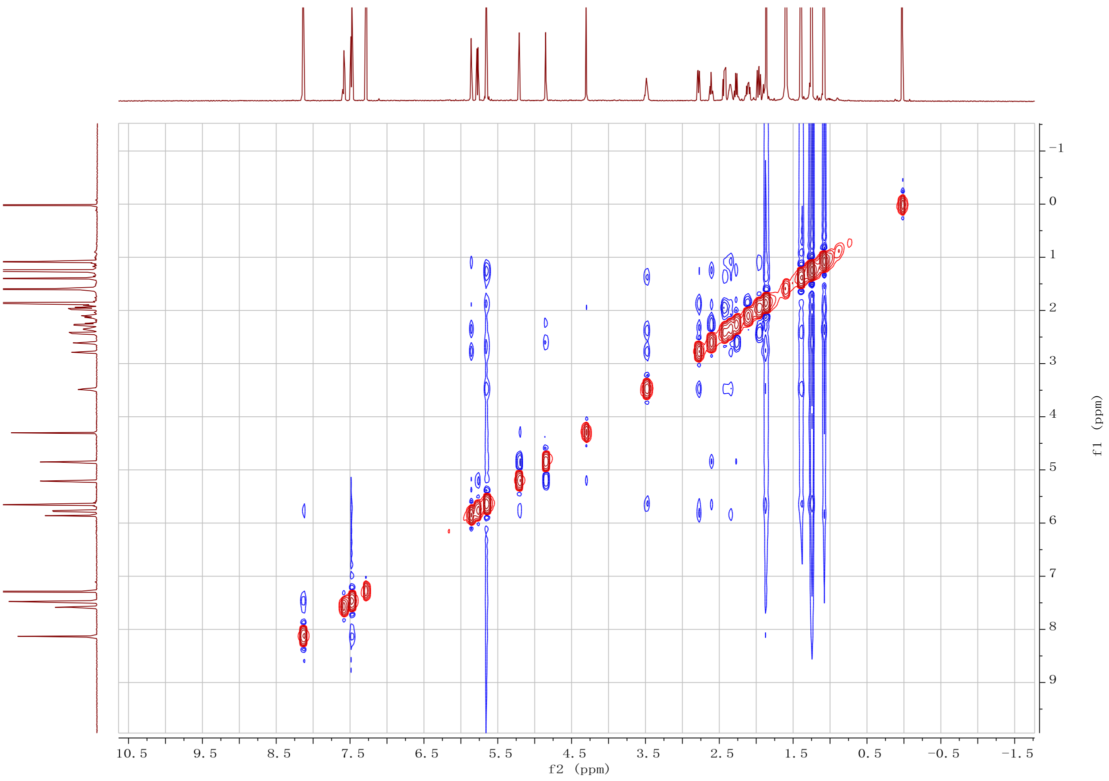

Figure S6. ROESY spectrum of euphjatrophane A (**1**)

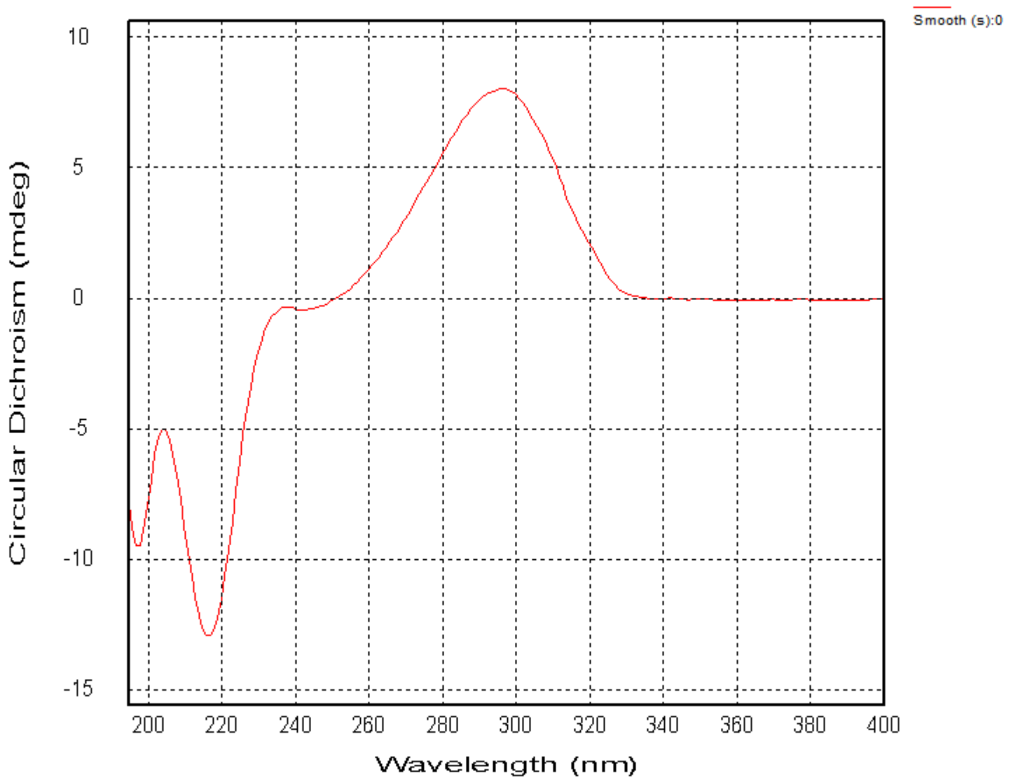

Figure S7. CD spectrum of euphjatrophane A (**1**)

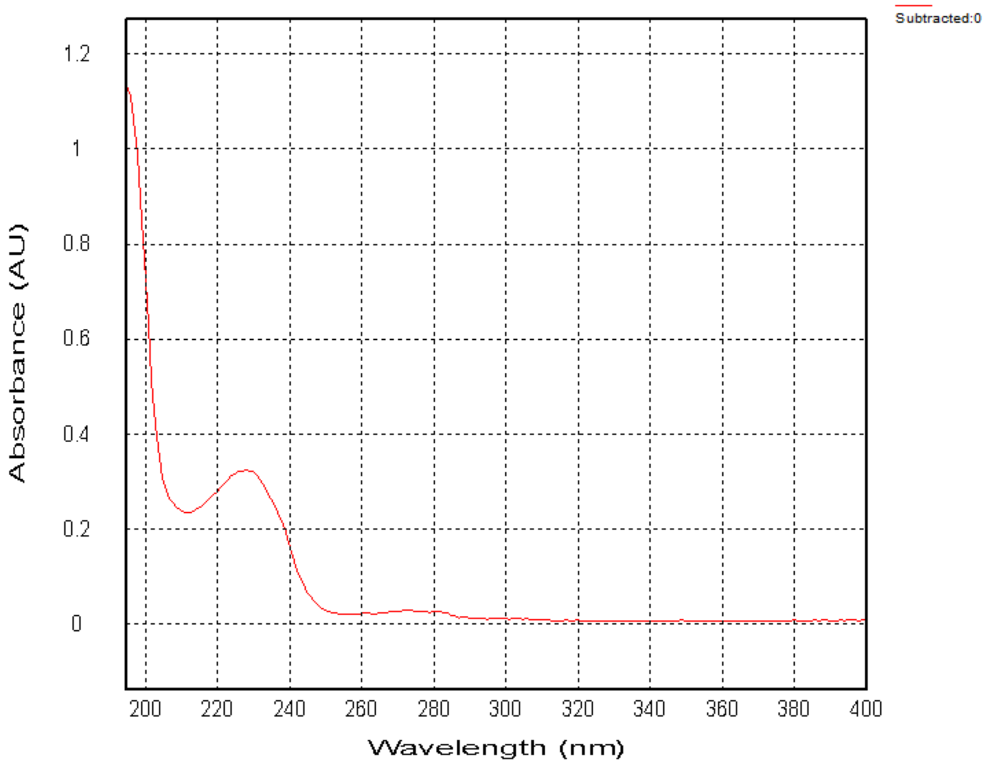

Figure S8. UV spectrum of euphjatrophane A (**1**)

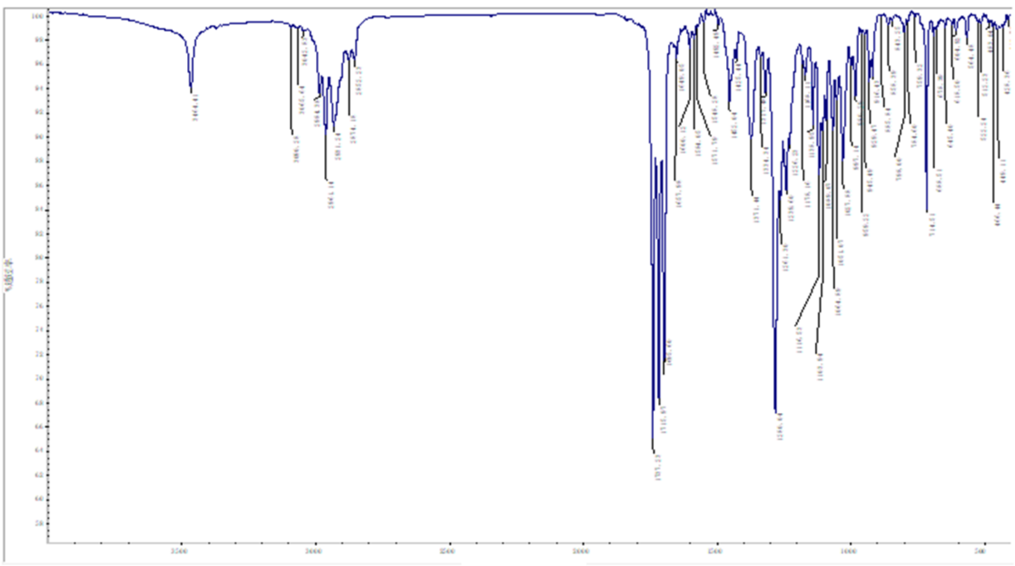

Wavenumber (cm^{-1})

Figure S9. IR spectrum of euphjatrophane A (**1**)

Qualitative Analysis Report

**Data Filename**	HLQY-952.d	**Sample Name**	HLQY-952
**Sample Type**	Sample	**Position**	P1-A3
**Instrument Name**	Instrument 1	**User Name**	
**Acq Method**	s.m	**Acquired Time**	8/31/2020 3:59:52 PM
**IRM Calibration Status**	Success	**DA Method**	Default.m
**Comment**			

Sample Group **Info.**
Acquisition SW 6200 series TOF/6500 series
Version Q-TOF B.05.01 (B5125.2)

User Spectra

Fragmentor Voltage	Collision Energy	Ionization Mode
135	0	ESI

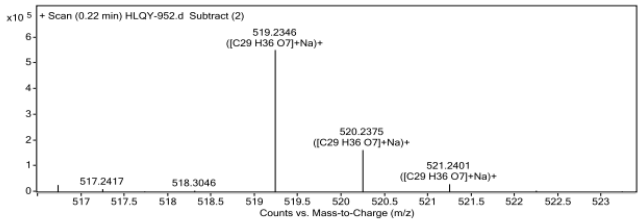

Peak List

m/z	z	Abund	Formula	Ion
274.2735	1	187302.42		
318.2996	1	199051.88		
437.2319	1	820976		
438.2345	1	230957.72		
514.2788	1	538790		
519.2346	1	551515.94	C29 H36 O7	(M+Na)+
520.2375	1	164931.64	C29 H36 O7	(M+Na)+
535.2074	1	173966.48		
1015.479	1	333128.03		
1016.4812	1	214466.94		

Formula Calculator Element Limits

Element	Min	Max
C	3	60
H	0	120
O	0	30

Formula Calculator Results

Formula	CalculatedMass	CalculatedMz	Mz	Diff. (mDa)	Diff. (ppm)	DBE
C29 H36 O7	496.2461	519.2353	519.2346	0.70	1.35	12.0000

--- End Of Report ---

Figure S10. (+)-HRESIMS spectrum of euphjatrophane A (**1**)

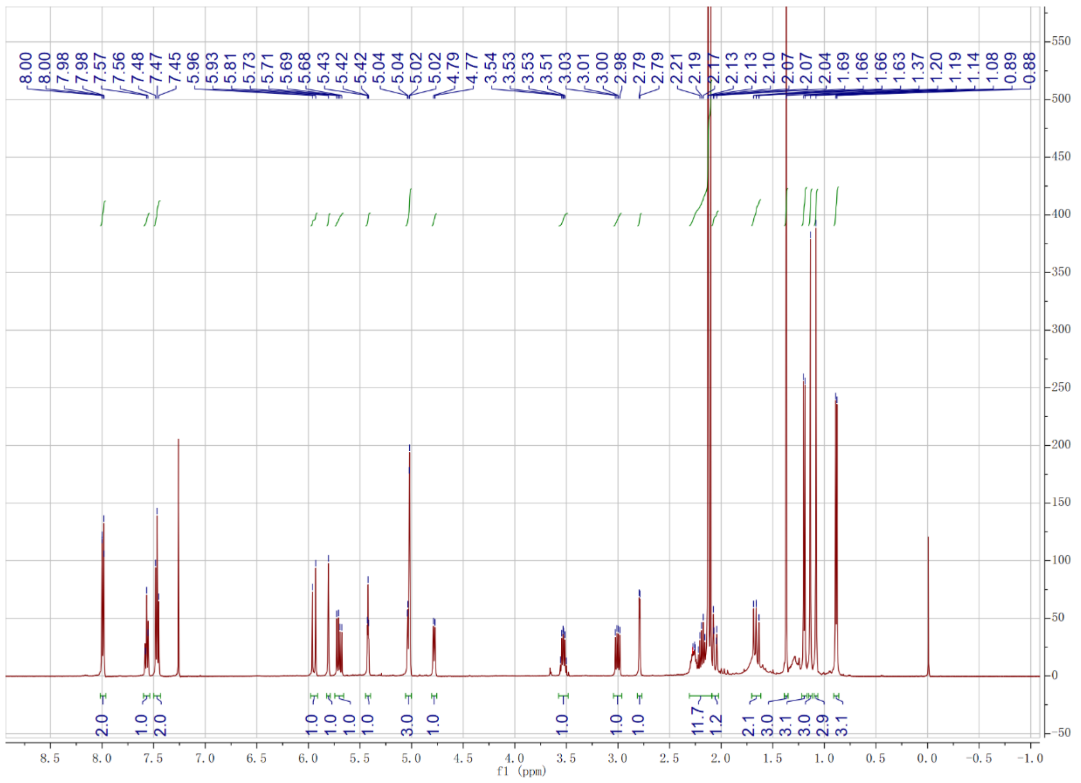

Figure S11. ^{1}H NMR spectrum of euphjatrophane B (**2**)

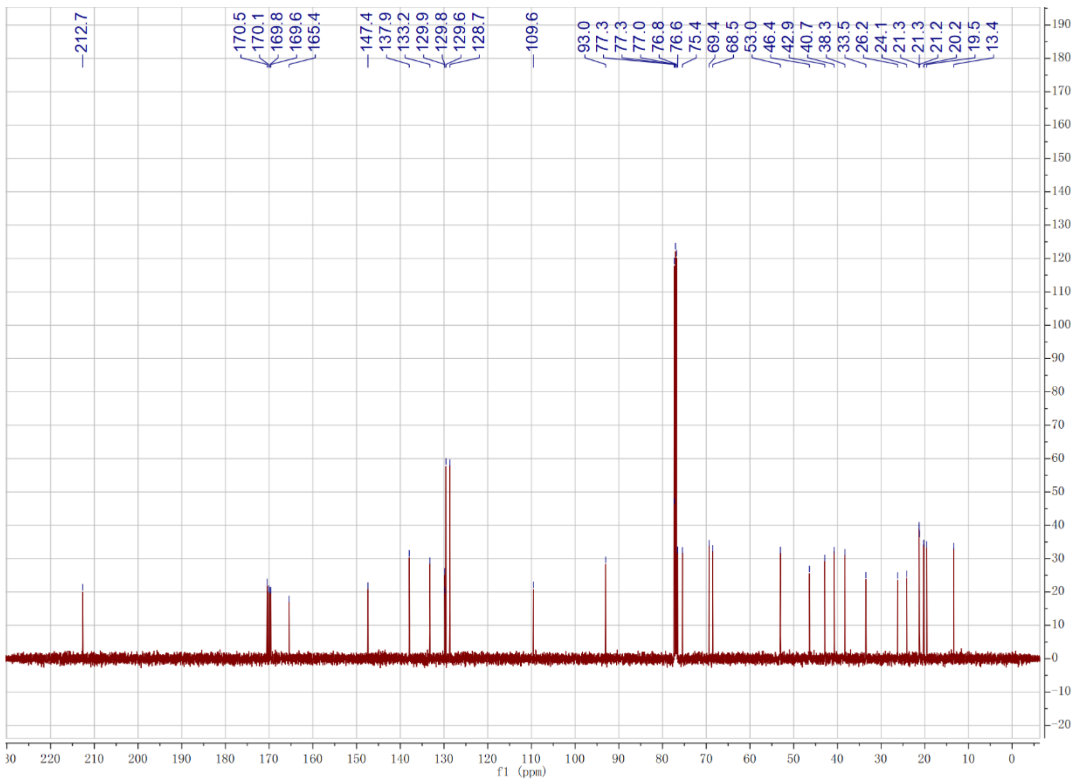

Figure S12. ^{13}C NMR spectrum of euphjatrophane B (**2**)

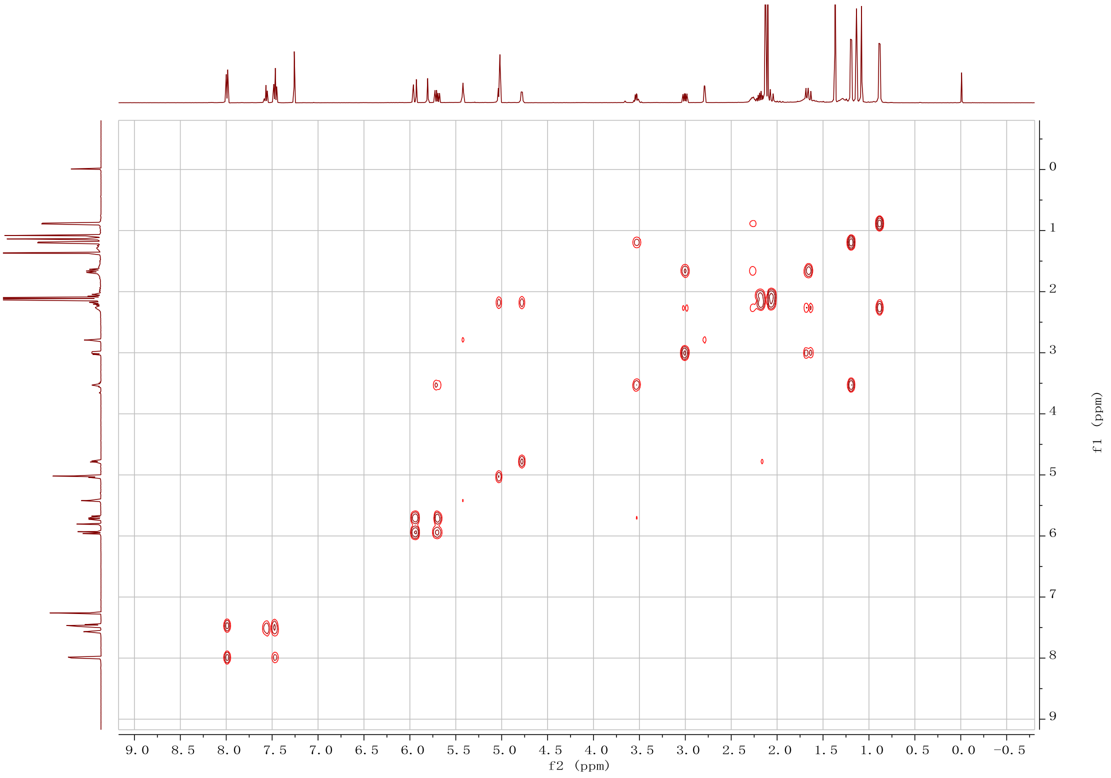

Figure S13. ^{1}H-^{1}H COSY spectrum of euphjatrophane B (**2**)

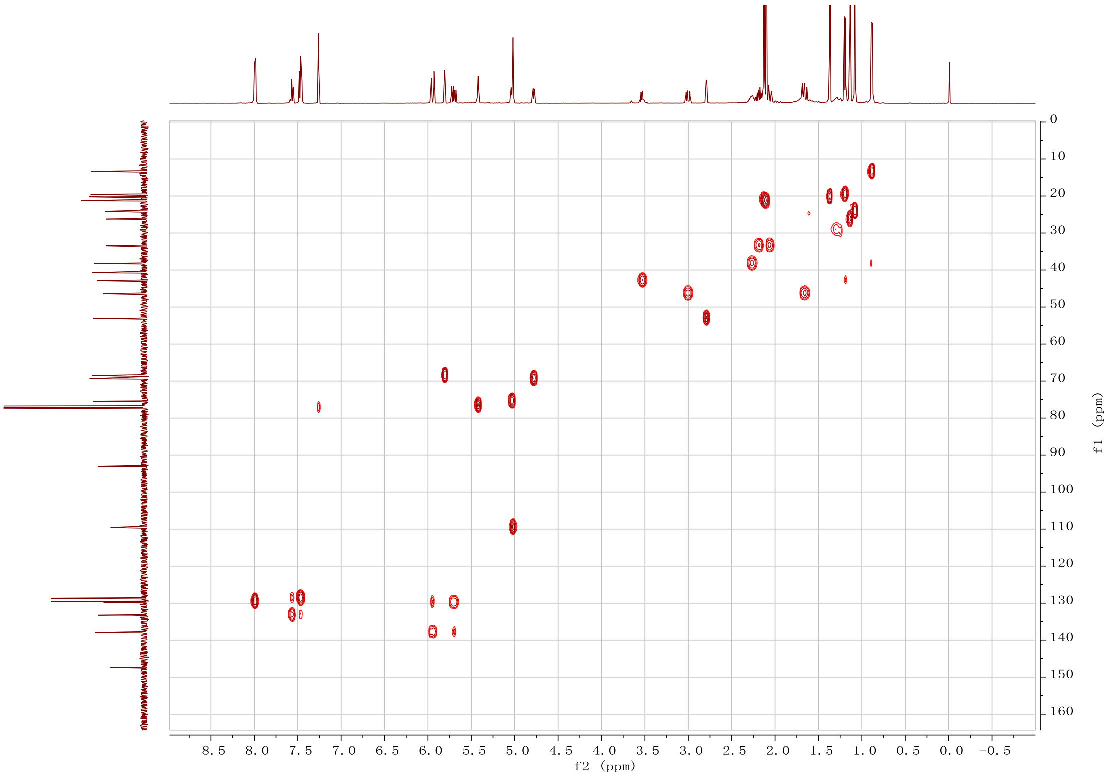

Figure S14. HSQC spectrum of euphjatrophane B (**2**)

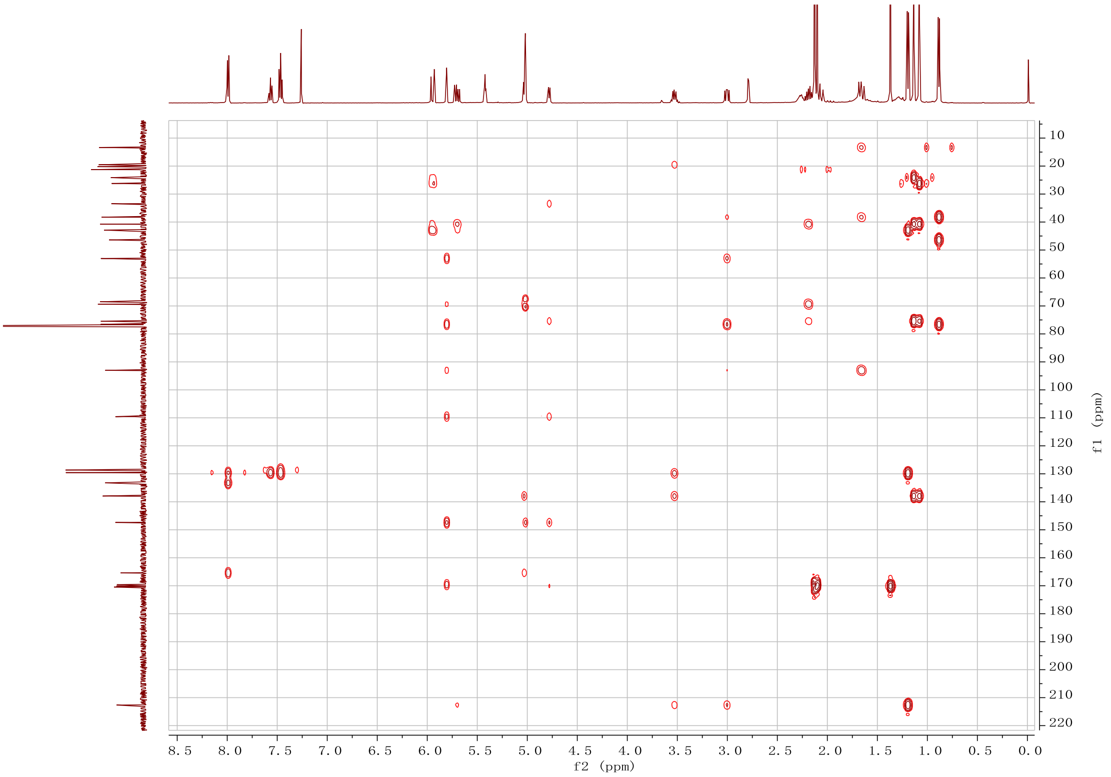

Figure S15. HMBC spectrum of euphjatrophane B (**2**)

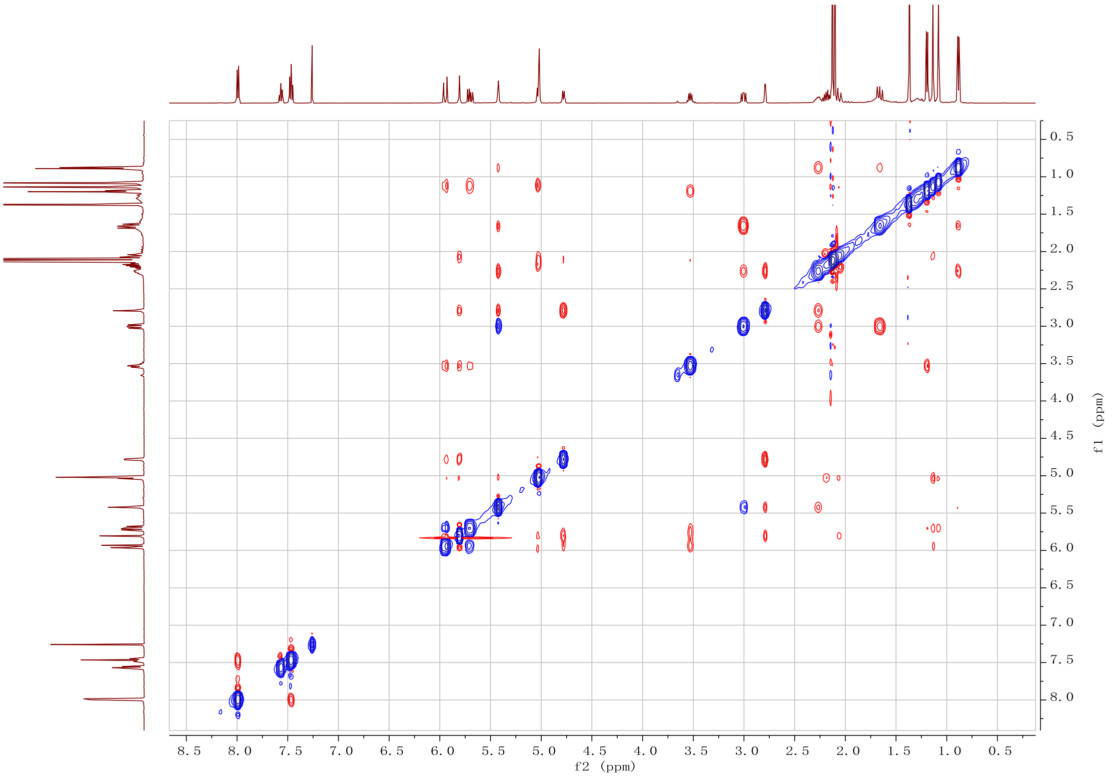

Figure S16. ROESY spectrum of euphjatrophane B (**2**)

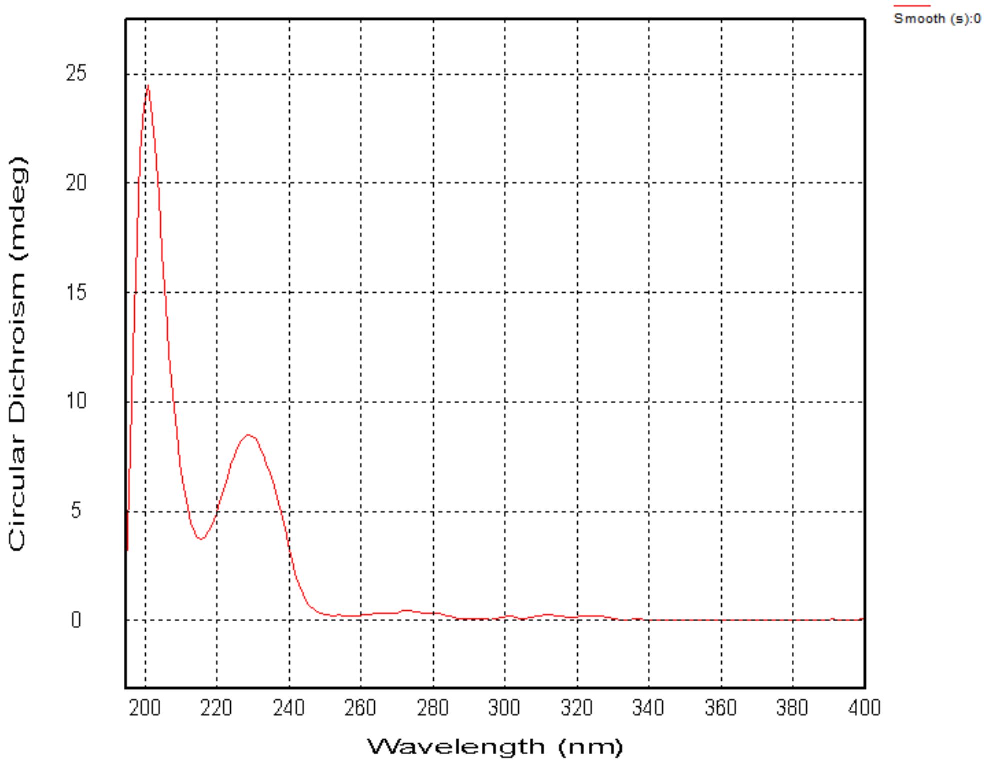

Figure S17. CD spectrum of euphjatrophane B (**2**)

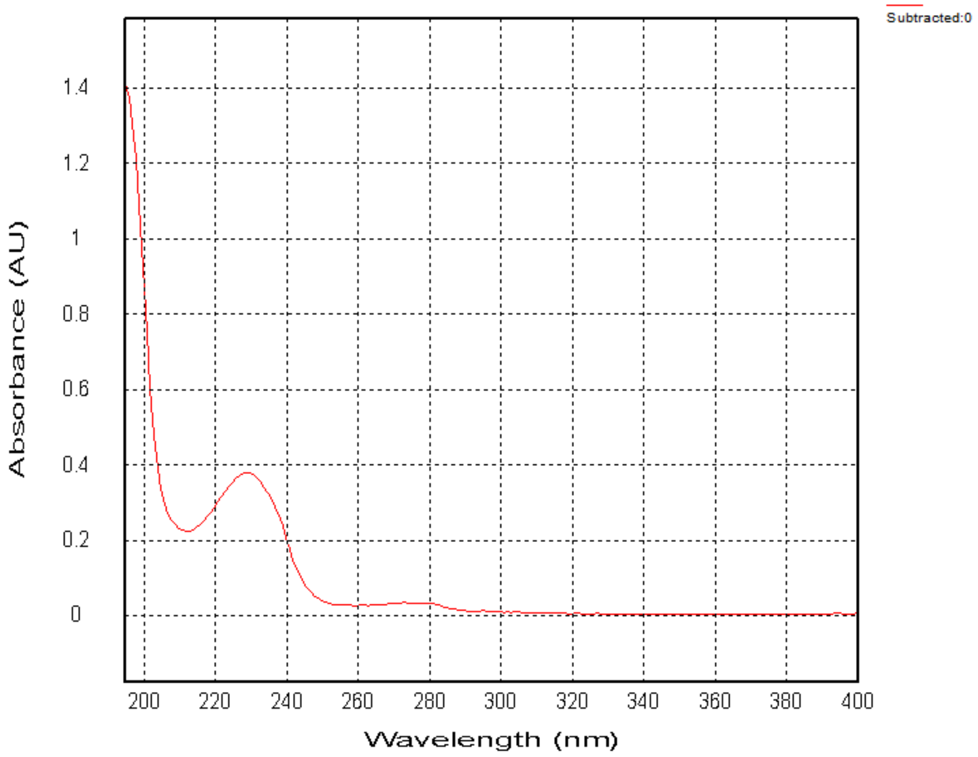

Figure S18. UV spectrum of euphjatrophane B (**2**)

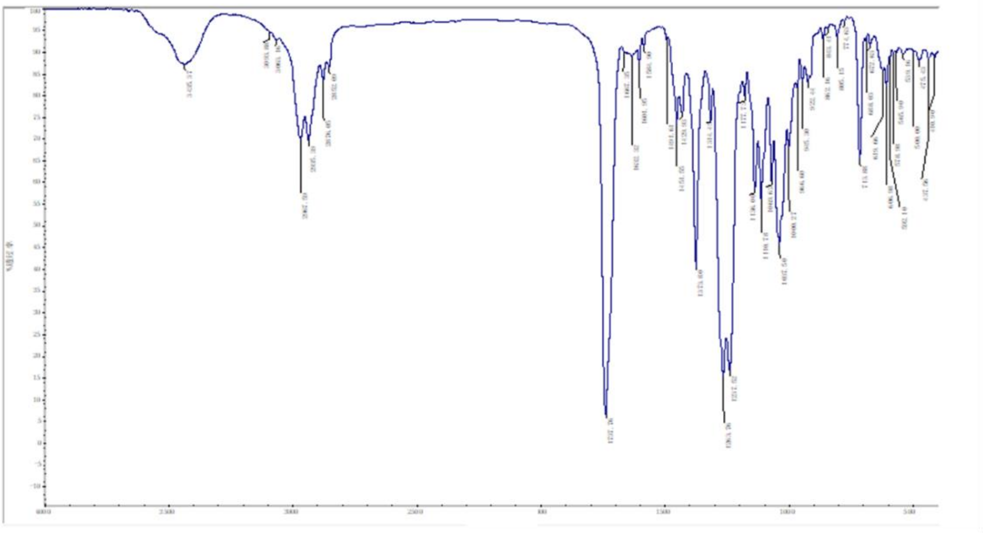

Wavenumber (cm-1)

Figure S19. IR spectrum of euphjatrophane B (**2**)

Qualitative Analysis Report

**Data Filename**	HLQY-506.d	**Sample Name**	HLQY-506
**Sample Type**	Sample	**Position**	P1-A1
**Instrument Name**	Instrument 1	**User Name**	
**Acq Method**	s.m	**Acquired Time**	8/31/2020 3:57:19 PM
**IRM Calibration Status**	Success	**DA Method**	Default.m
**Comment**			

Sample Group **Info.**
Acquisition SW 6200 series TOF/6500 series
Version Q-TOF B.05.01 (B5125.2)

User Spectra

Fragmentor Voltage	Collision Energy	Ionization Mode
135	0	ESI

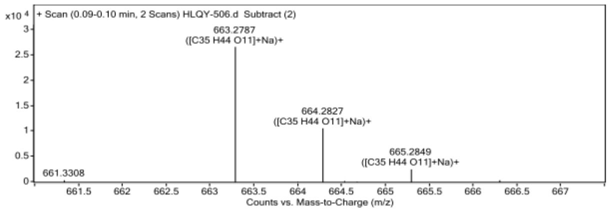

Peak List

m/z	z	Abund	Formula	Ion
581.2744	1	4388.82		
658.3233	1	19267.56		
659.3239	1	7405.69		
663.2787	1	26662.53	C35 H44 O11	(M+Na)+
664.2827	1	10650.82	C35 H44 O11	(M+Na)+
679.2511	1	10730.54		
680.254	1	4275.27		
686.3542	1	5421.47		
1303.5697	1	5857.36		
1304.5701	1	4597.56		

Formula Calculator Element Limits

Element	Min	Max
C	3	60
H	0	120
O	0	30

Formula Calculator Results

Formula	CalculatedMass	CalculatedMz	Mz	Diff. (mDa)	Diff. (ppm)	DBE
C35 H44 O11	640.2884	663.2776	663.2787	-1.10	-1.66	14.0000

--- End Of Report ---

Figure S20. (+)-HRESIMS spectrum of euphjatrophane B (**2**)

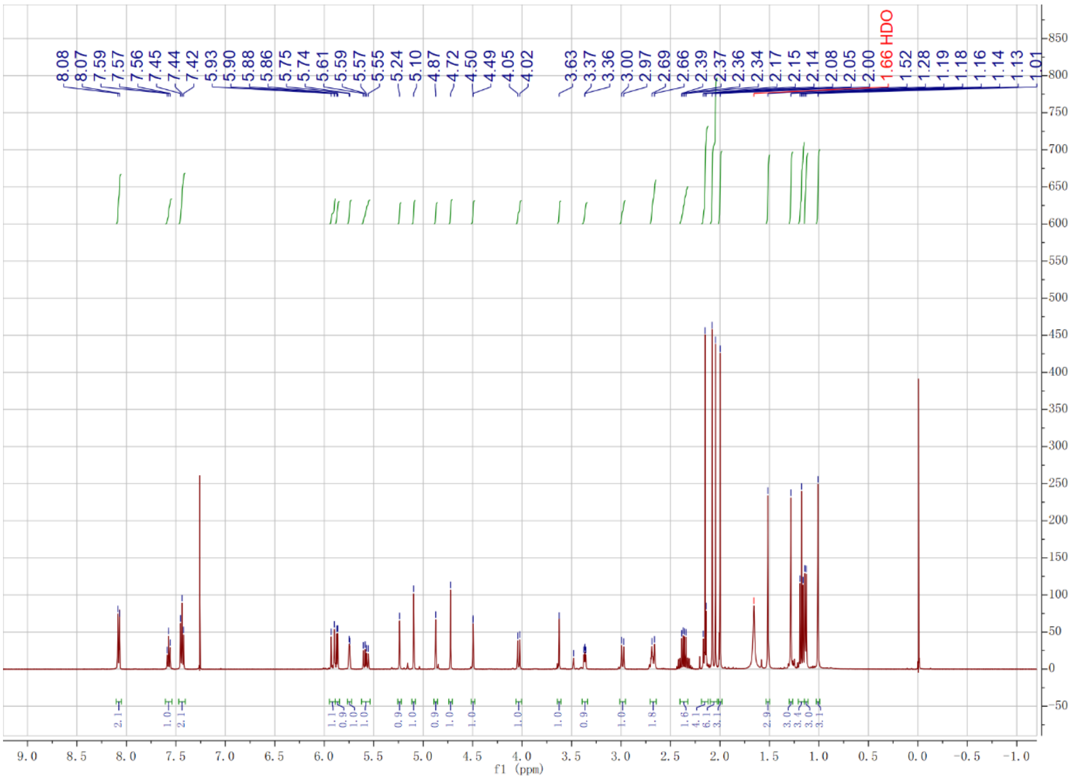

Figure S21. ¹H NMR spectrum of euphpepluone G (**3**)

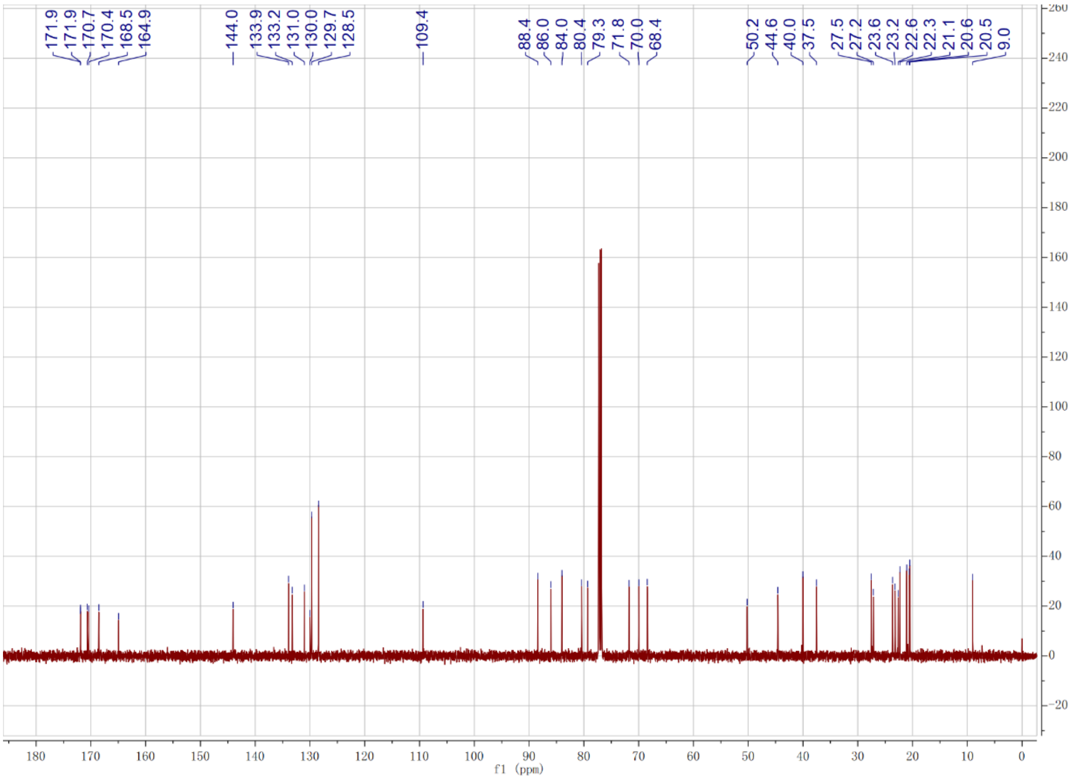

Figure S22. ¹³C NMR spectrum of euphpepluone G (**3**)

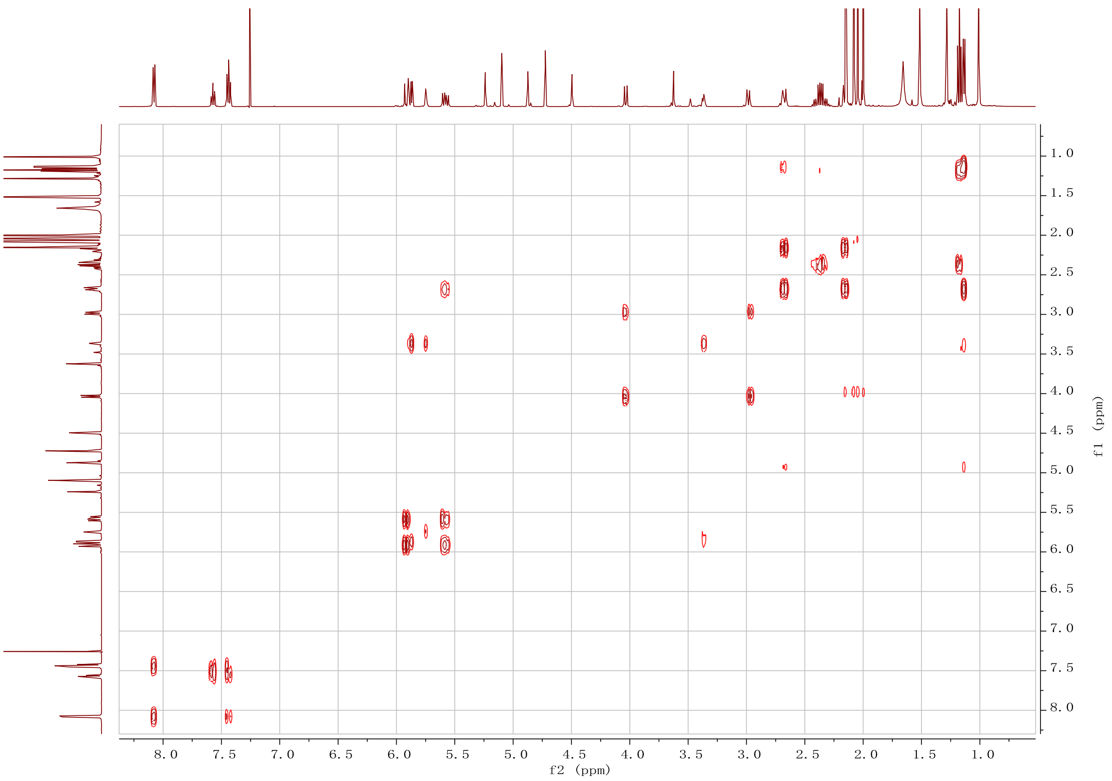

Figure S23. ^{1}H-^{1}H COSY spectrum of euphpepluone G (**3**)

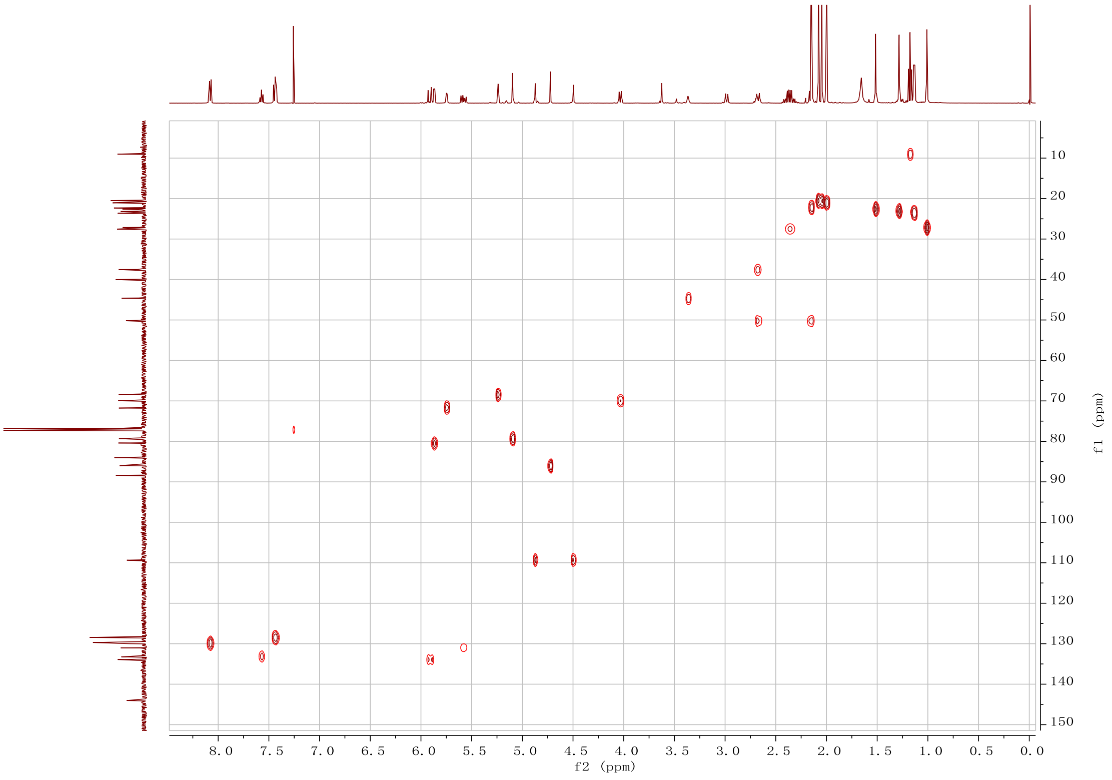

Figure S24. HSQC spectrum of euphpepluone G (**3**)

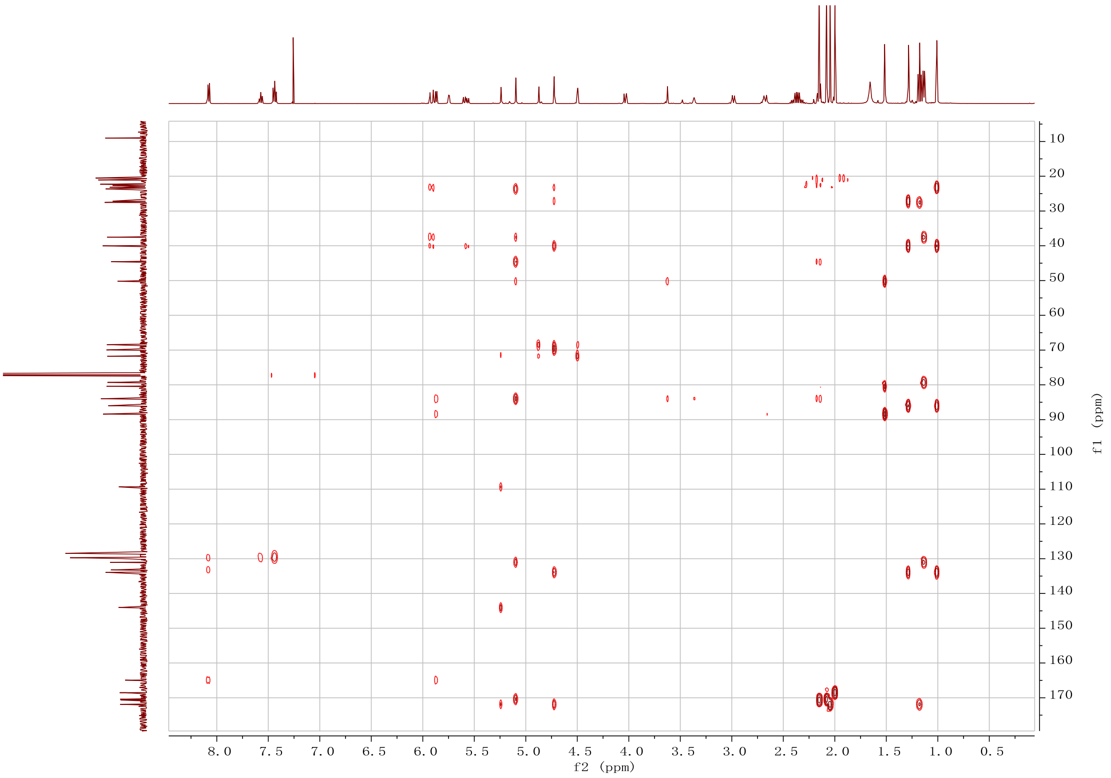

Figure S25. HMBC spectrum of euphpepluone G (**3**)

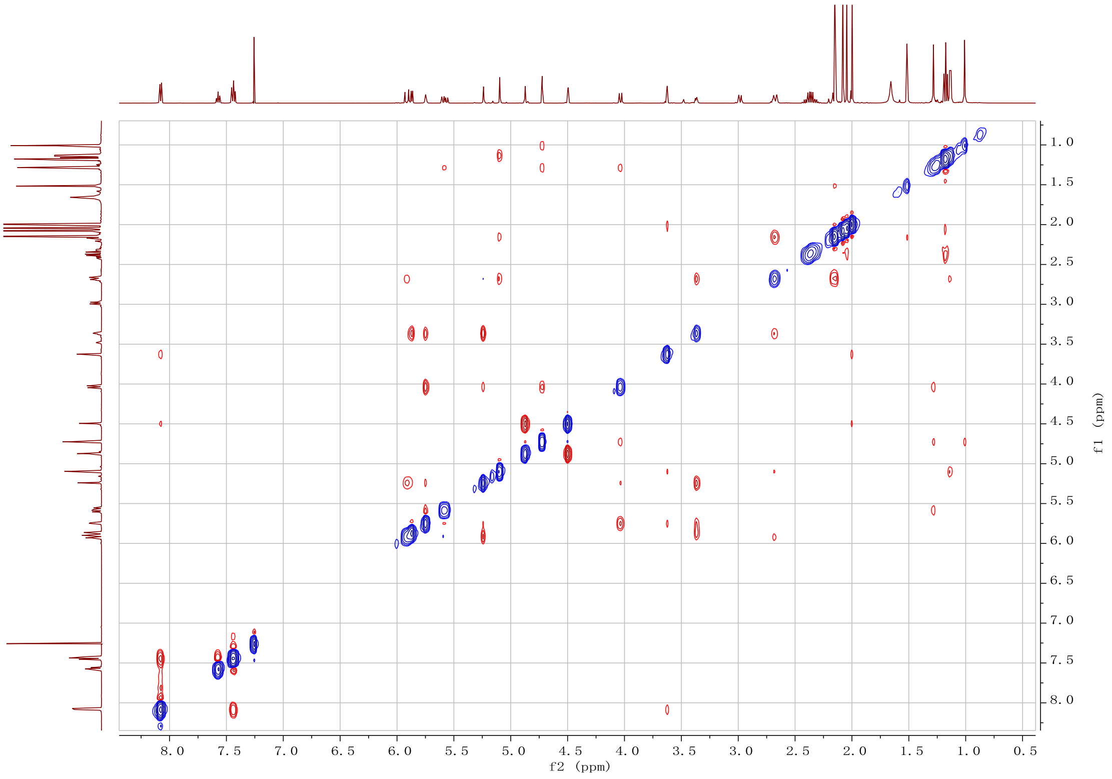

Figure S26.ROESY spectrum of euphpepluone G (**3**)

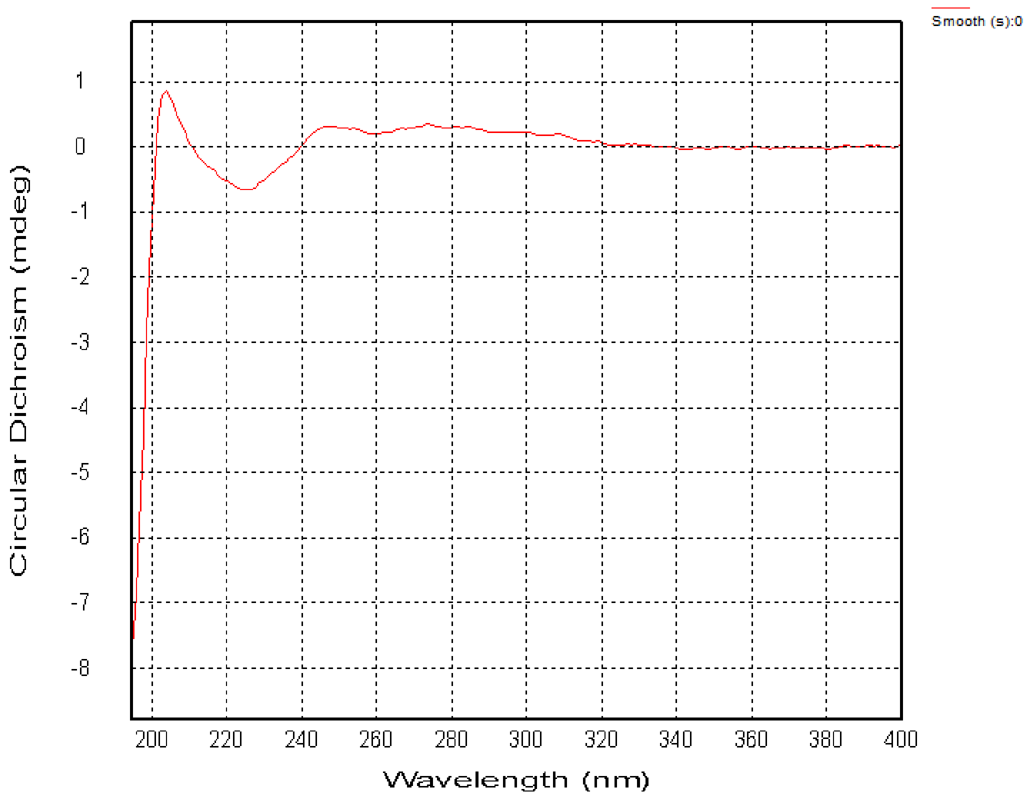

Figure S27. CD spectrum of euphpepluone G (**3**)

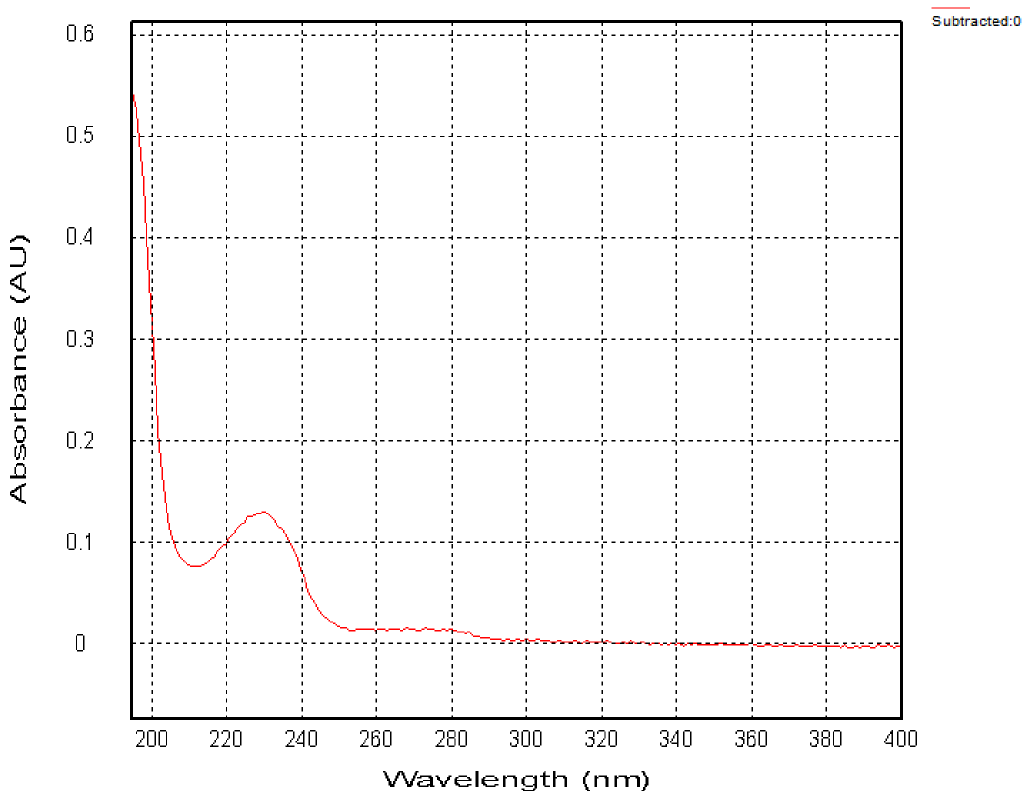

Figure S28. UV spectrum of euphpepluone G (**3**)

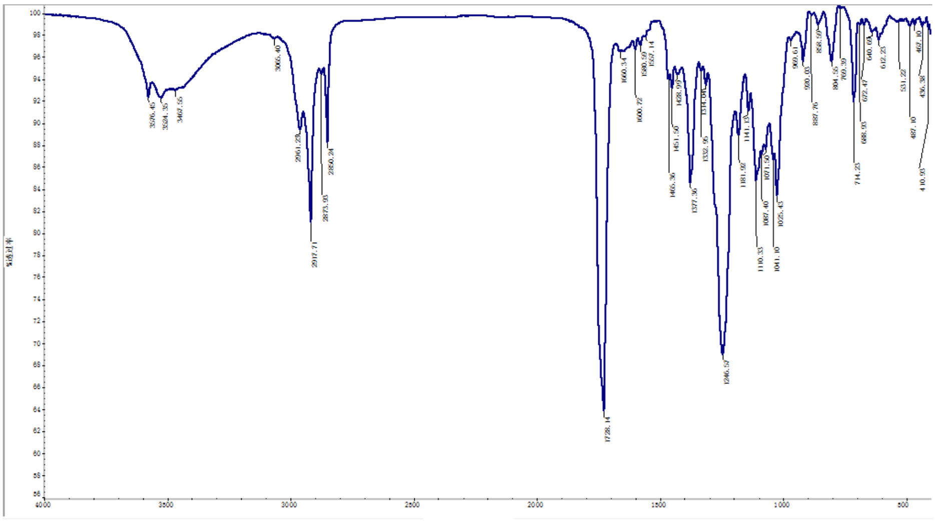

Wavenumber (cm^{-1})

Figure S29. IR spectrum of euphpepluone G (**3**)

Qualitative Analysis Report

**Data Filename**	HLQY-864.d	**Sample Name**	HLQY-864
**Sample Type**	Sample	**Position**	P1-A8
**Instrument Name**	Instrument 1	**User Name**	
**Acq Method**	s.m	**Acquired Time**	8/31/2020 4:06:17 PM
**IRM Calibration Status**	Success	**DA Method**	Default.m
**Comment**			

**Sample Group**		**Info.**
**Acquisition SW Version**	6200 series TOF/6500 series Q-TOF B.05.01 (B5125.2)	

User Spectra

Fragmentor Voltage	Collision Energy	Ionization Mode
135	0	ESI

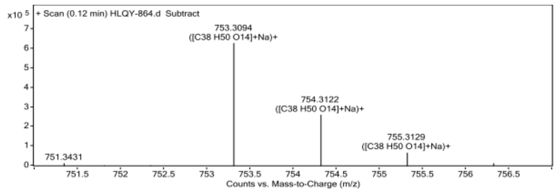

Peak List

m/z	z	Abund	Formula	Ion
713.3164	1	157386.44		
748.3539	1	479388.53		
749.3575	1	197872.16		
753.3094	1	628769.69	C38 H50 O14	(M+Na)+
754.3122	1	260881.48	C38 H50 O14	(M+Na)+
755.3129	1	68288.34	C38 H50 O14	(M+Na)+
769.2835	1	77974.2		
1483.6283	1	174450.08		
1484.6319	1	146220.5		
1485.6352	1	67339.73		

Formula Calculator Element Limits

Element	Min	Max
C	3	60
H	0	120
O	0	30

Formula Calculator Results

Formula	CalculatedMass	CalculatedMz	Mz	Diff. (mDa)	Diff. (ppm)	DBE
C38 H50 O14	730.3201	753.3093	753.3094	-0.10	-0.13	14.0000

--- End Of Report ---

Figure S30. (+)-HRESIMS spectrum of euphpepluone G (**3**)

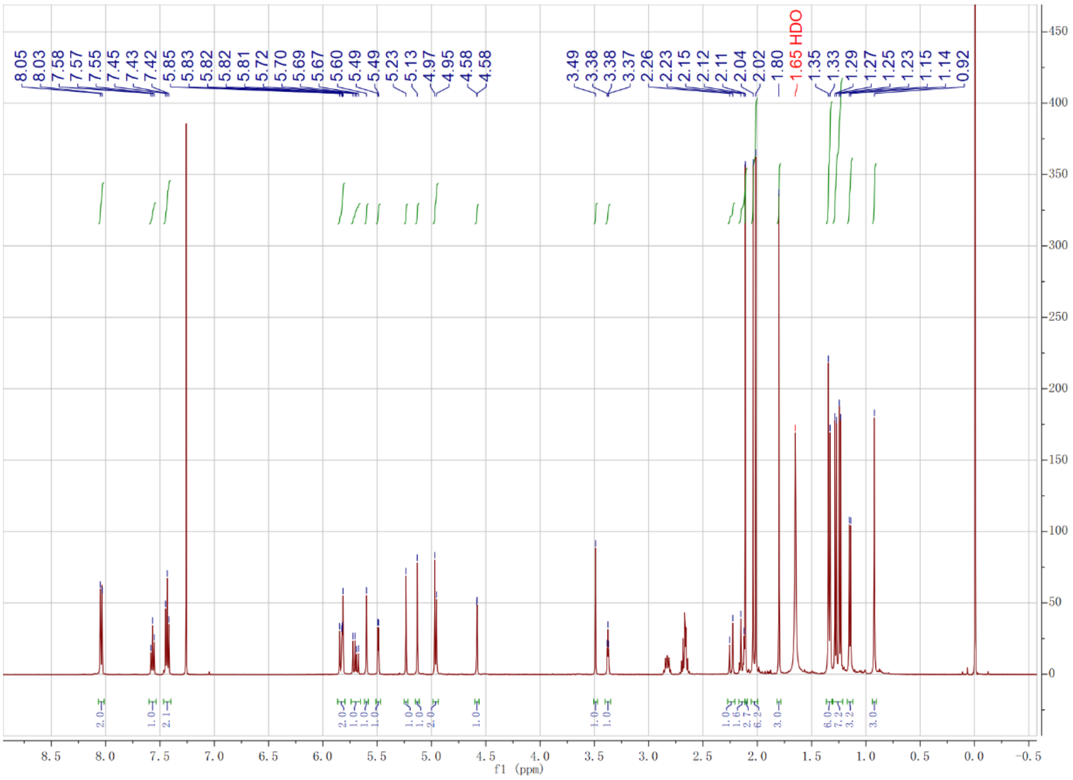

Figure S31. ^{1}H NMR spectrum of euphjatrophane C (**4**)

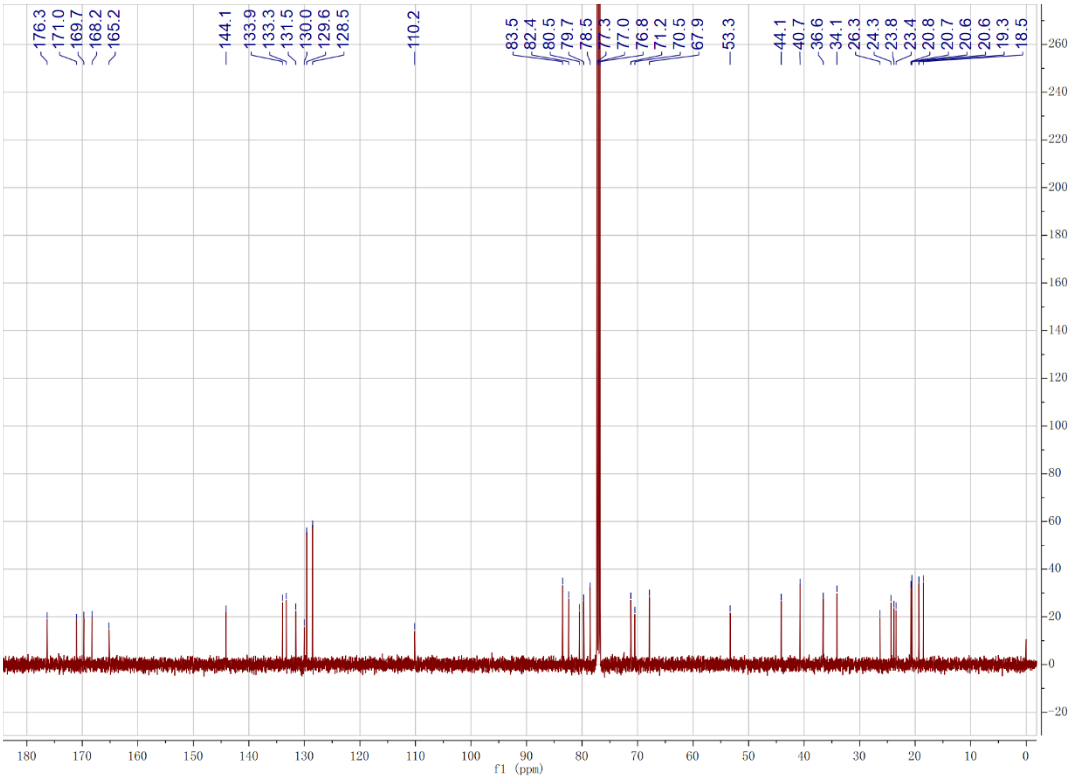

Figure S32. ^{13}C NMR spectrum of euphjatrophane C (**4**)

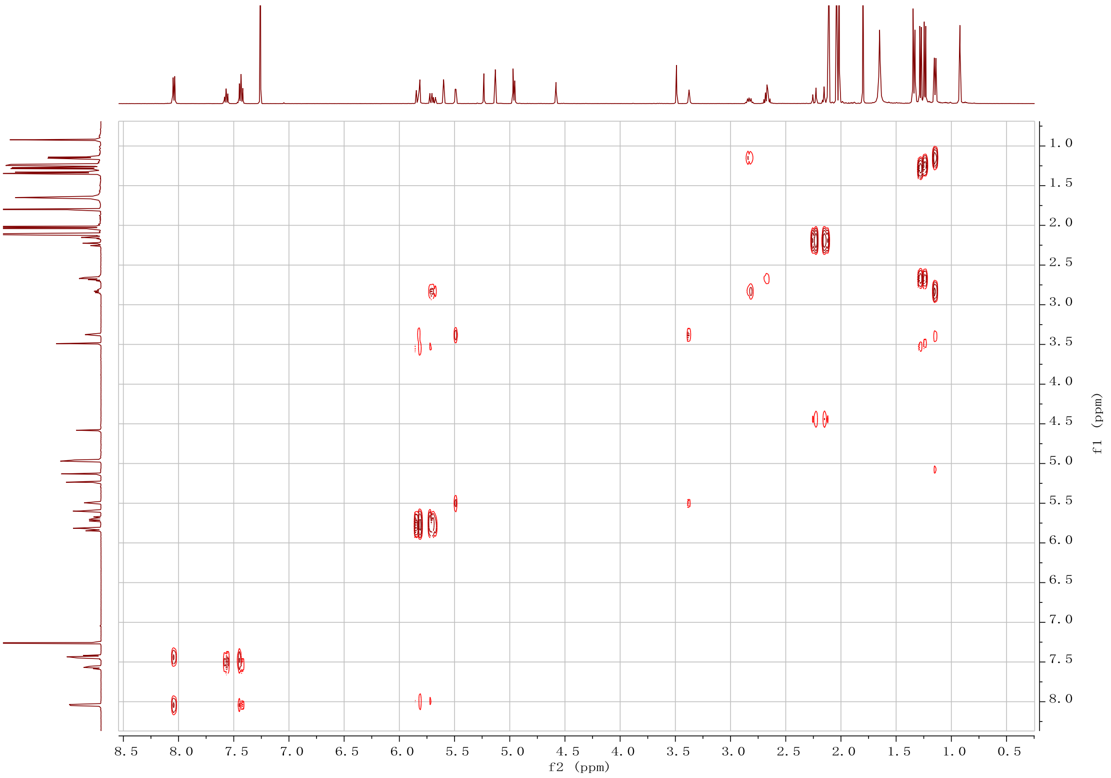

Figure S33. ^{1}H-^{1}H COSY spectrum of euphjatrophane C (**4**)

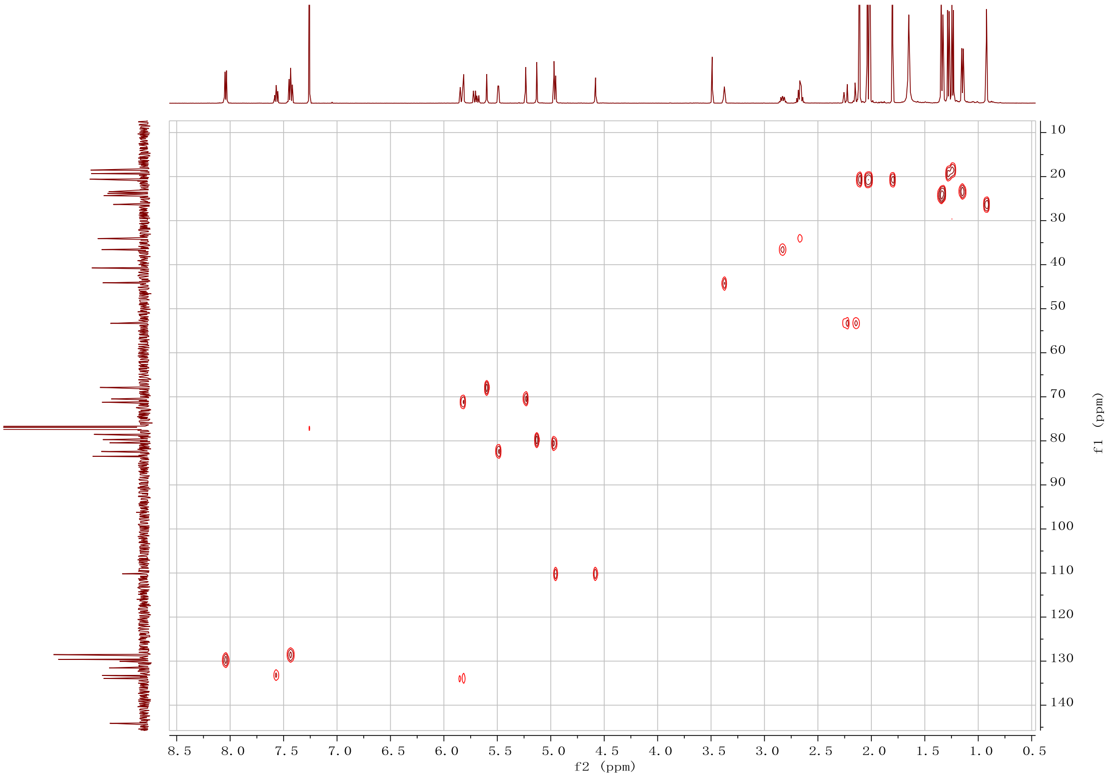

Figure S34. HSQC spectrum of euphjatrophane C (**4**)

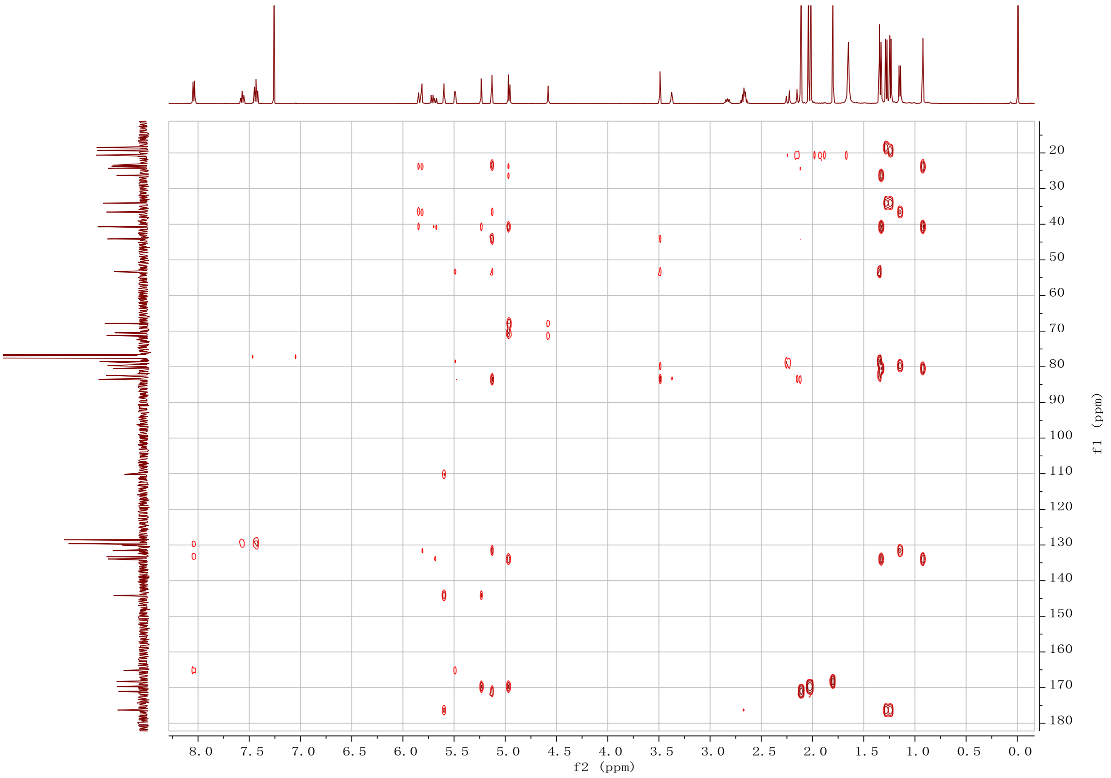

Figure S35. HMBC spectrum of euphjatrophane C (**4**)

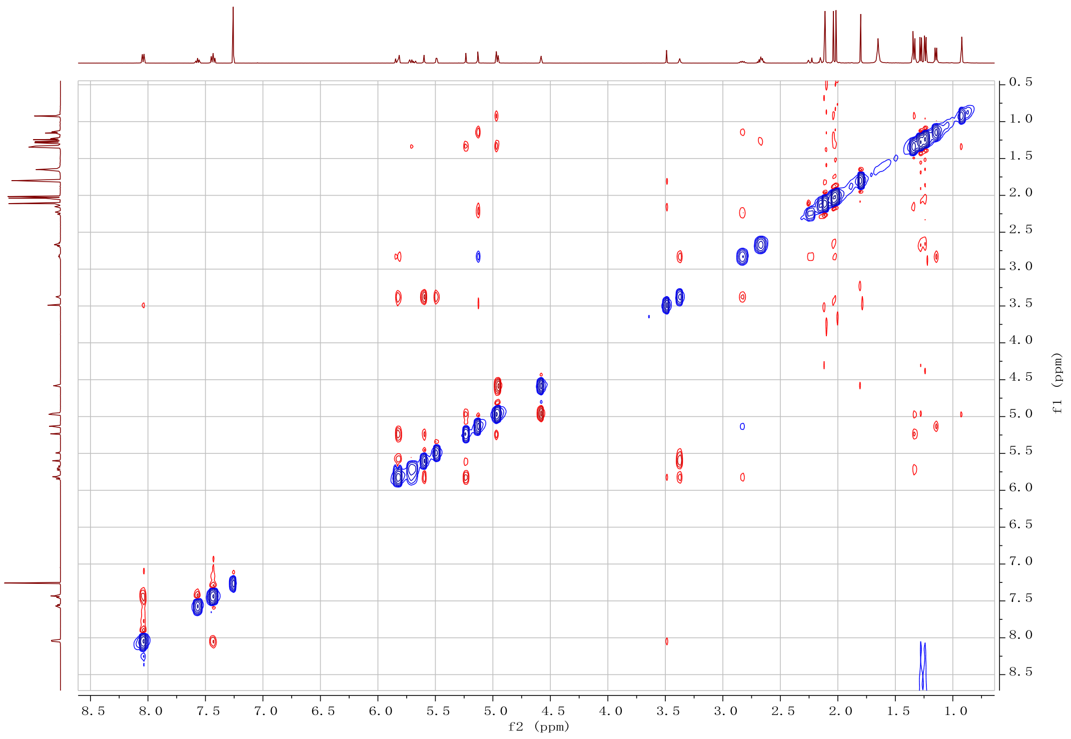

Figure S36. ROESY spectrum of euphjatrophane C (**4**)

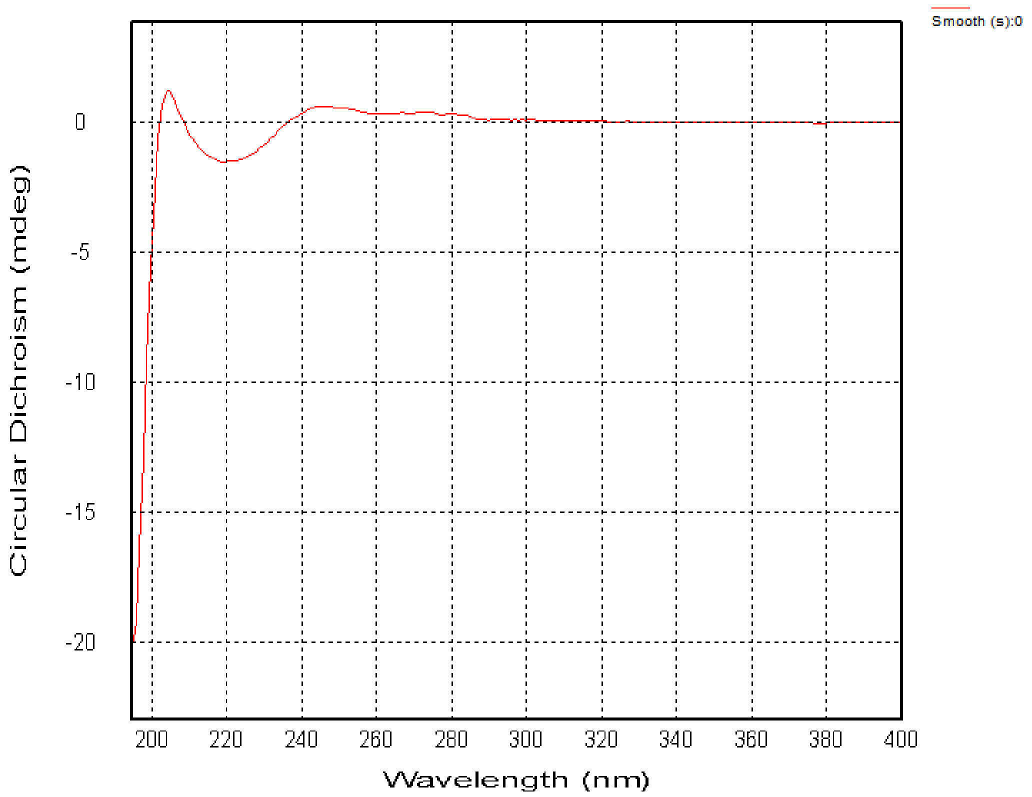

Figure S37. CD spectrum of euphjatrophane C (**4**)

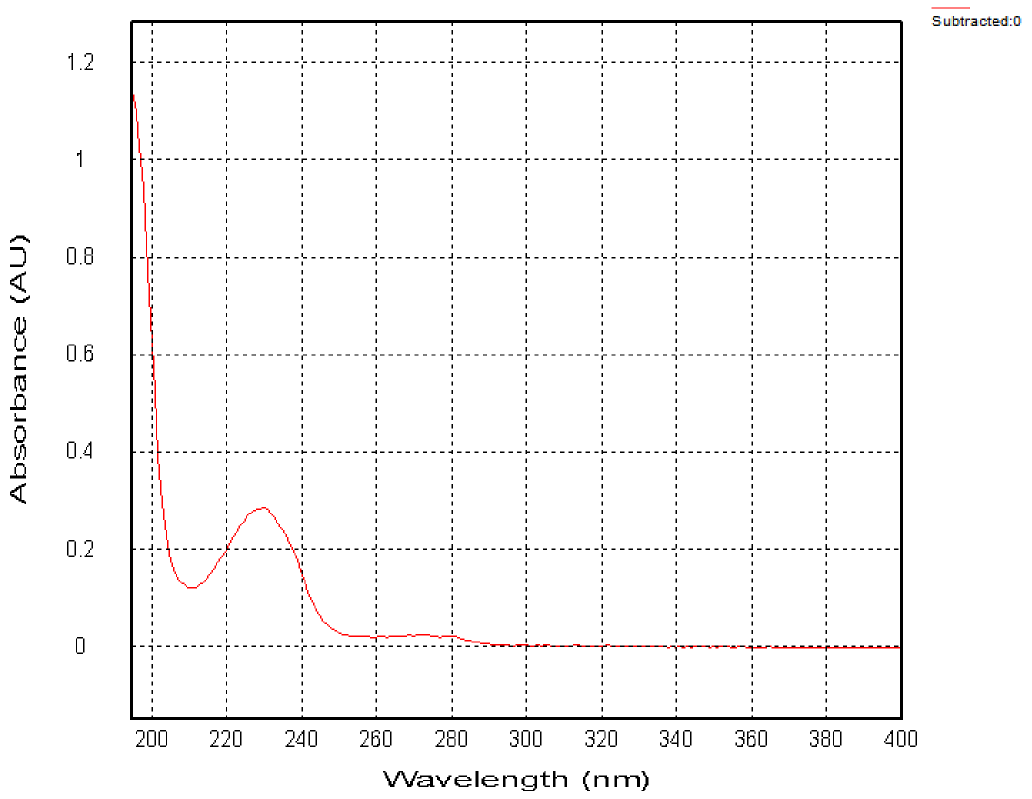

Figure S38. UV spectrum of euphjatrophane C (**4**)

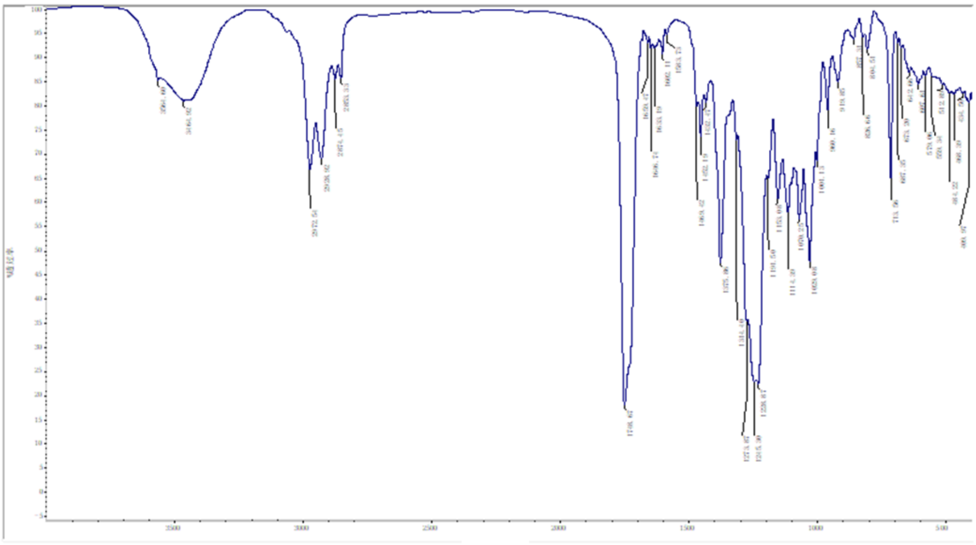

Wavenumber (cm^{-1})

Figure S39. IR spectrum of euphjatrophane C (**4**)

Qualitative Analysis Report

**Data Filename**	HLQY-881.d	**Sample Name**	HLQY-881
**Sample Type**	Sample	**Position**	P1-A6
**Instrument Name**	Instrument 1	**User Name**	
**Acq Method**	s.m	**Acquired Time**	7/29/2020 10:53:45 AM
**IRM Calibration Status**	Success	**DA Method**	Default.m
**Comment**			

Sample Group **Info.**
Acquisition SW Version 6200 series TOF/6500 series Q-TOF B.05.01 (B5125.2)

User Spectra

Fragmentor Voltage	Collision Energy	Ionization Mode
135	0	ESI

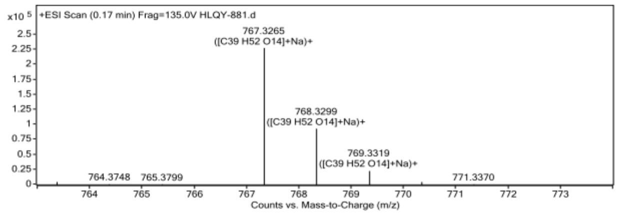

Peak List

m/z	z	Abund	Formula	Ion
102.1279	1	1552532.13		
103.1313	1	96506.15		
304.1548	1	11476.21		
317.2411	1	9243.8		
762.3707	1	11510.65		
767.3265	1	227820.47	C39 H52 O14	(M+Na)+
768.3299	1	93488.44	C39 H52 O14	(M+Na)+
769.3319	1	23889.52	C39 H52 O14	(M+Na)+
783.3002	1	29571.4		
784.3043	1	14502.56		

Formula Calculator Element Limits

Element	Min	Max
C	3	60
H	0	120
O	0	30

Formula Calculator Results

Formula	CalculatedMass	CalculatedMz	Mz	Diff. (mDa)	Diff. (ppm)	DBE
C39 H52 O14	744.3357	767.3249	767.3265	-1.60	-2.09	14.0000

--- End Of Report ---

Figure S40. (+)-HRESIMS spectrum of euphjatrophane C (**4**)

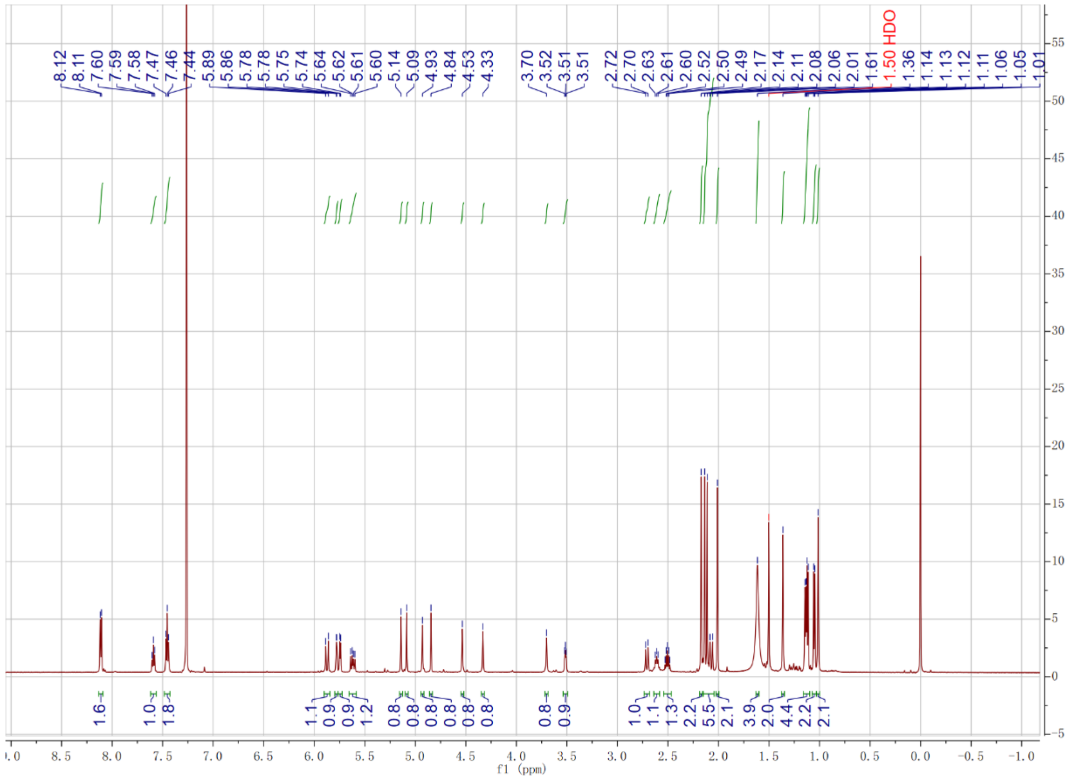

Figure S41. ^{1}H NMR spectrum of euphjatrophane D (**5**)

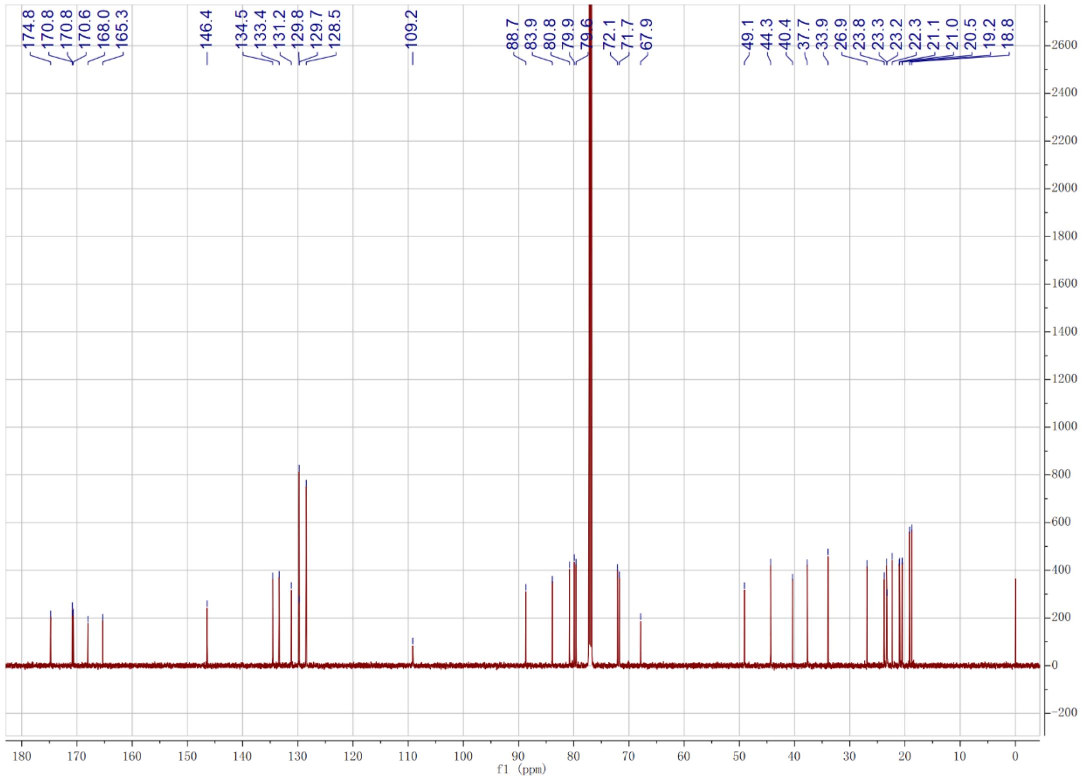

Figure S42. ^{13}C NMR spectrum of euphjatrophane D (**5**)

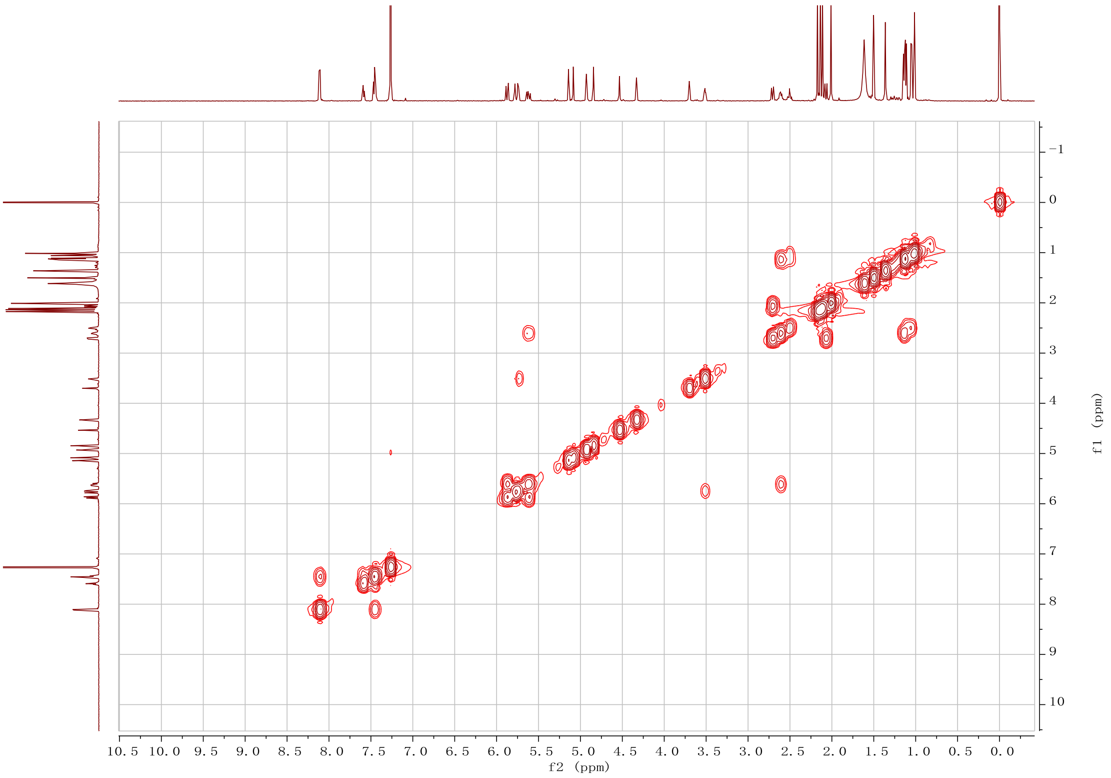

Figure S43. ^{1}H-^{1}H COSY spectrum of euphjatrophane D (**5**)

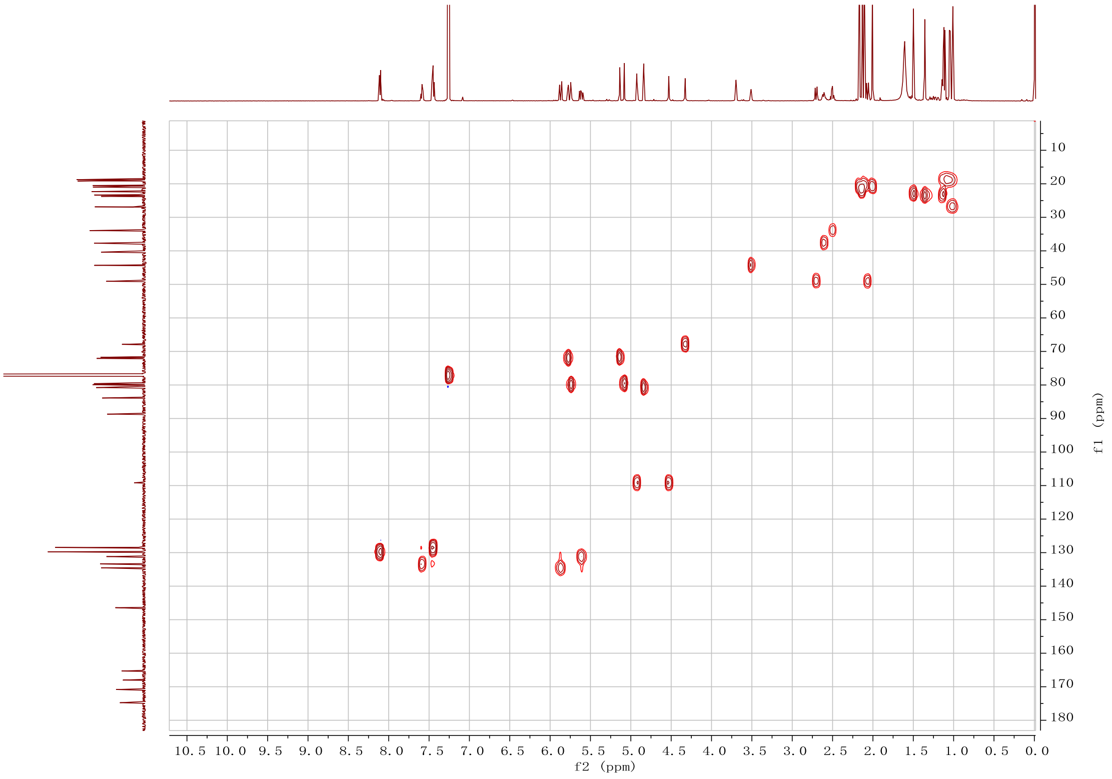

Figure S44. HSQC spectrum of euphjatrophane D (**5**)

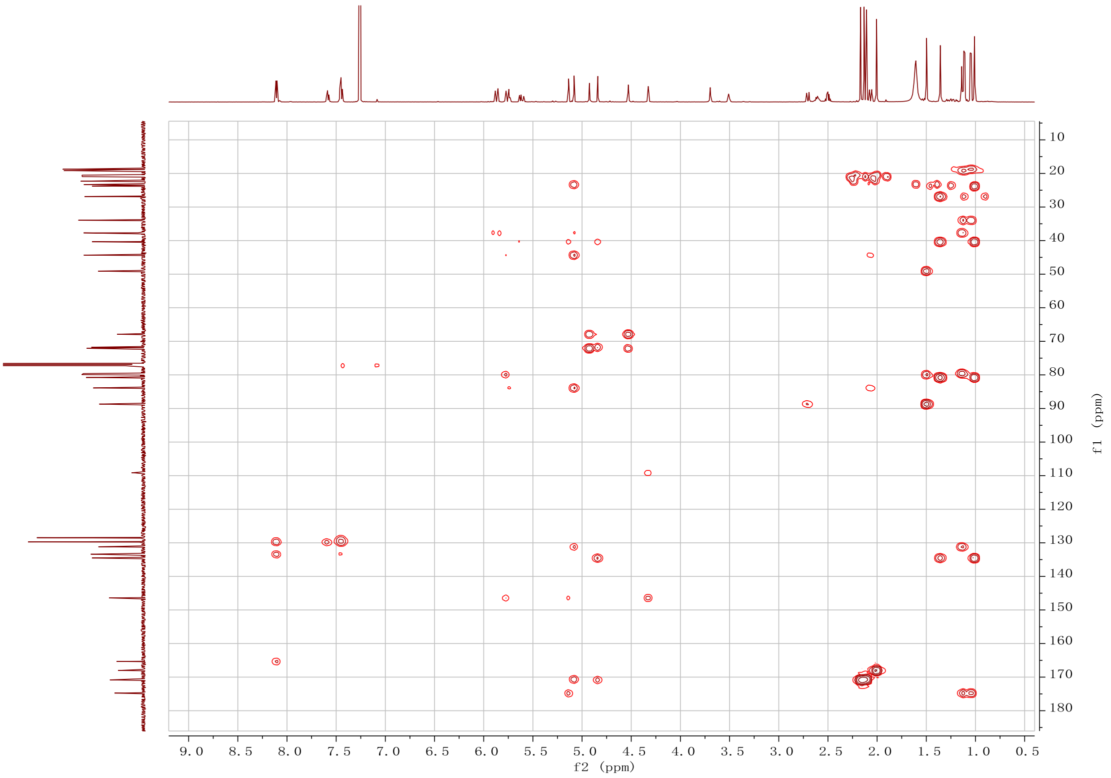

Figure S45. HMBC spectrum of euphjatrophane D (**5**)

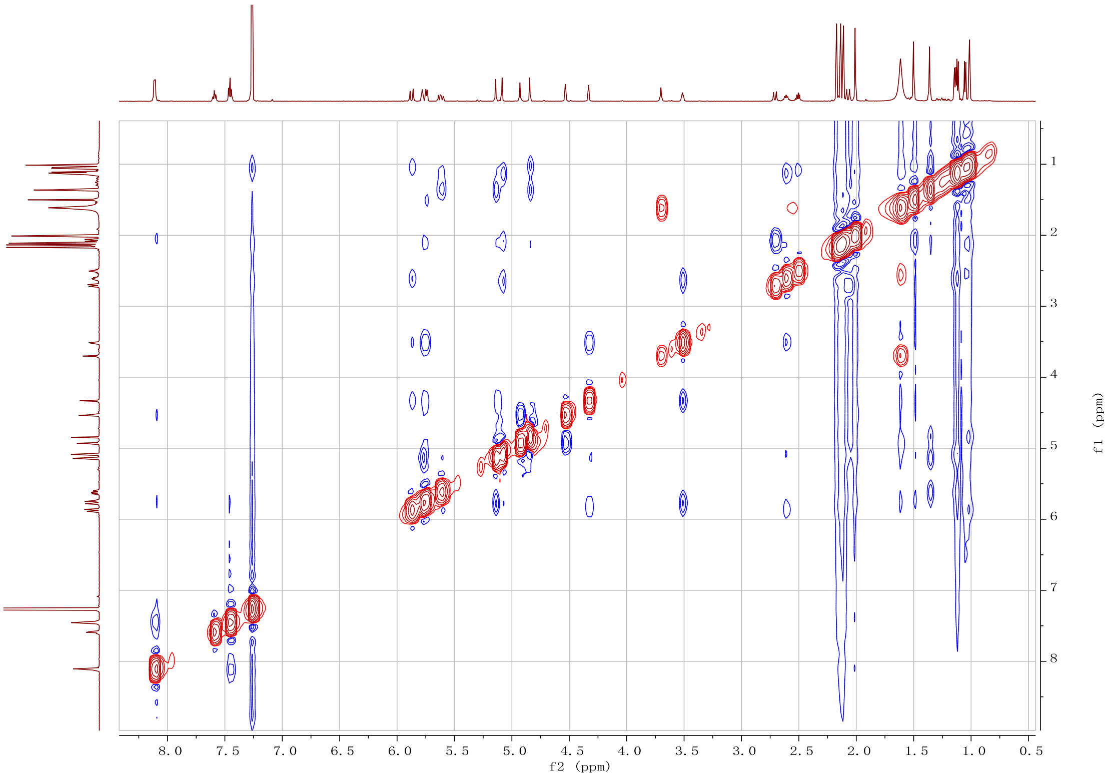

Figure S46. ROESY spectrum of euphjatrophane D (**5**)

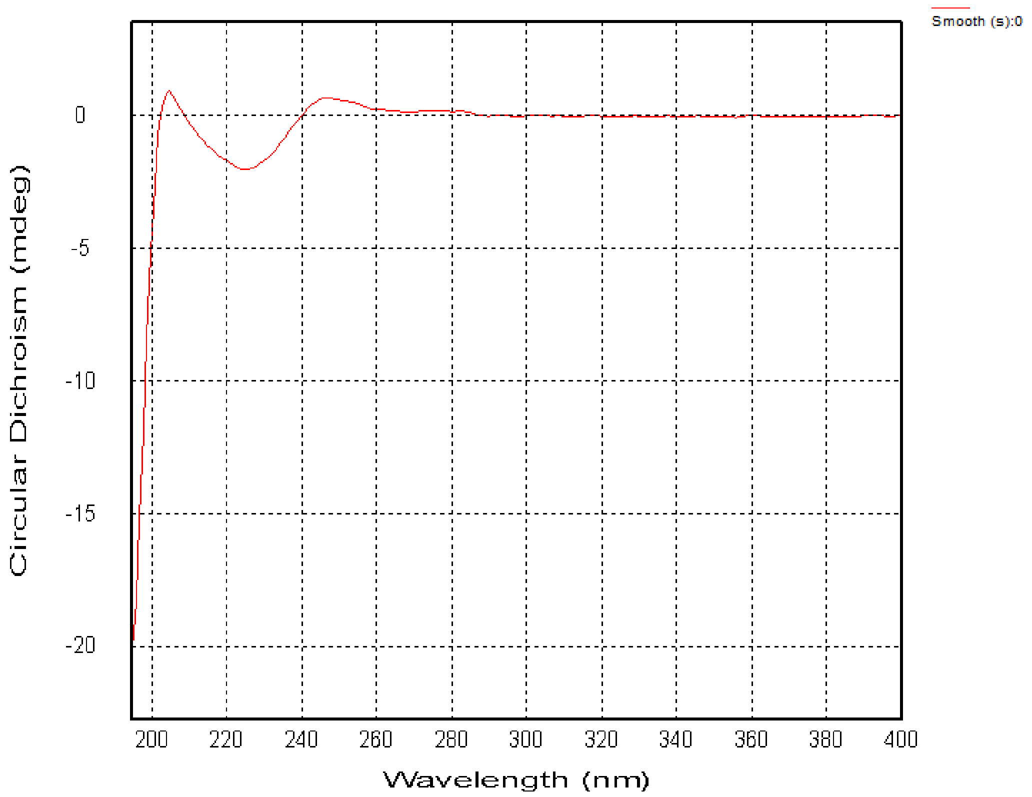

Figure S47. CD spectrum of euphjatrophane D (**5**)

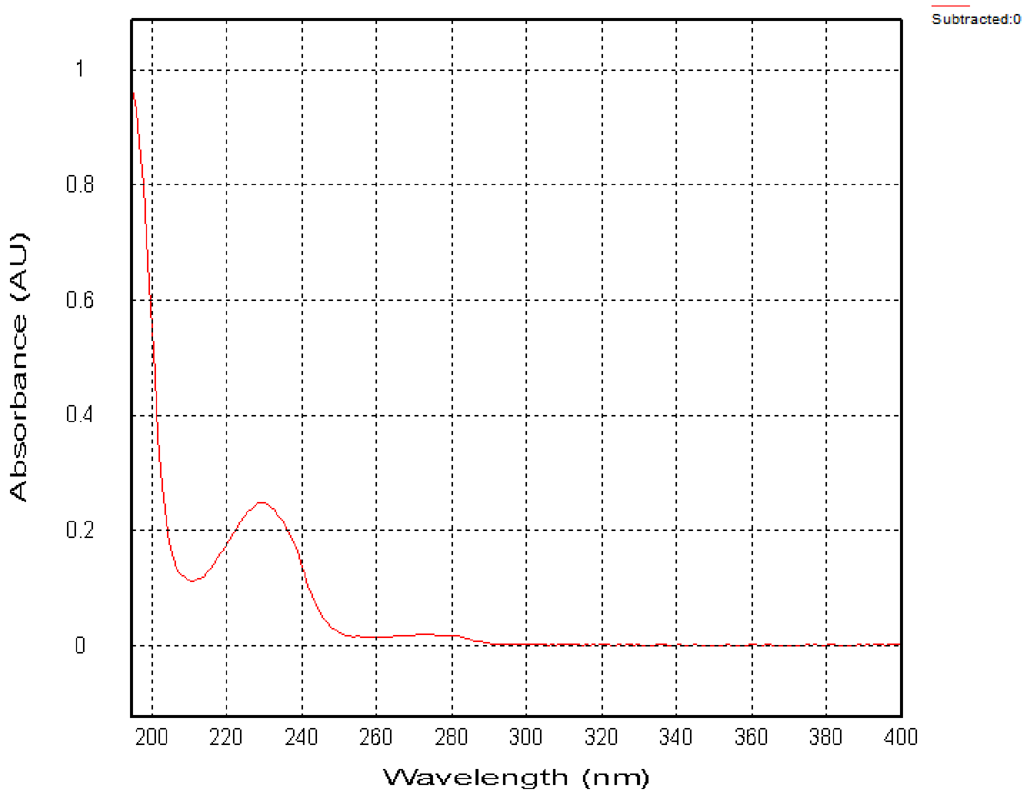

Figure S48. UV spectrum of euphjatrophane D (**5**)

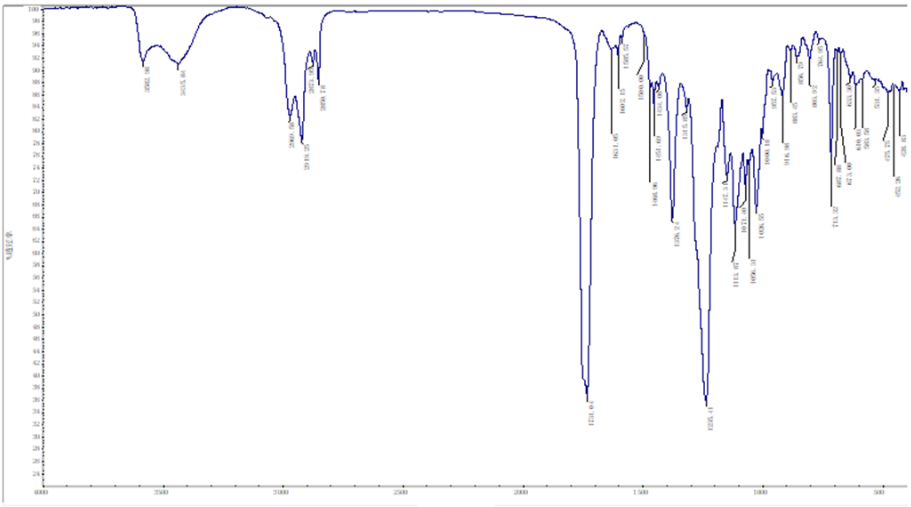

Wavenumber (cm^{-1})

Figure S49. IR spectrum of euphjatrophane D (**5**)

Qualitative Analysis Report

**Data Filename**	HLQY-6212.d	**Sample Name**	HLQY-6212
**Sample Type**	Sample	**Position**	P1-A6
**Instrument Name**	Instrument 1	**User Name**	
**Acq Method**	s.m	**Acquired Time**	8/31/2020 4:03:42 PM
**IRM Calibration Status**	Success	**DA Method**	Default.m
**Comment**			

Sample Group **Info.**
Acquisition SW 6200 series TOF/6500 series
Version Q-TOF B.05.01 (B5125.2)

User Spectra

Fragmentor Voltage	Collision Energy	Ionization Mode
135	0	ESI

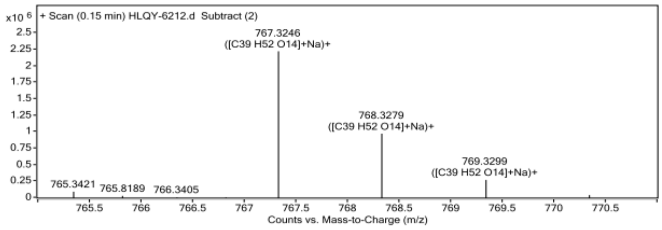

Peak List

m/z	z	Abund	Formula	Ion
727.3323	1	3352046.5		
728.3354	1	1477377.5		
729.338	1	415997.06		
762.3691	1	2333769.5		
763.3727	1	1032560.44		
764.3563	1	383109		
767.3246	1	2223751	C39 H52 O14	(M+Na)+
768.3279	1	974630.06	C39 H52 O14	(M+Na)+
769.3299	1	277421.38	C39 H52 O14	(M+Na)+
783.298	1	359401.5		

Formula Calculator Element Limits

Element	Min	Max
C	3	60
H	0	120
O	0	30

Formula Calculator Results

Formula	CalculatedMass	CalculatedMz	Mz	Diff. (mDa)	Diff. (ppm)	DBE
C39 H52 O14	744.3357	767.3249	767.3246	0.30	0.39	14.0000

--- End Of Report ---

Figure S50. (+)-HRESIMS spectrum of euphjatrophane D (**5**)

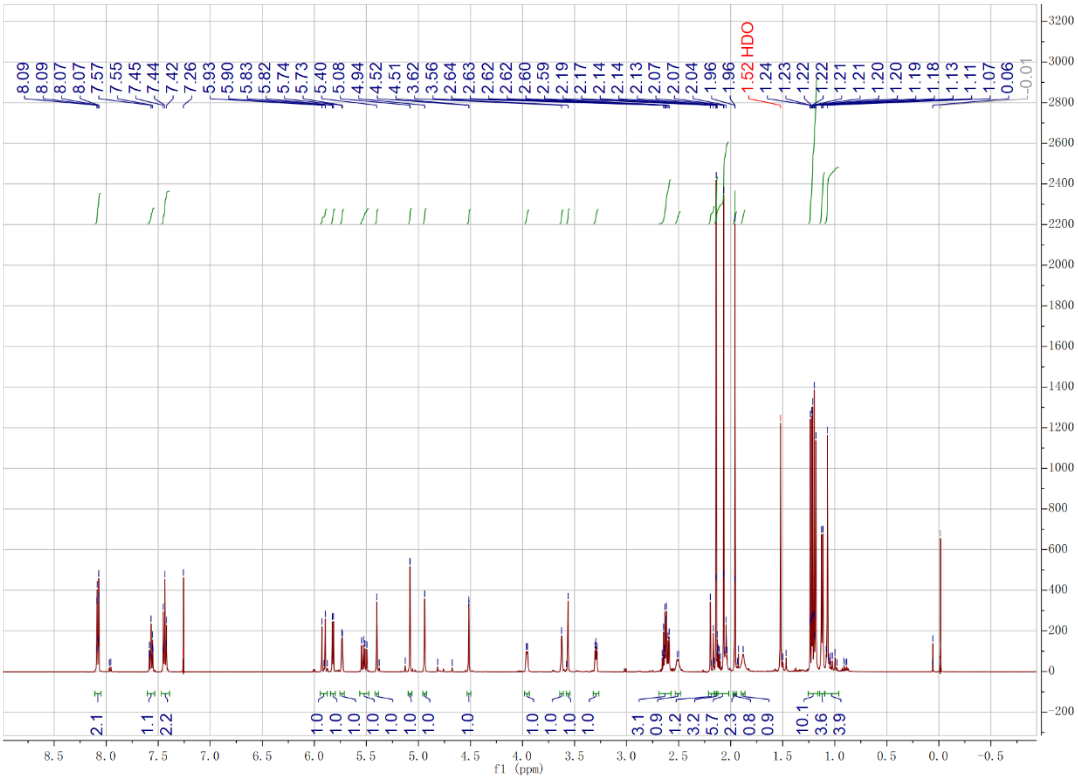

Figure S51. ^{1}H NMR spectrum of euphjatrophane E (**6**)

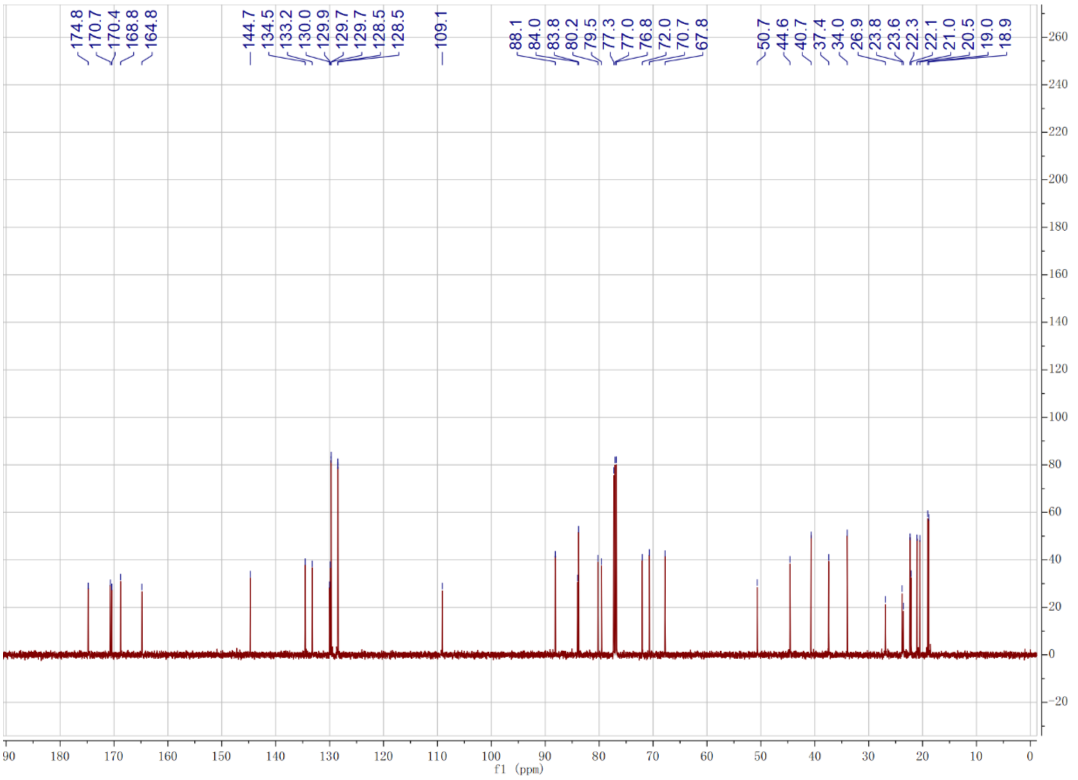

Figure S52. ^{13}C NMR spectrum of euphjatrophane E (**6**)

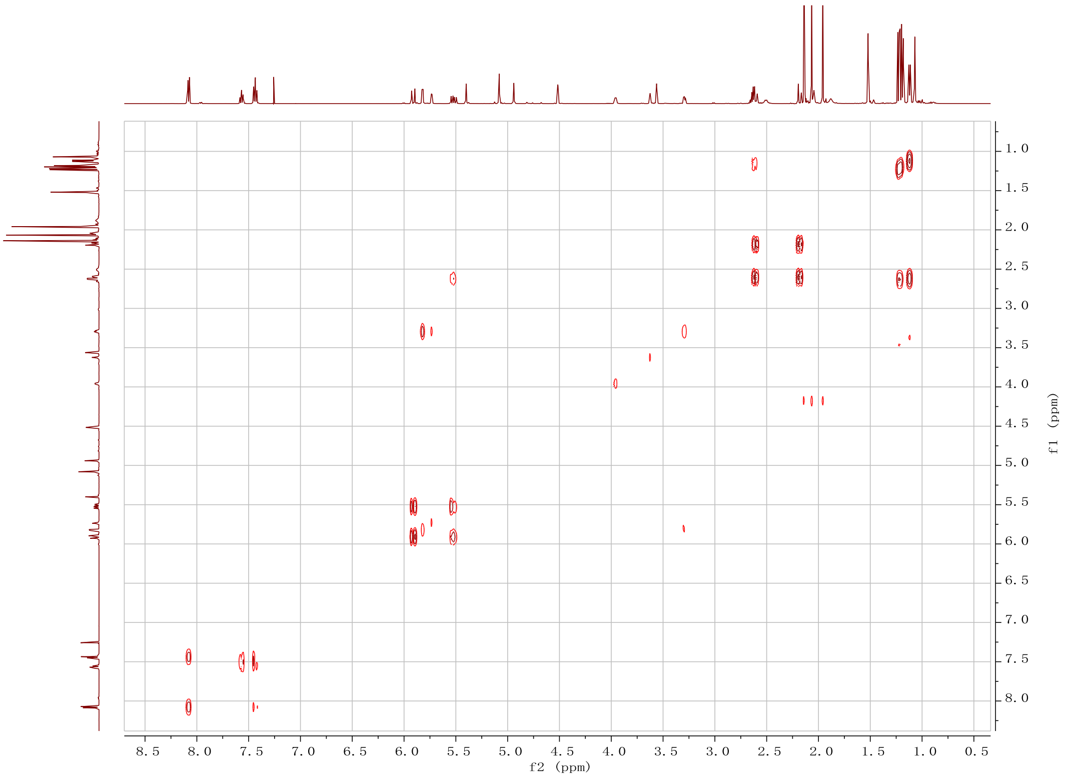

Figure S53. ^{1}H-^{1}H COSY spectrum of euphjatrophane E (**6**)

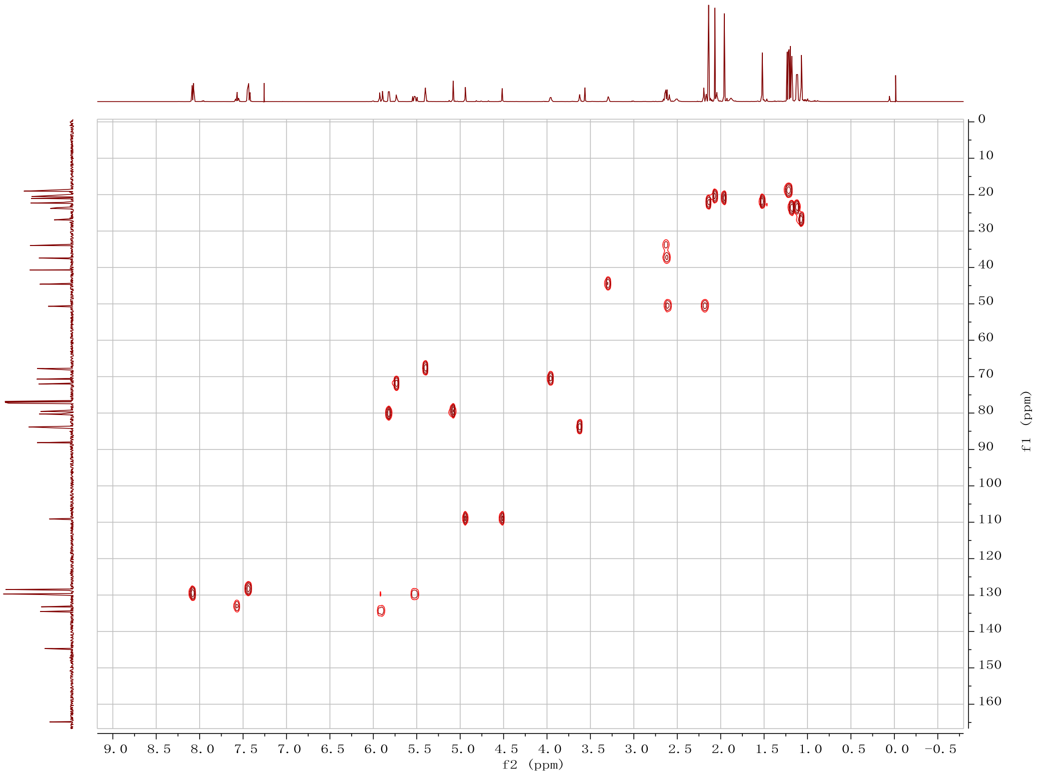

Figure S54. HSQC spectrum of euphjatrophane E (**6**)

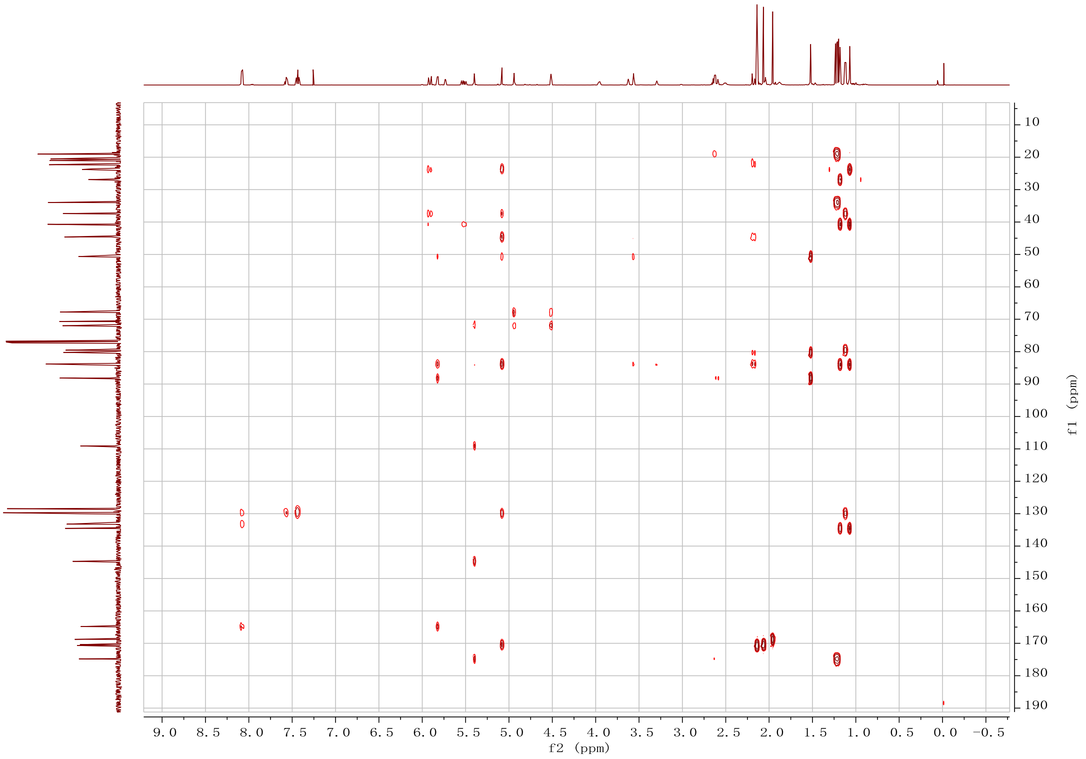

Figure S55. HMBC spectrum of euphjatrophane E (**6**)

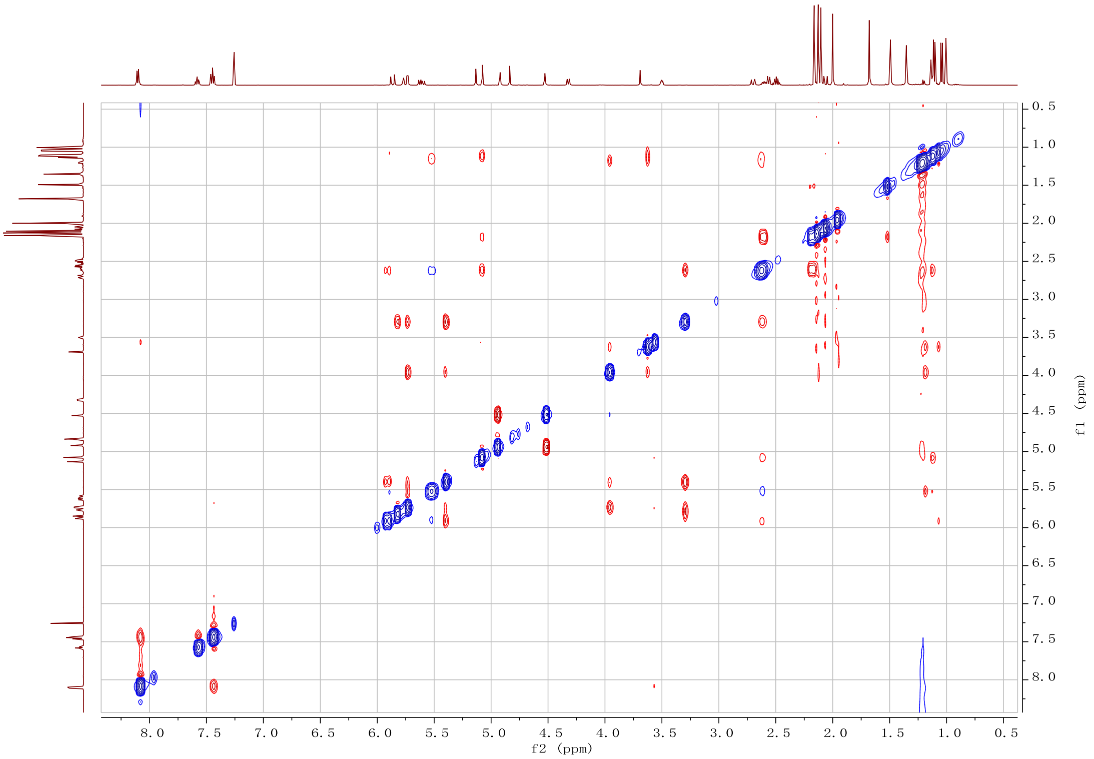

Figure S56. ROESY spectrum of euphjatrophane E (**6**)

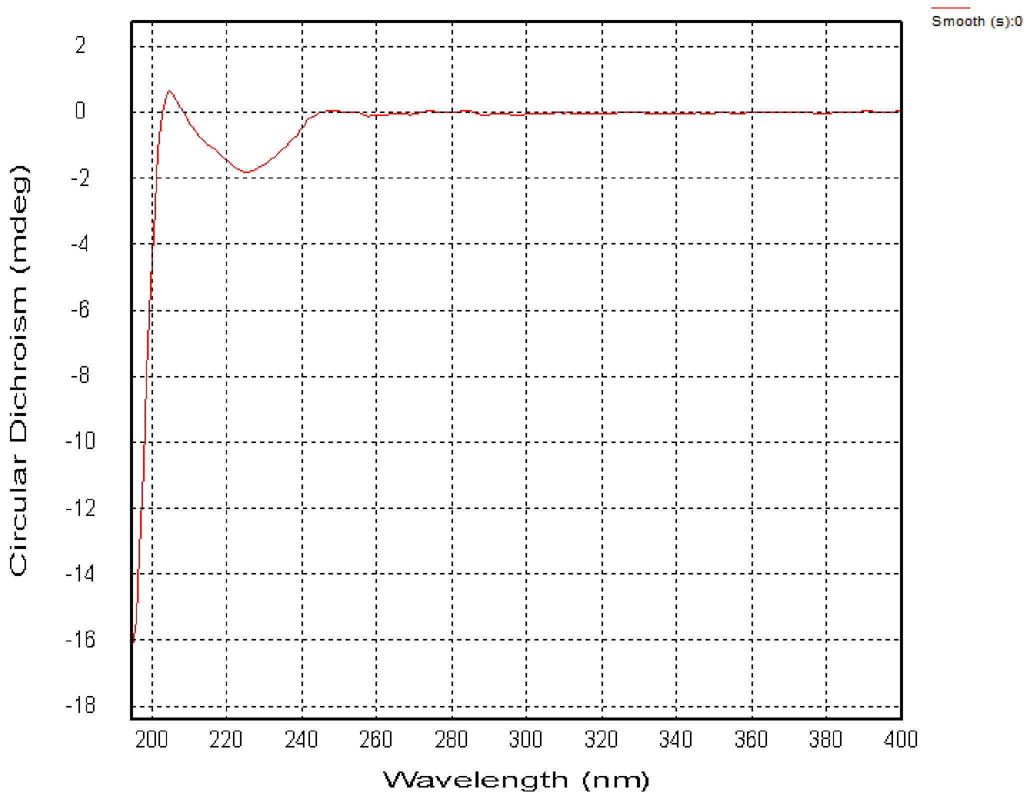

Figure S57. CD spectrum of euphjatrophane E (**6**)

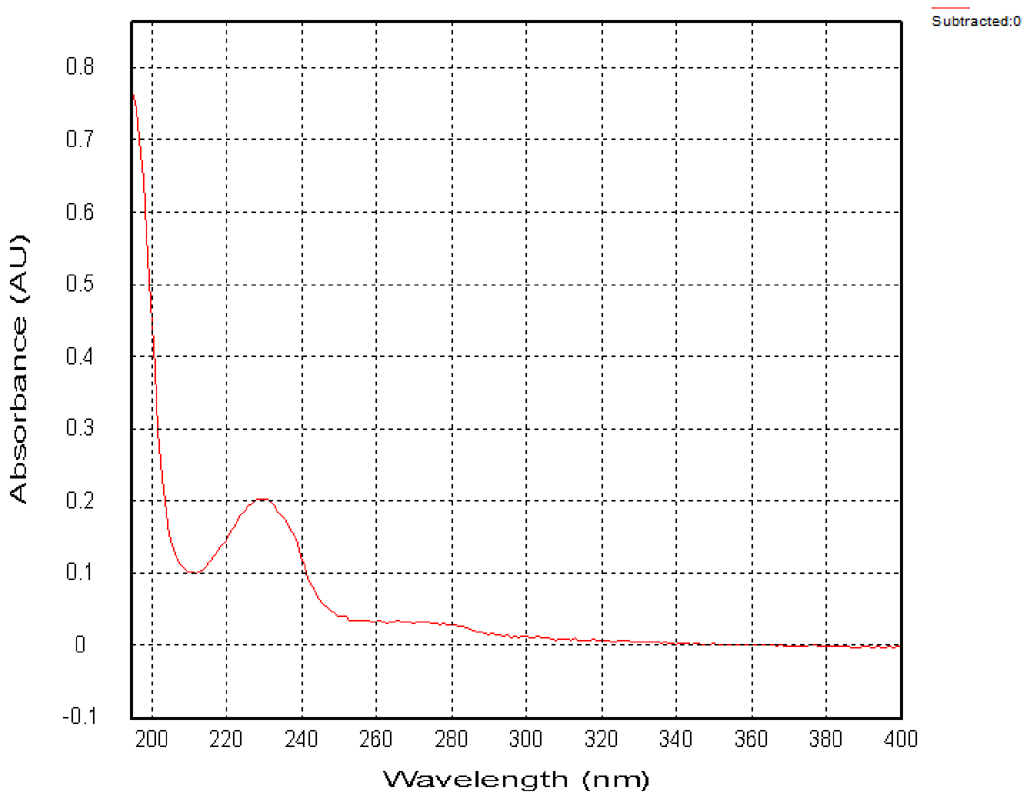

Figure S58. UV spectrum of euphjatrophane E (**6**)

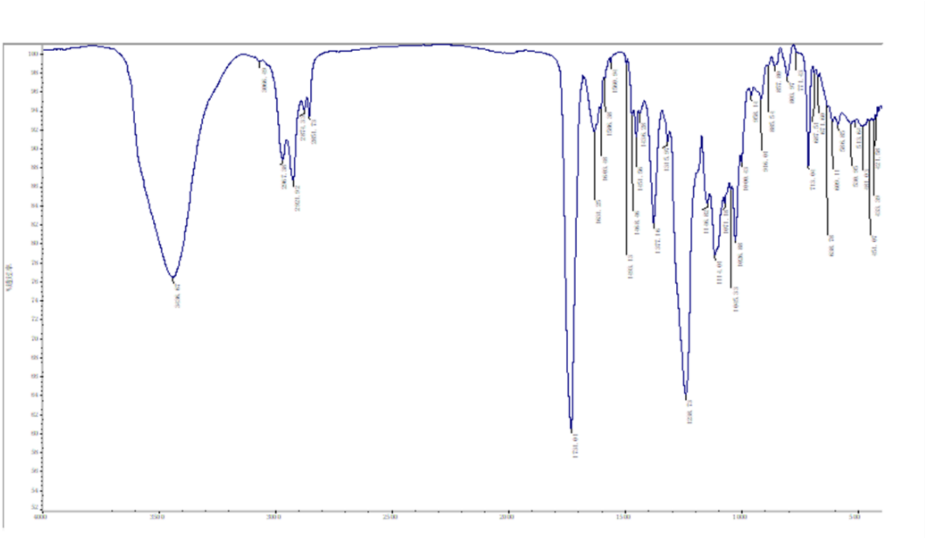

Figure S59. IR spectrum of euphjatrophane E (**6**)

Qualitative Analysis Report

**Data Filename**	HLQY-212.d	**Sample Name**	HLQY-212
**Sample Type**	Sample	**Position**	P1-C2
**Instrument Name**	Instrument 1	**User Name**	
**Acq Method**	s.m	**Acquired Time**	7/13/2020 3:08:00 PM
**IRM Calibration Status**	Success	**DA Method**	Default.m
**Comment**			

Sample Group **Info.**
Acquisition SW 6200 series TOF/6500 series
Version Q-TOF B.05.01 (B5125.2)

User Spectra

Fragmentor Voltage	Collision Energy	Ionization Mode
135	0	ESI

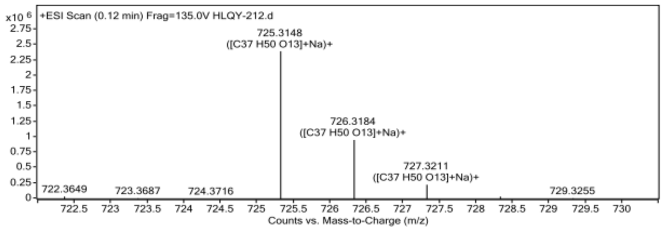

Peak List

m/z	z	Abund	Formula	Ion
720.3595	1	397637.34		
721.3624	1	163782.09		
725.3148	1	2393288.75	C37 H50 O13	(M+Na)+
726.3184	1	955570.06	C37 H50 O13	(M+Na)+
727.3211	1	228703.84	C37 H50 O13	(M+Na)+
741.2887	1	140841.22		
748.3908	1	260615.67		
1427.6409	1	373467.72		
1428.6441	1	299112.44		
1429.6466	1	131236.53		

Formula Calculator Element Limits

Element	Min	Max
C	3	60
H	0	120
O	0	30

Formula Calculator Results

Formula	CalculatedMass	CalculatedMz	Mz	Diff. (mDa)	Diff. (ppm)	DBE
C37 H50 O13	702.3251	725.3144	725.3148	-0.40	-0.55	13.0000

--- End Of Report ---

Figure S60. (+)-HRESIMS spectrum of euphjatrophane E (**6**)

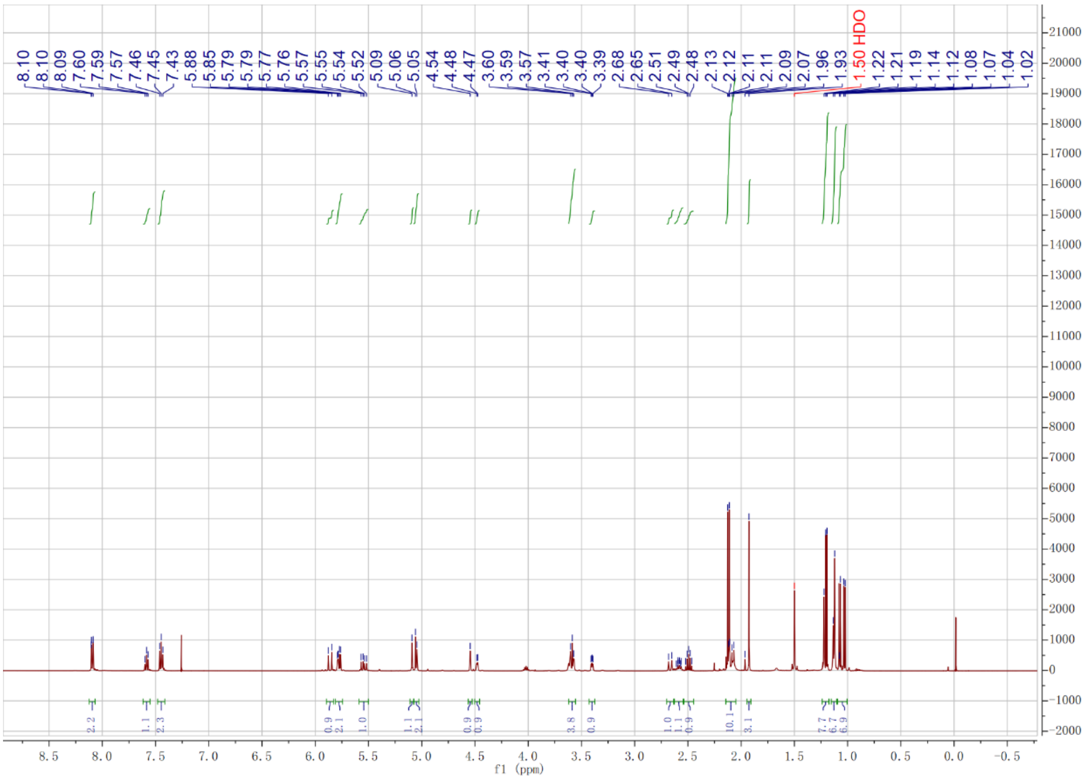

Figure S61. ^{1}H NMR spectrum of euphjatrophane F (**7**)

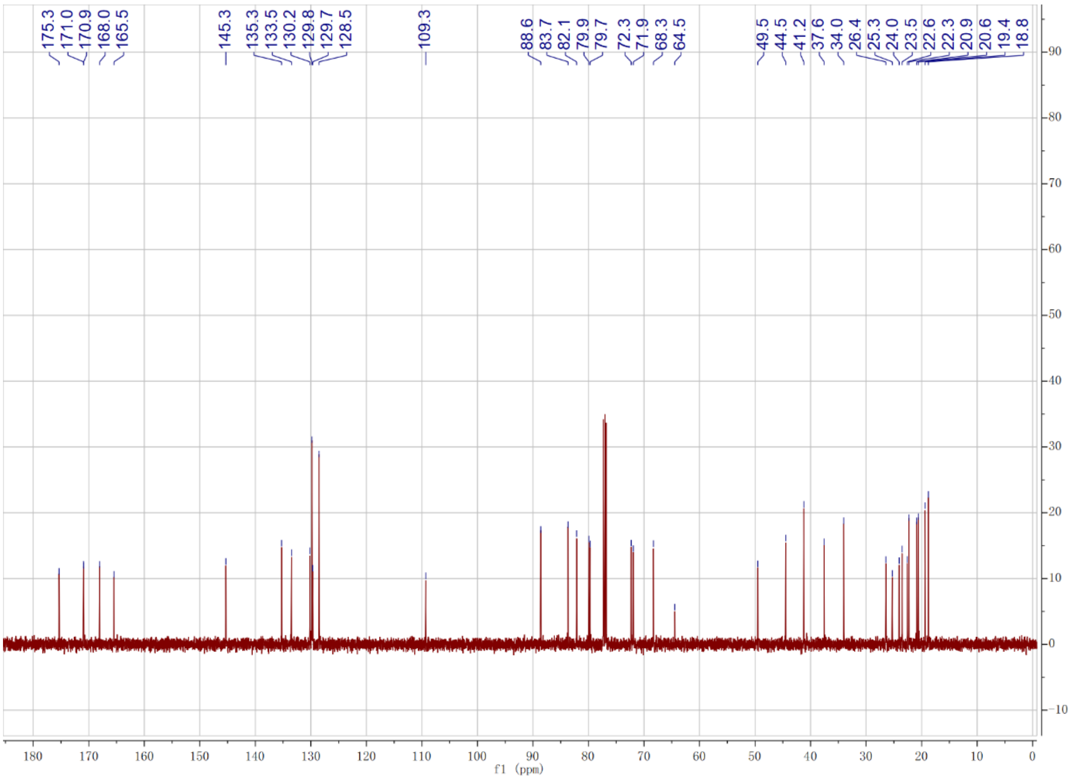

Figure S62. ^{13}C NMR spectrum of euphjatrophane F (**7**)

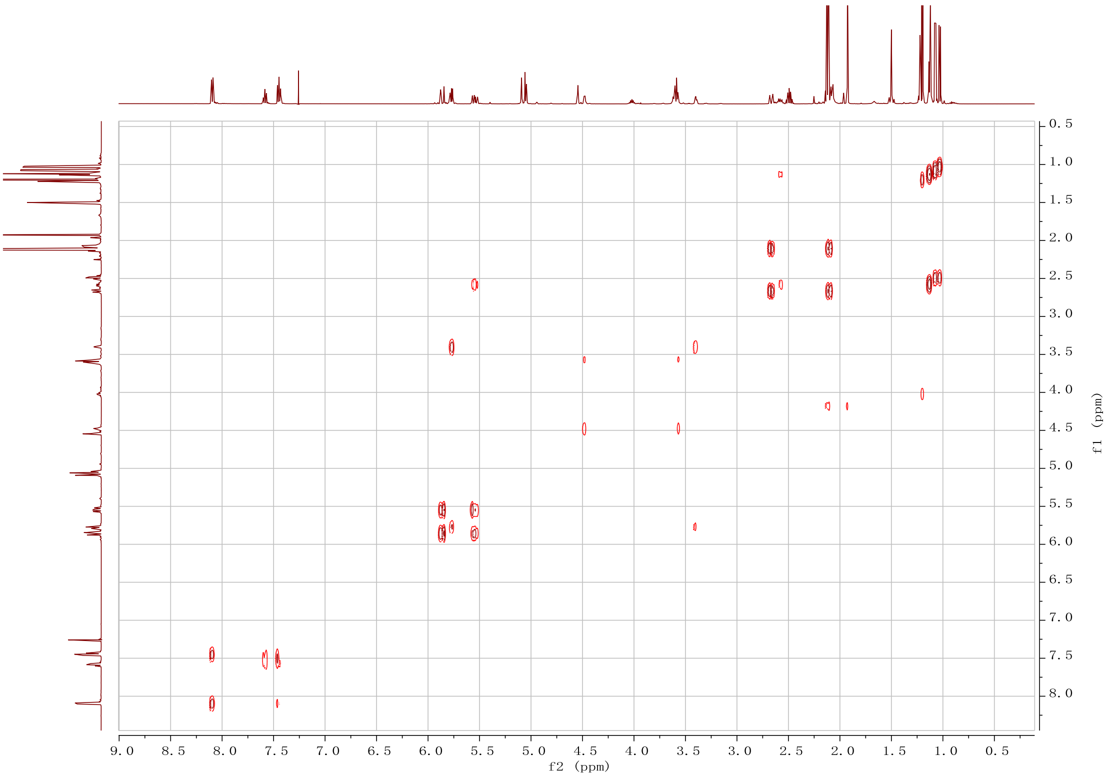

Figure S63. ^{1}H-^{1}H COSY spectrum of euphjatrophane F (**7**)

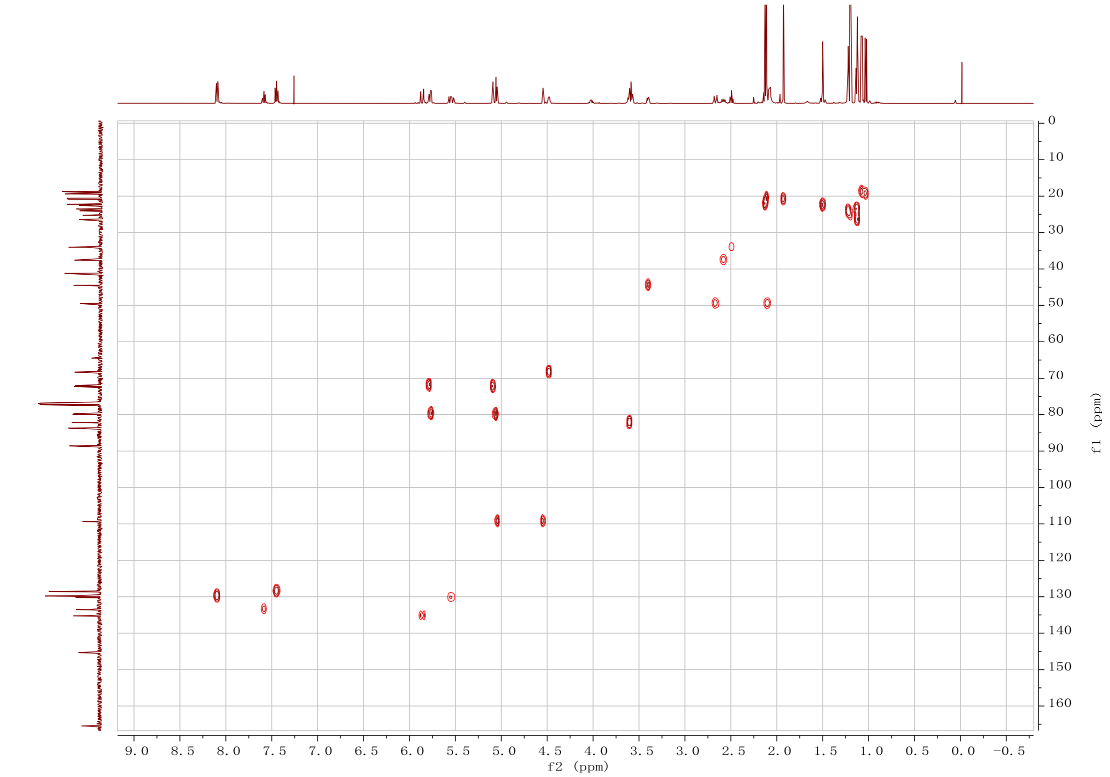

Figure S64. HSQC spectrum of euphjatrophane F (**7**)

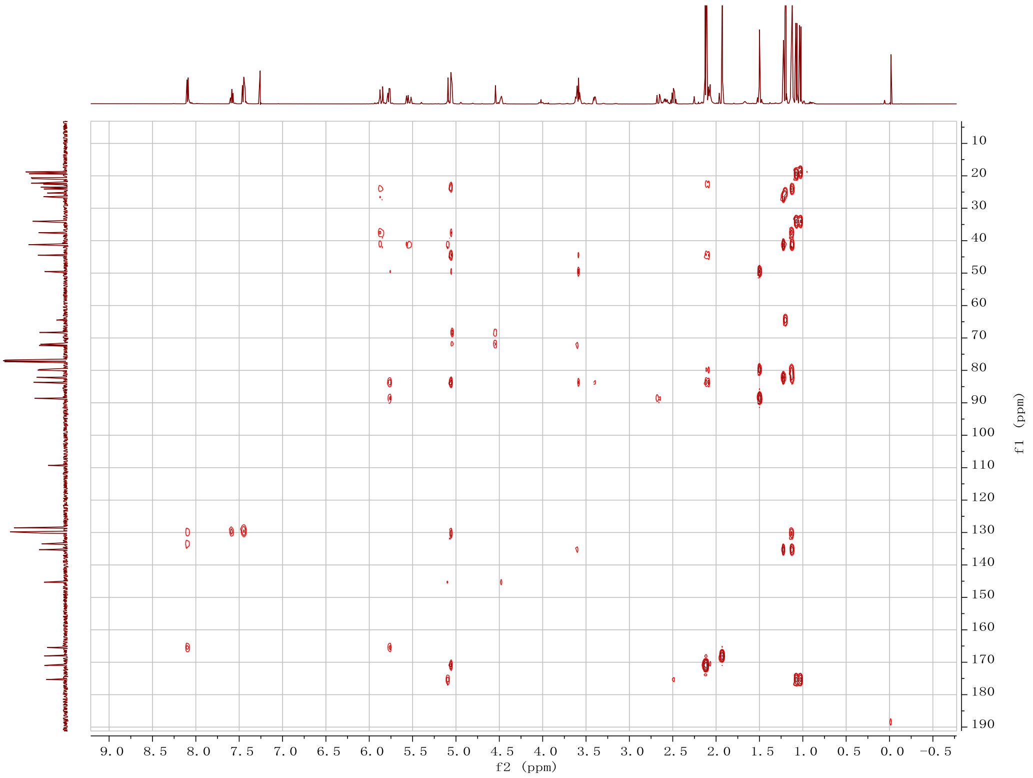

Figure S65. HMBC spectrum of euphjatrophane F (**7**)

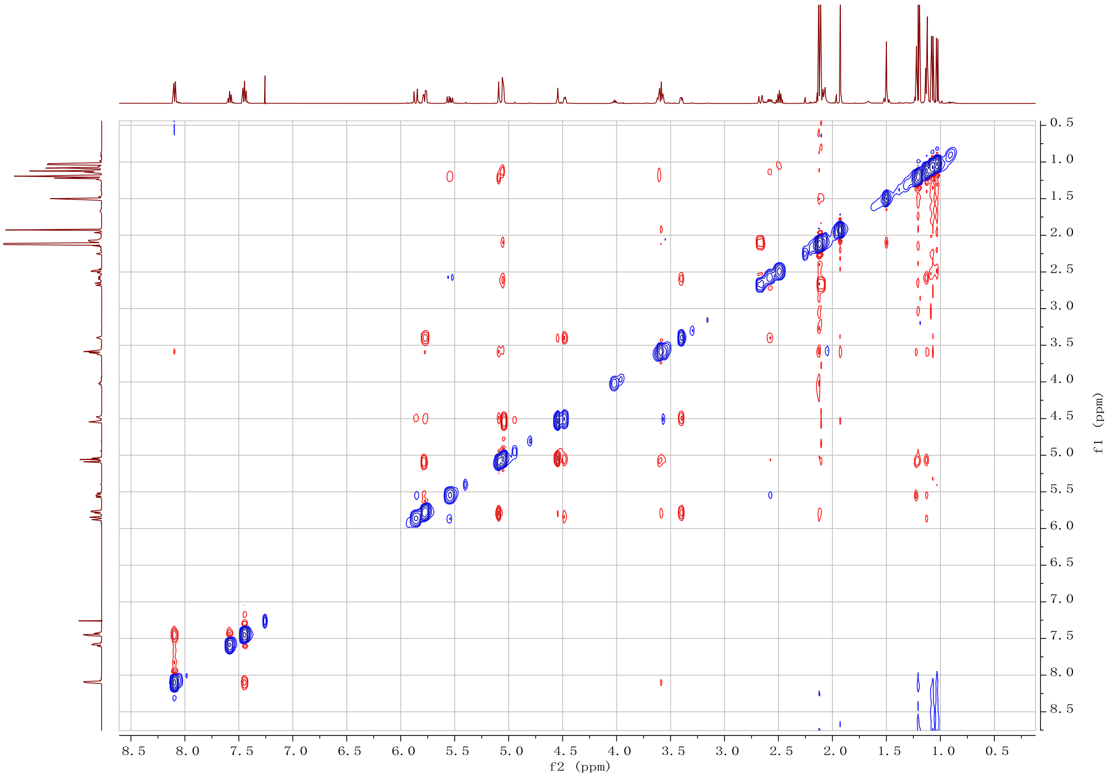

Figure S66. ROESY spectrum of euphjatrophane F (**7**)

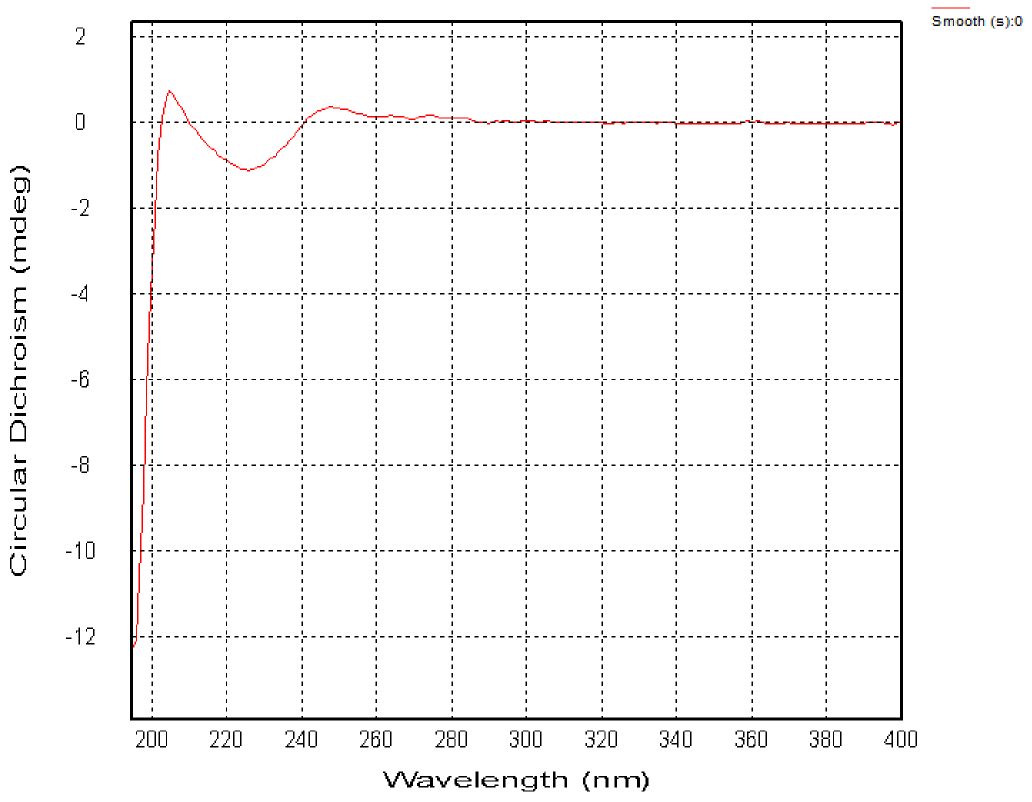

Figure S67. CD spectrum of euphjatrophane F (**7**)

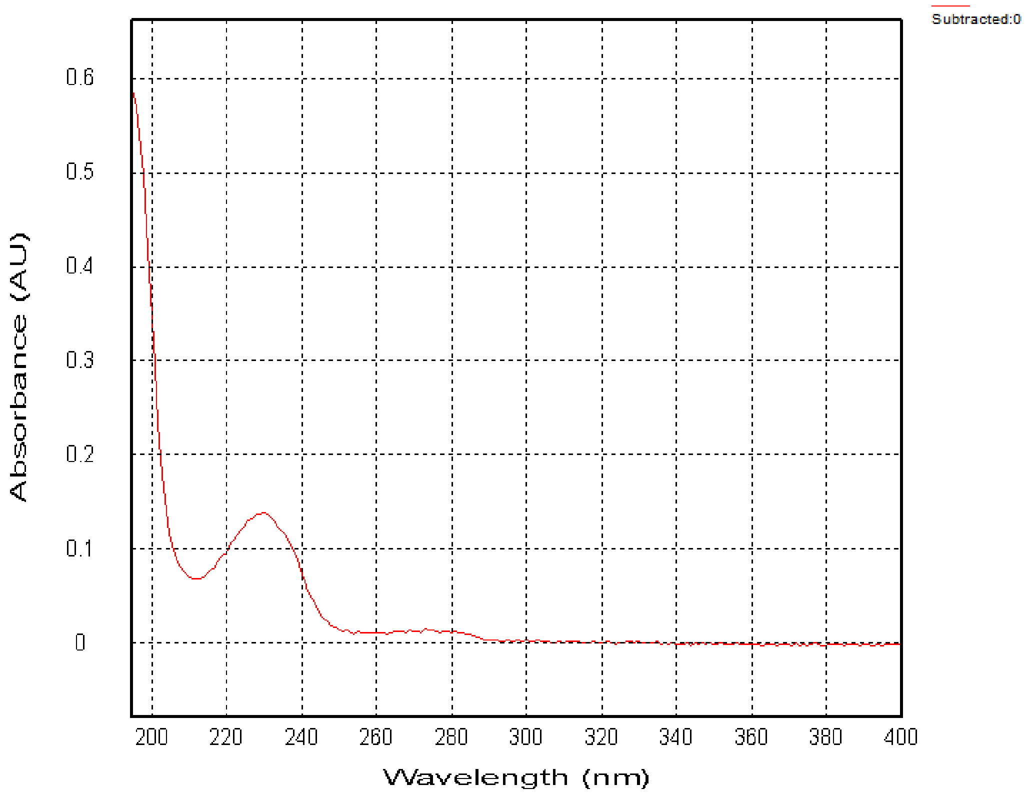

Figure S68. UV spectrum of euphjatrophane F (**7**)

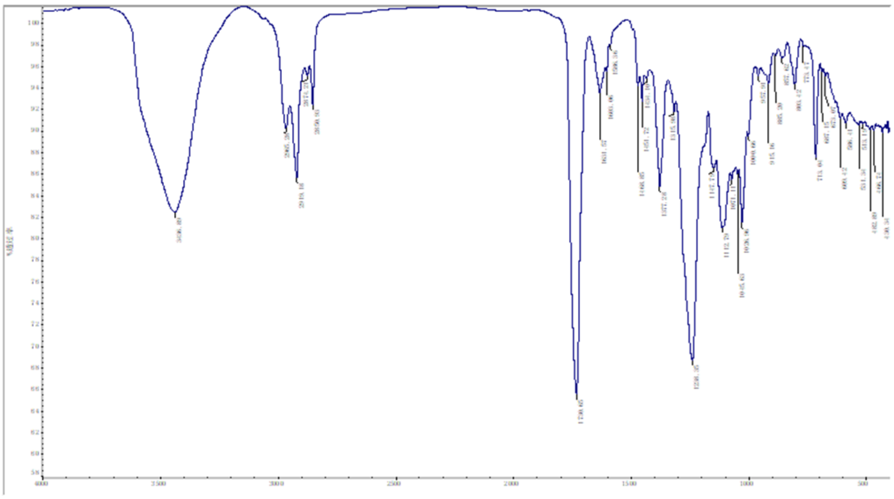

Wavenumber (cm^{-1})

Figure S69. IR spectrum of euphjatrophane F (**7**)

Qualitative Analysis Report

**Data Filename**	HLQY-211.d	**Sample Name**	HLQY-211
**Sample Type**	Sample	**Position**	P1-C1
**Instrument Name**	Instrument 1	**User Name**	
**Acq Method**	s.m	**Acquired Time**	7/13/2020 3:06:49 PM
**IRM Calibration Status**	Success	**DA Method**	Default.m
**Comment**			

Sample Group **Info.**

Acquisition SW 6200 series TOF/6500 series

Version Q-TOF B.05.01 (B5125.2)

User Spectra

Fragmentor Voltage	Collision Energy	Ionization Mode
135	0	ESI

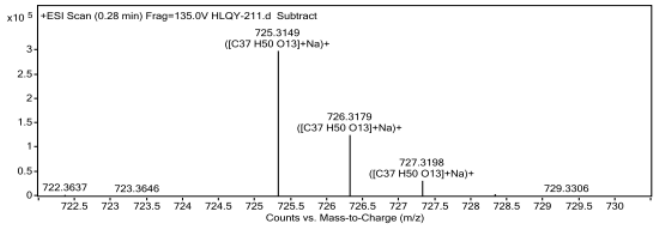

Peak List

m/z	z	Abund	Formula	Ion
683.3043	1	12952.36		
720.3589	1	28360.97		
721.3622	1	12745.93		
725.3149	1	298945.53	C37 H50 O13	(M+Na)+
726.3179	1	125976.79	C37 H50 O13	(M+Na)+
727.3198	1	31686.18	C37 H50 O13	(M+Na)+
741.2883	1	35291.52		
742.292	1	12583.82		
748.3903	1	45541.17		
749.3931	1	18295.9		

Formula Calculator Element Limits

Element	Min	Max
C	3	60
H	0	120
O	0	30

Formula Calculator Results

Formula	CalculatedMass	CalculatedMz	Mz	Diff. (mDa)	Diff. (ppm)	DBE
C37 H50 O13	702.3251	725.3144	725.3149	-0.50	-0.69	13.0000

--- End Of Report ---

Figure S70. (+)-HRESIMS spectrum of euphjatrophane F (**7**)

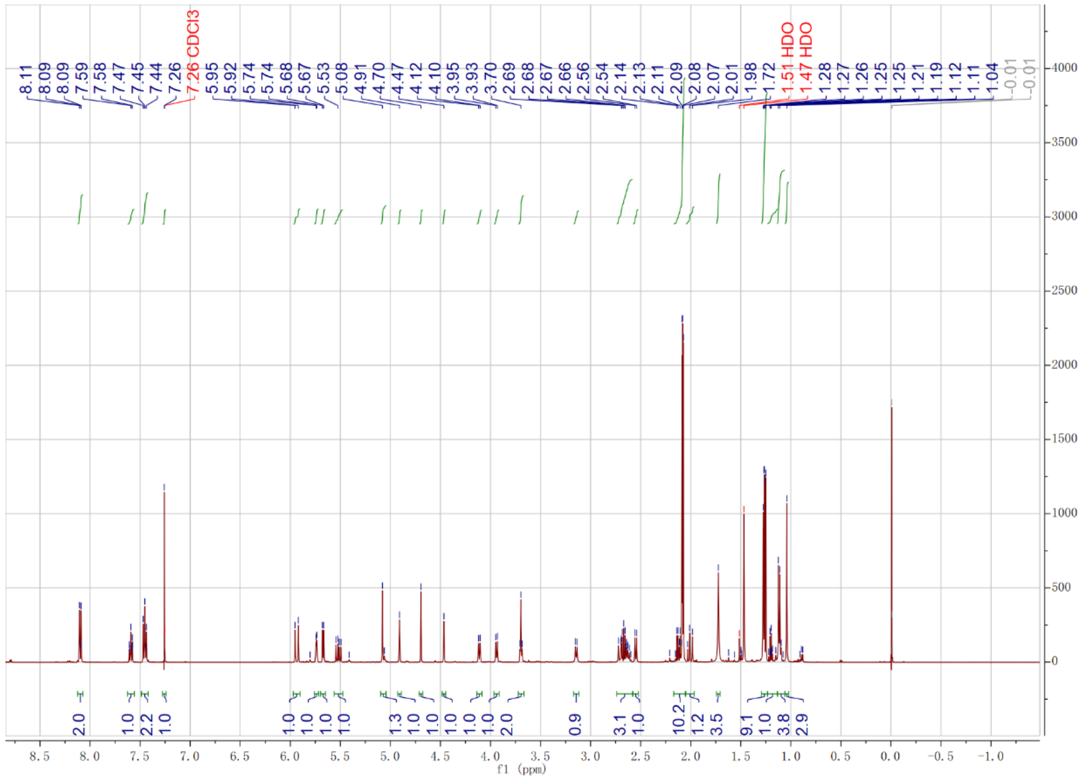

Figure S71. ^{1}H NMR spectrum of euphjatrophane G (**8**)

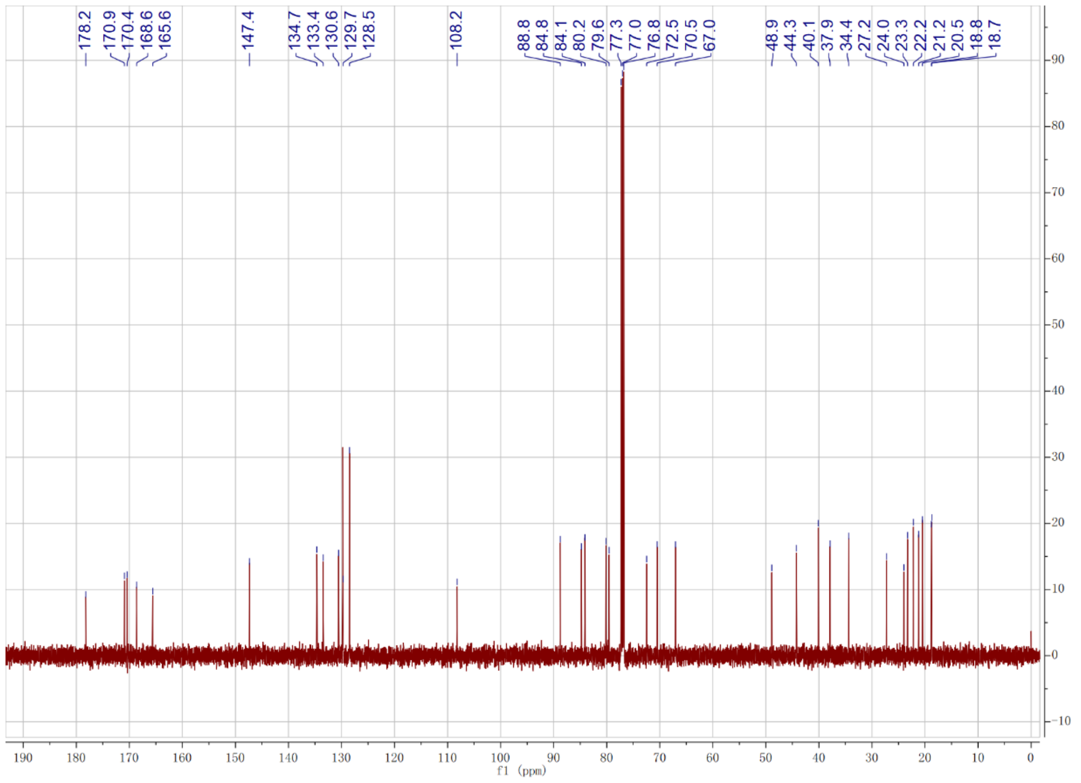

Figure S72. ^{13}C NMR spectrum of euphjatrophane G (**8**)

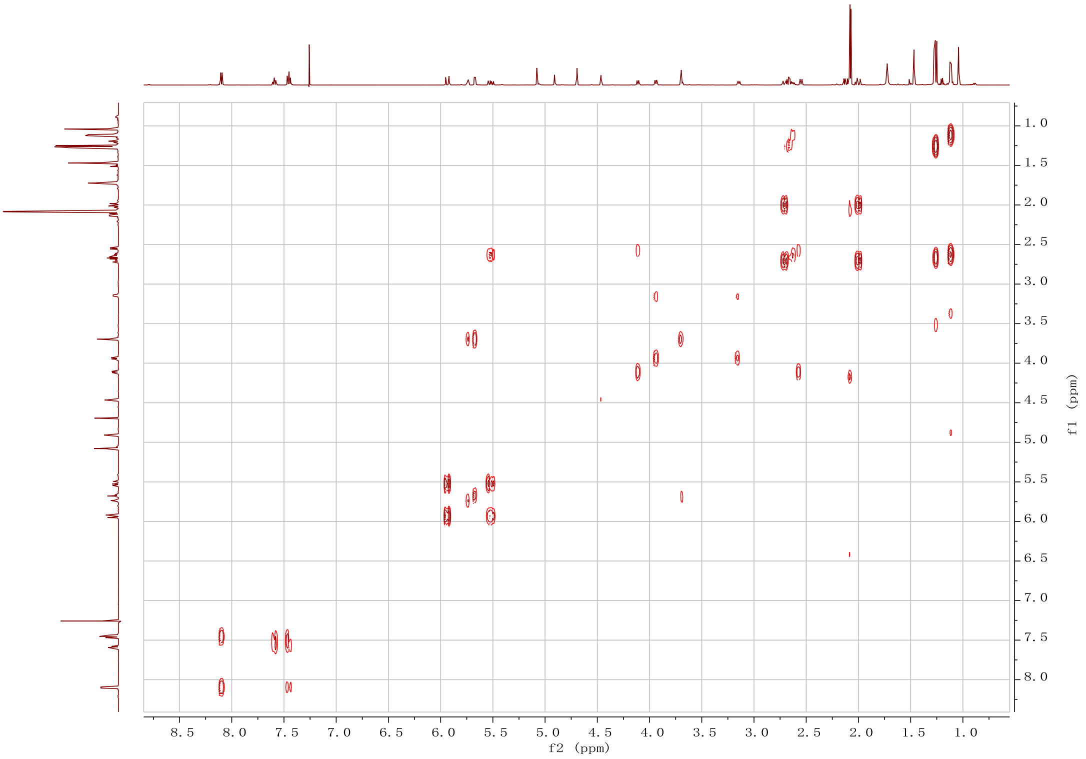

Figure S73. ^{1}H-^{1}H COSY spectrum of euphjatrophane G (**8**)

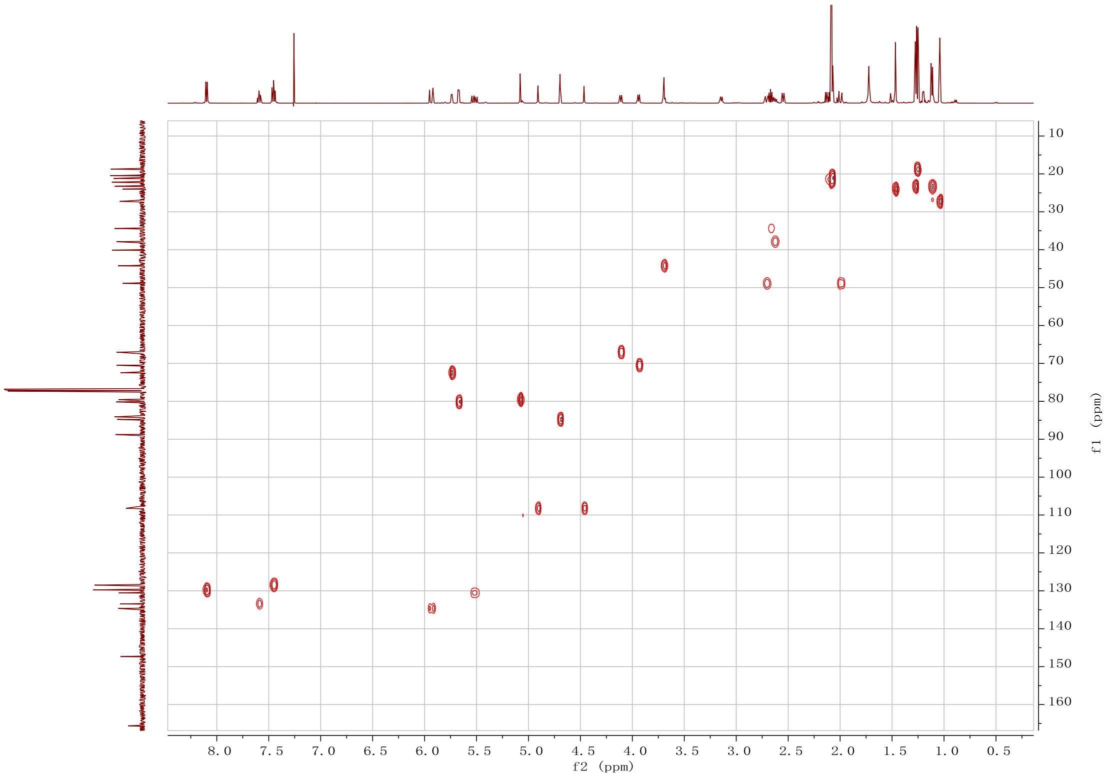

Figure S74. HSQC spectrum of euphjatrophane G (**8**)

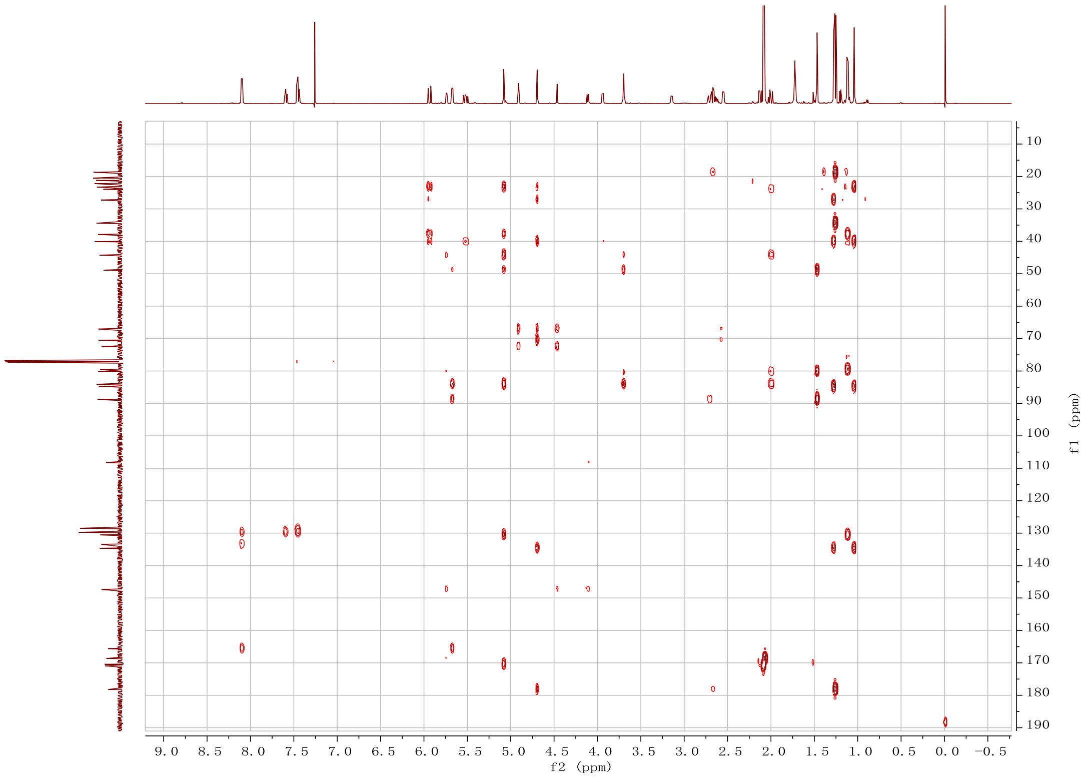

Figure S75. HMBC spectrum of euphjatrophane G (**8**)

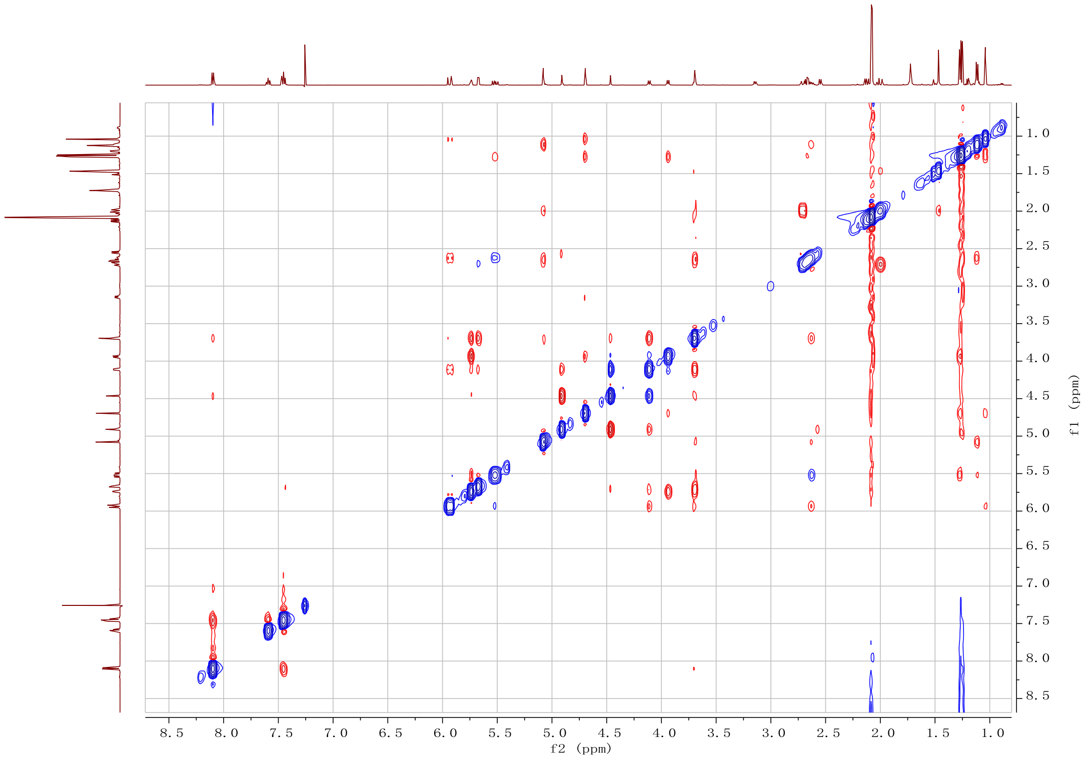

Figure S76. ROESY spectrum of euphjatrophane G (**8**)

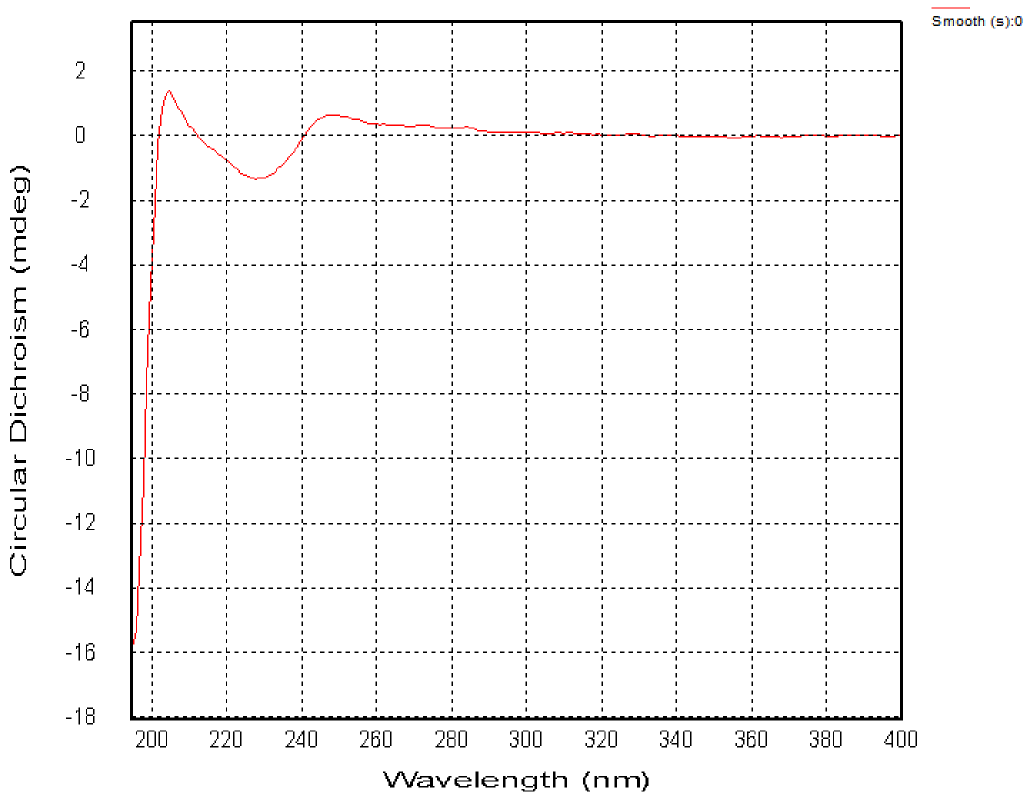

Figure S77. CD spectrum of euphjatrophane G (**8**)

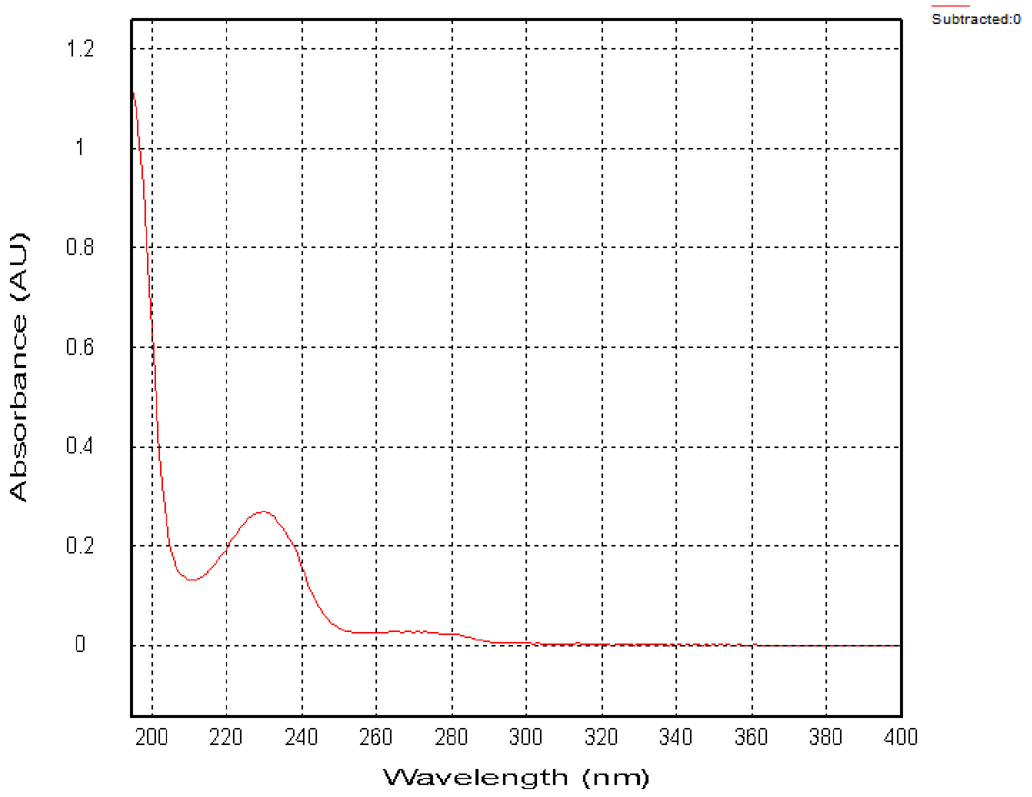

Figure S78. UV spectrum of euphjatrophane G (**8**)

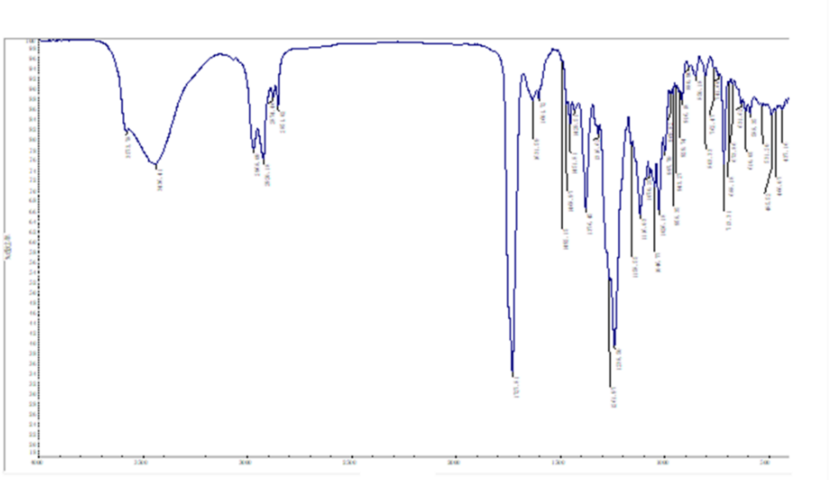

Wavenumber (cm^{-1})

Figure S79. IR spectrum of euphjatrophane G (**8**)

Qualitative Analysis Report

**Data Filename**	HLQY-823.d	**Sample Name**	HLQY-823
**Sample Type**	Sample	**Position**	P1-C3
**Instrument Name**	Instrument 1	**User Name**	
**Acq Method**	s.m	**Acquired Time**	7/13/2020 3:09:11 PM
**IRM Calibration Status**	Success	**DA Method**	Default.m
**Comment**			

**Sample Group**		**Info.**
**Acquisition SW**	6200 series TOF/6500 series	
**Version**	Q-TOF B.05.01 (B5125.2)	

User Spectra

Fragmentor Voltage	Collision Energy	Ionization Mode
135	0	ESI

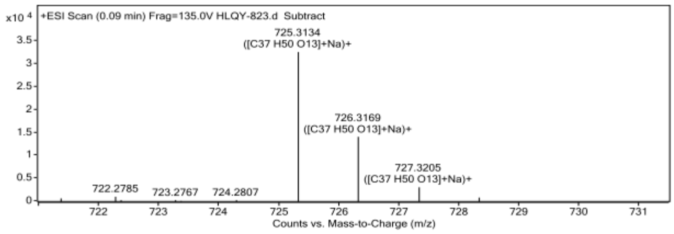

Peak List

m/z	z	Abund	Formula	Ion
245.0779		1740.77		
642.2923	1	2982.86		
664.2722	1	2461.84		
725.3134	1	32670.45	C37 H50 O13	(M+Na)+
726.3169	1	14188.44	C37 H50 O13	(M+Na)+
727.3205	1	3263.09	C37 H50 O13	(M+Na)+
741.2867	1	2554.61		
748.3882	1	2873.74		
749.3927	1	1668.98		
786.307	1	1752.37		

Formula Calculator Element Limits

Element	Min	Max
C	3	60
H	0	120
O	0	30

Formula Calculator Results

Formula	CalculatedMass	CalculatedMz	Mz	Diff. (mDa)	Diff. (ppm)	DBE
C37 H50 O13	702.3251	725.3144	725.3134	1.00	1.38	13.0000

--- End Of Report ---

Figure S80. (+)-HRESIMS spectrum of euphjatrophane G (**8**)

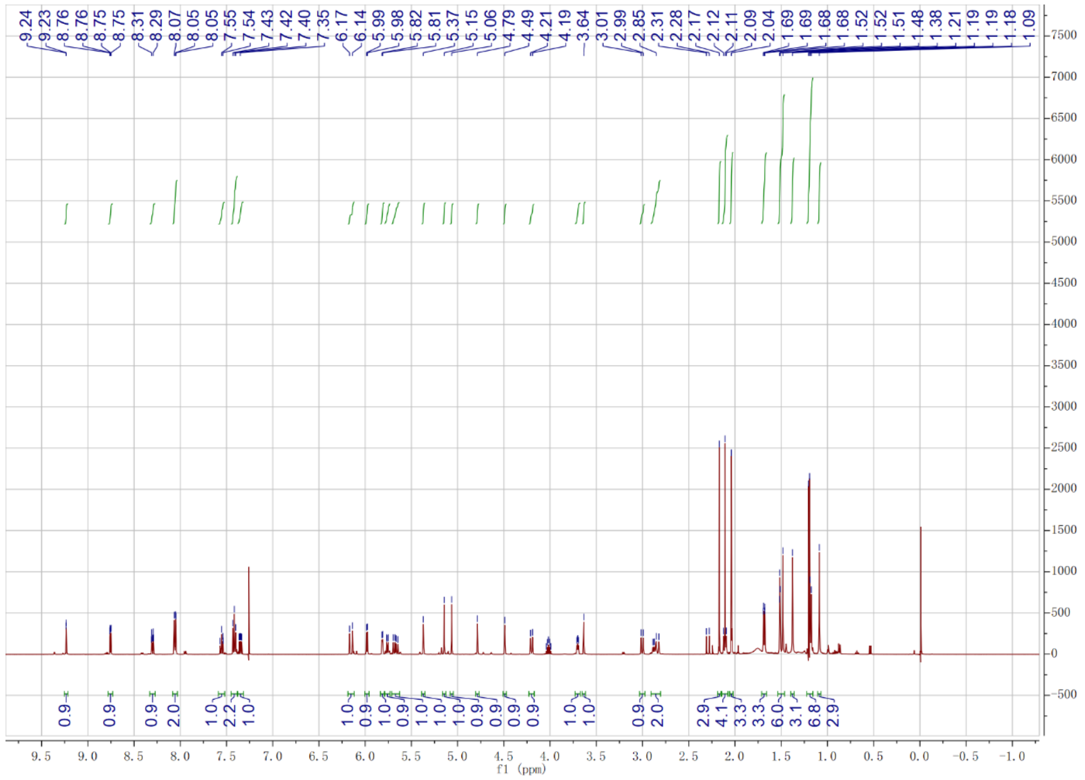

Figure S81. ^{1}H NMR spectrum of euphpepluone K (**9**)

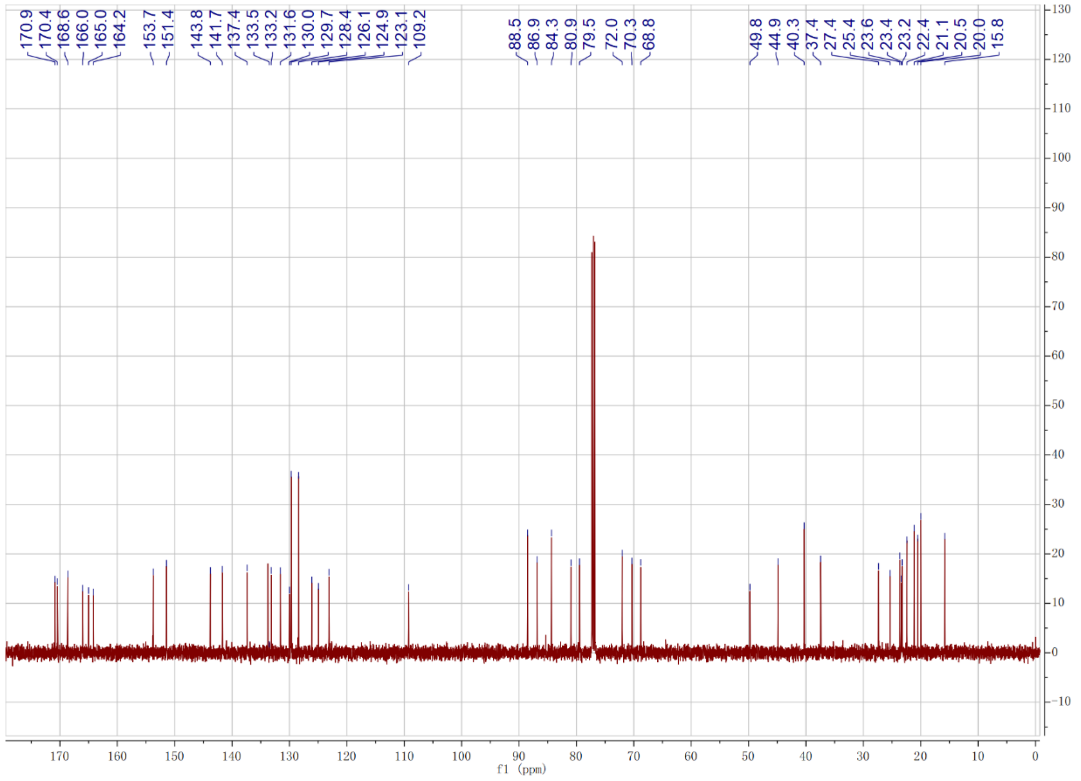

Figure S82. ^{13}C NMR spectrum of euphpepluone K (**9**)

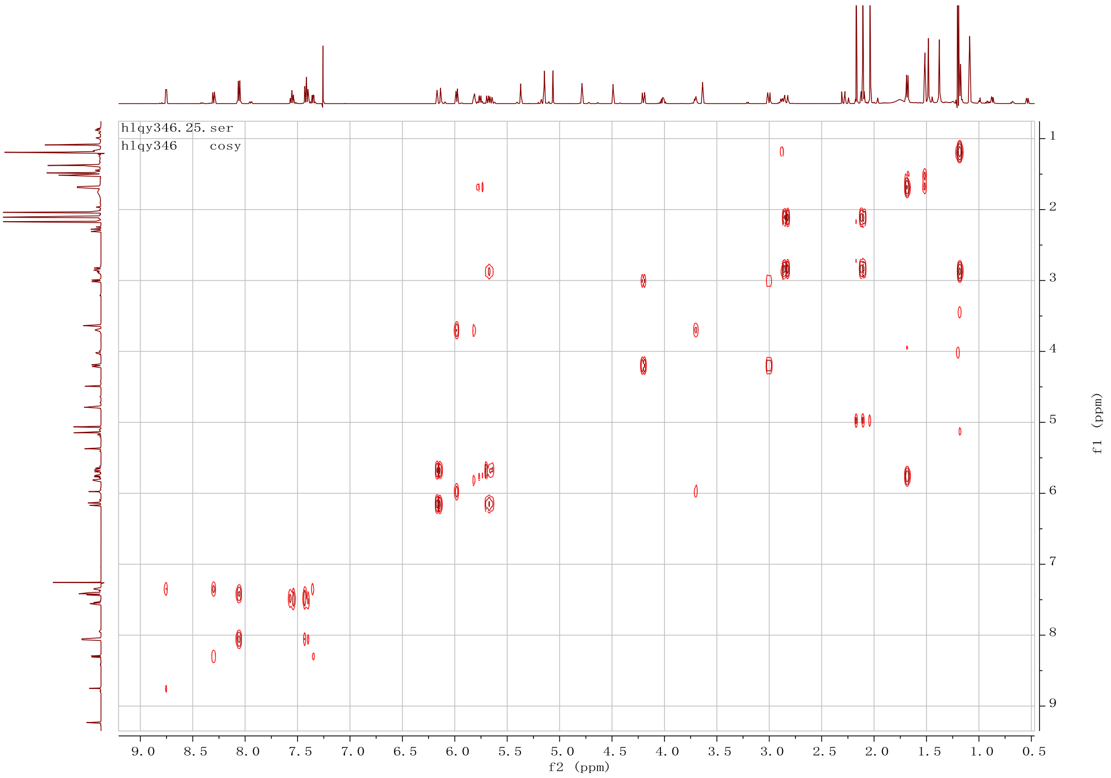

Figure S83. ^{1}H-^{1}H COSY spectrum of euphpepluone K (**9**)

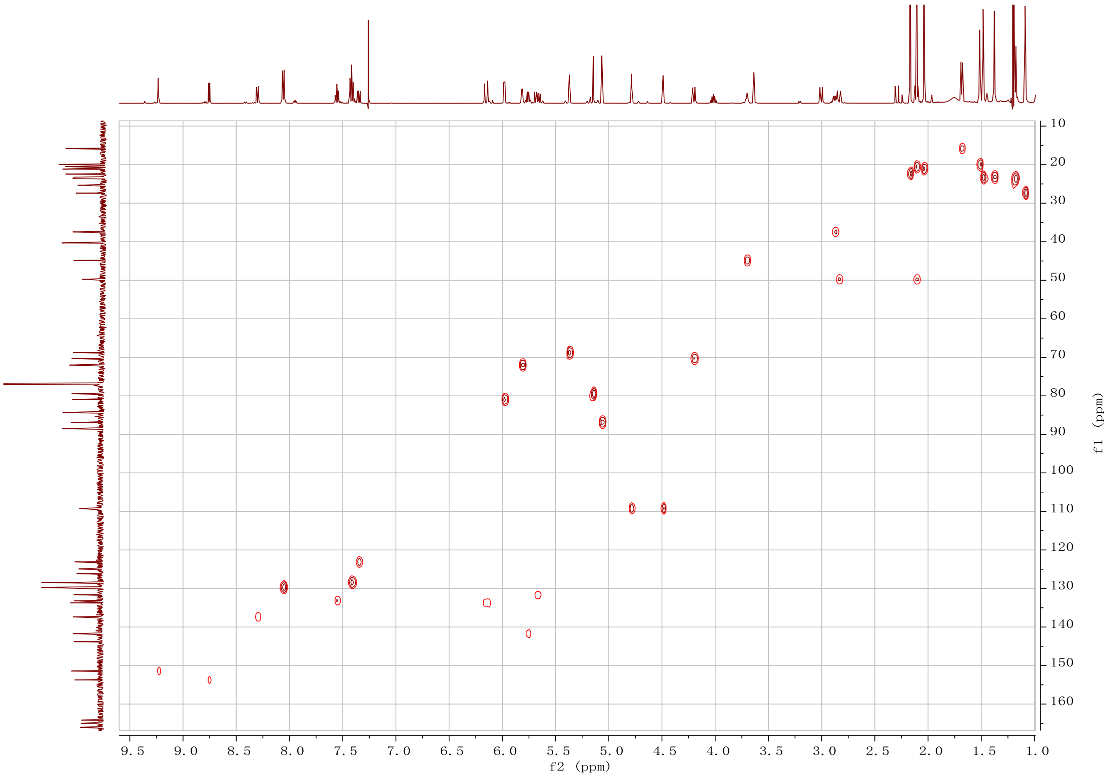

Figure S84. HSQC spectrum of euphpepluone K (**9**)

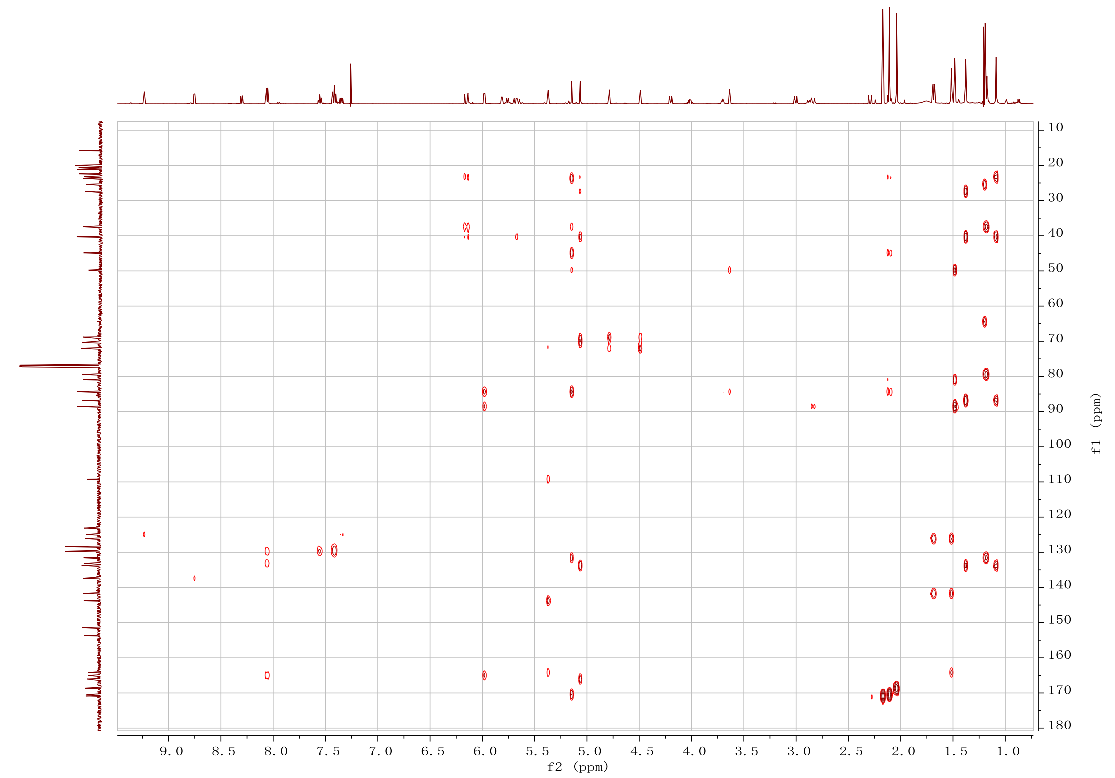

Figure S85. HMBC spectrum of euphpepluone K (**9**)

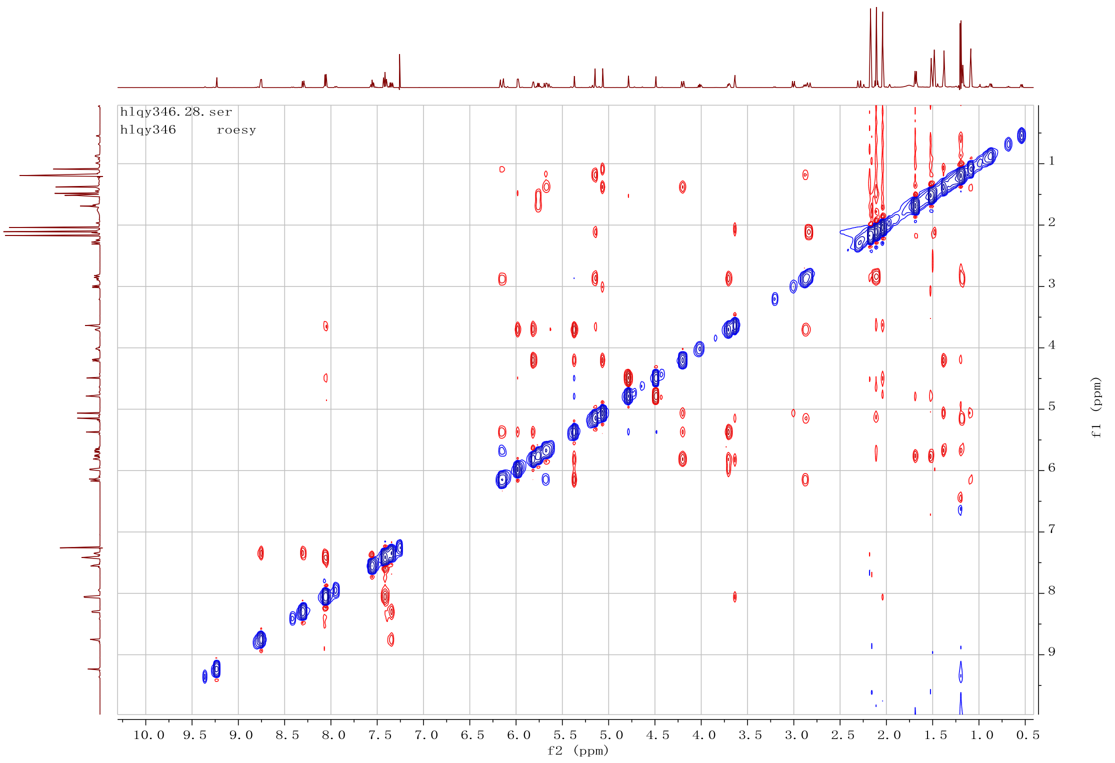

Figure S86. NOESY spectrum of euphpepluone K (**9**)

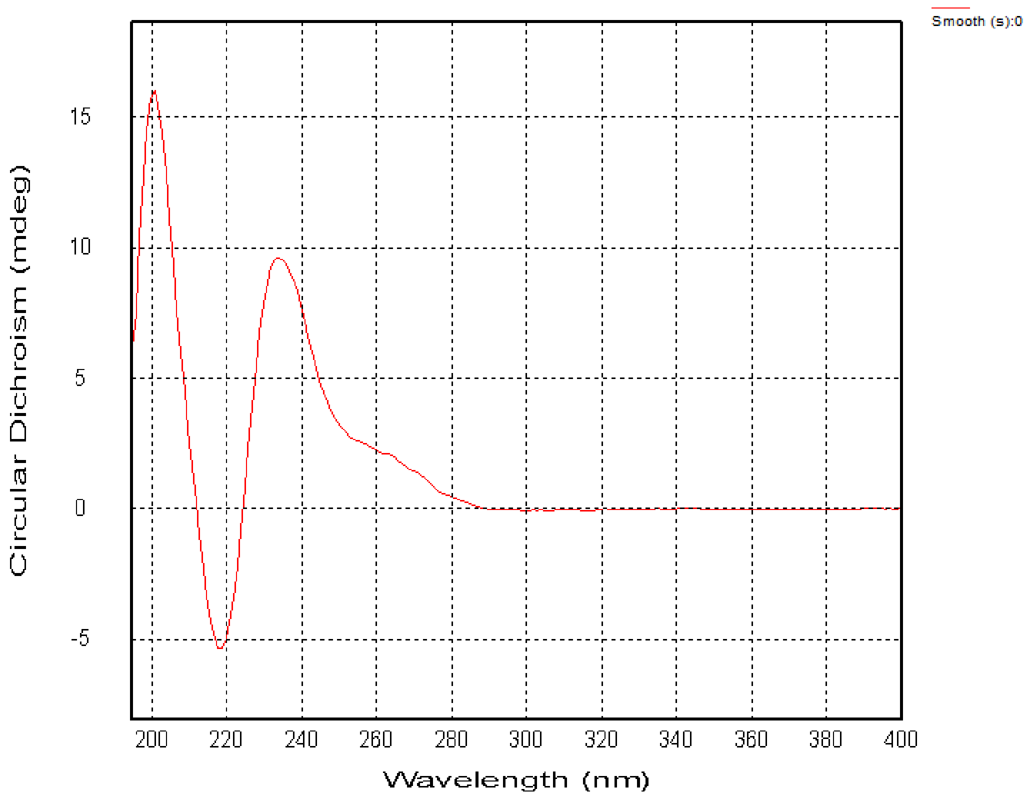

Figure S87. CD spectrum of euphpepluone K (**9**)

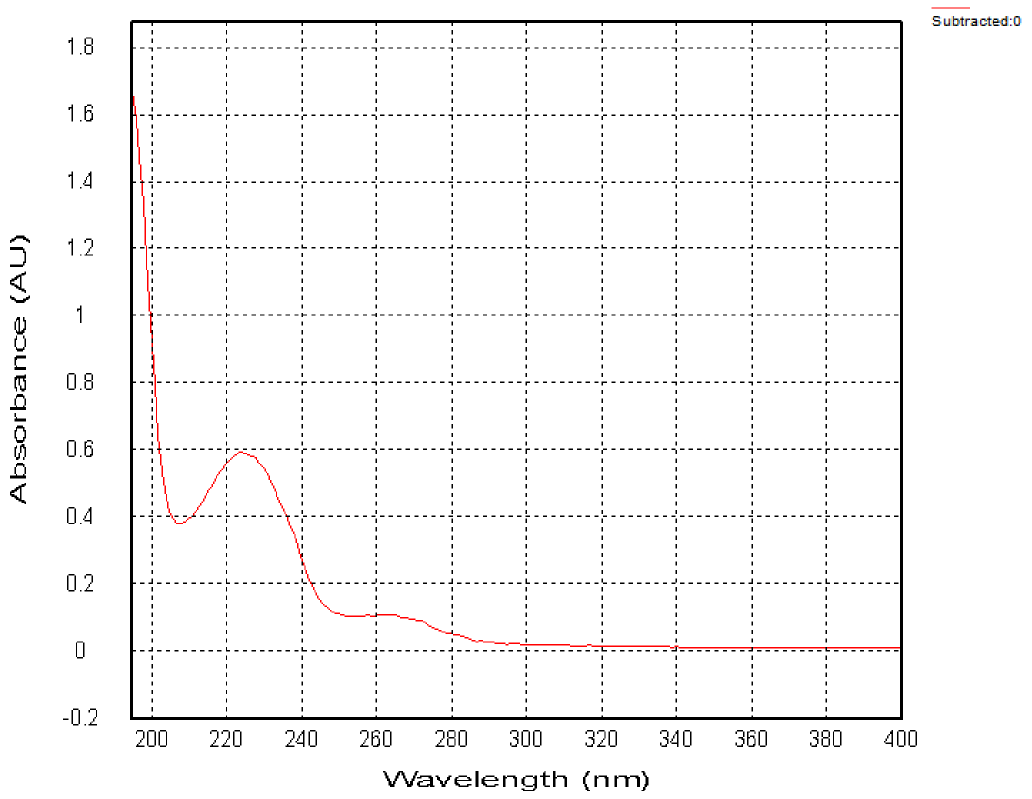

Figure S88. UV spectrum of euphpepluone K (**9**)

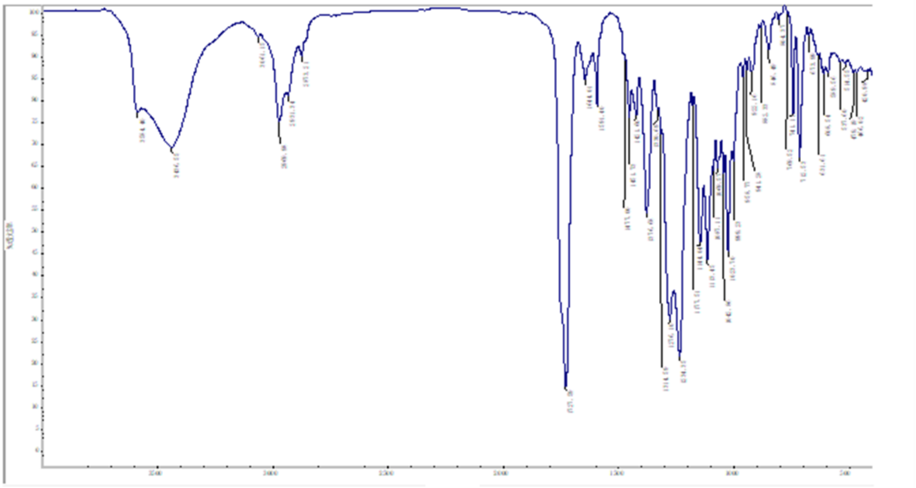

Wavenumber (cm^{-1})

Figure S89. IR spectrum of euphpepluone K (**9**)

Qualitative Analysis Report

**Data Filename**	HLQY-346.d	**Sample Name**	HLQY-346
**Sample Type**	Sample	**Position**	P1-A5
**Instrument Name**	Instrument 1	**User Name**	
**Acq Method**	s.m	**Acquired Time**	8/31/2020 4:02:24 PM
**IRM Calibration Status**	Success	**DA Method**	Default.m
**Comment**			

**Sample Group**		**Info.**
**Acquisition SW Version**	6200 series TOF/6500 series Q-TOF B.05.01 (B5125.2)	

User Spectra

Fragmentor Voltage	Collision Energy	Ionization Mode
135	0	ESI

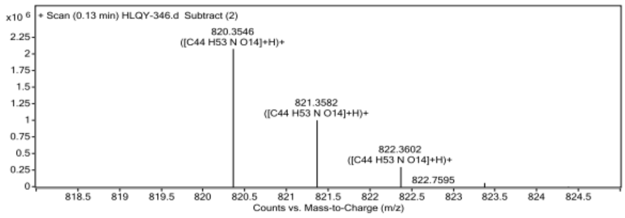

Peak List

m/z	z	Abund	Formula	Ion
274.2744	1	191942.41		
318.3002	1	219795.33		
808.3534	1	261503.28		
809.3565	1	118316.13		
820.3546	1	2078186.63	C44 H53 N O14	(M+H)+
821.3582	1	1010542.56	C44 H53 N O14	(M+H)+
822.3602	1	308538.47	C44 H53 N O14	(M+H)+
836.3476	1	104944.6		
842.3349	1	242545.97		
843.3379	1	113025.63		

Formula Calculator Element Limits

Element	Min	Max
C	3	60
H	0	120
O	0	30
N	0	5

Formula Calculator Results

Formula	CalculatedMass	CalculatedMz	Mz	Diff. (mDa)	Diff. (ppm)	DBE
C44 H53 N O14	819.3466	820.3539	820.3546	-0.70	-0.85	19.0000

--- End Of Report ---

Figure S90. (+)-HRESIMS spectrum of euphpepluone K (**9**)

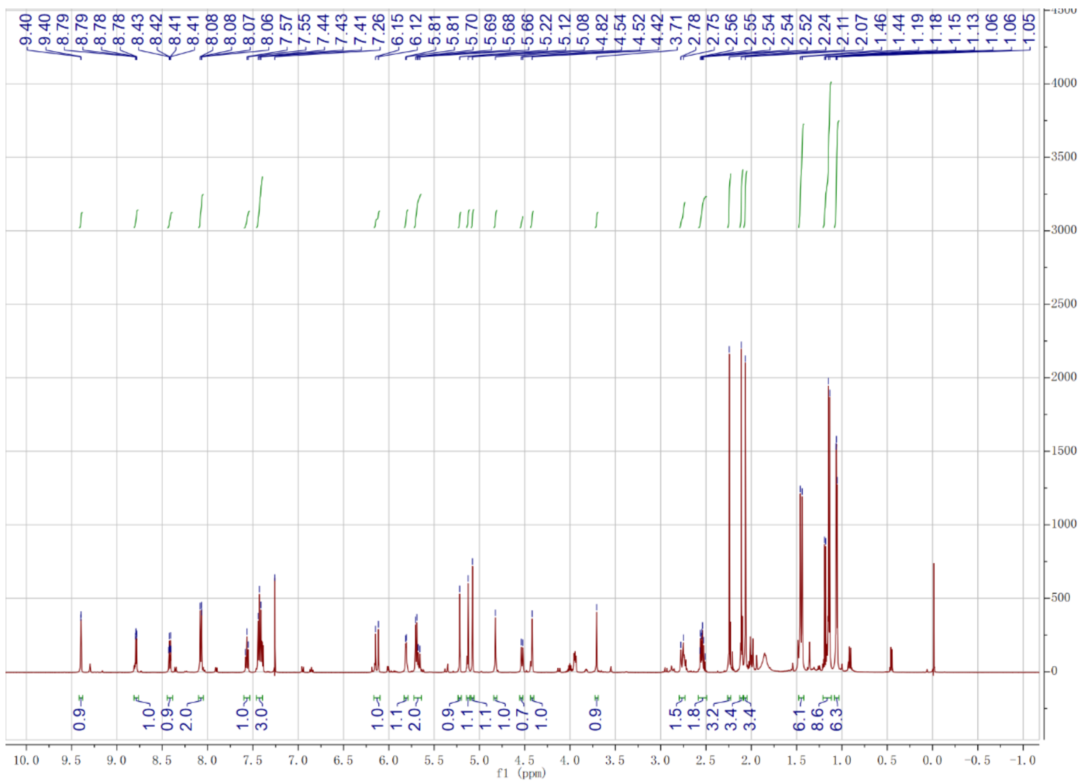

Figure S91. ^{1}H NMR spectrum of euphpepluone L (**10**)

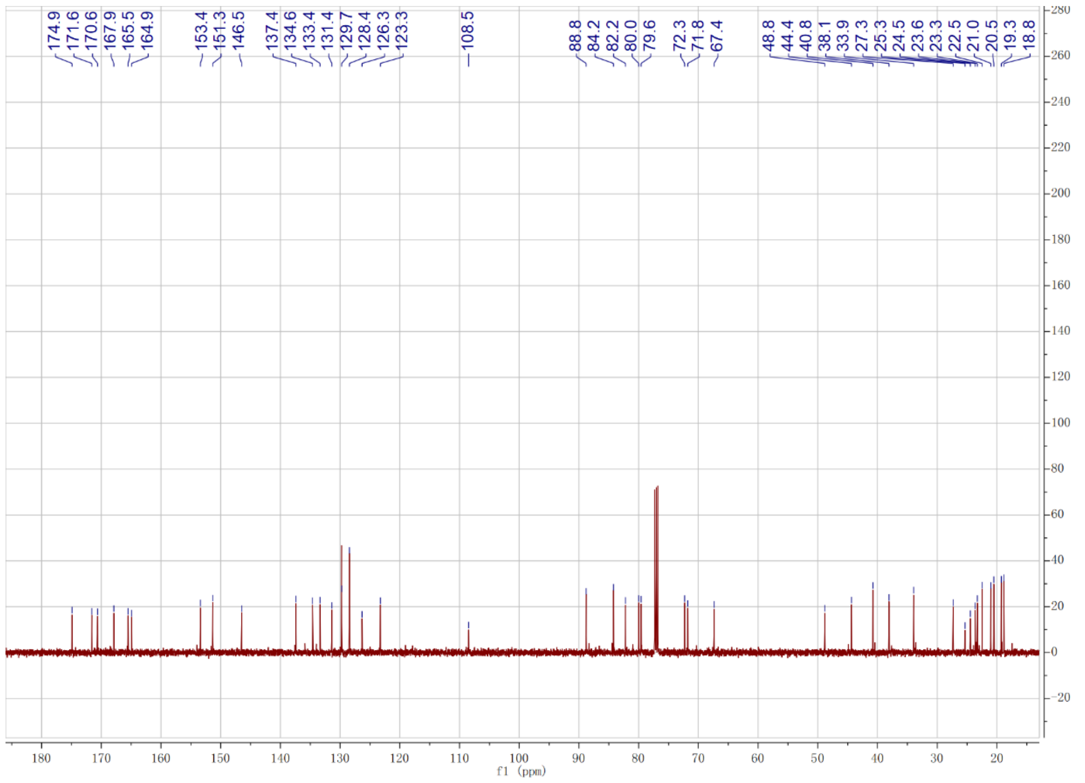

Figure S92. ^{13}C NMR spectrum of euphpepluone L (**10**)

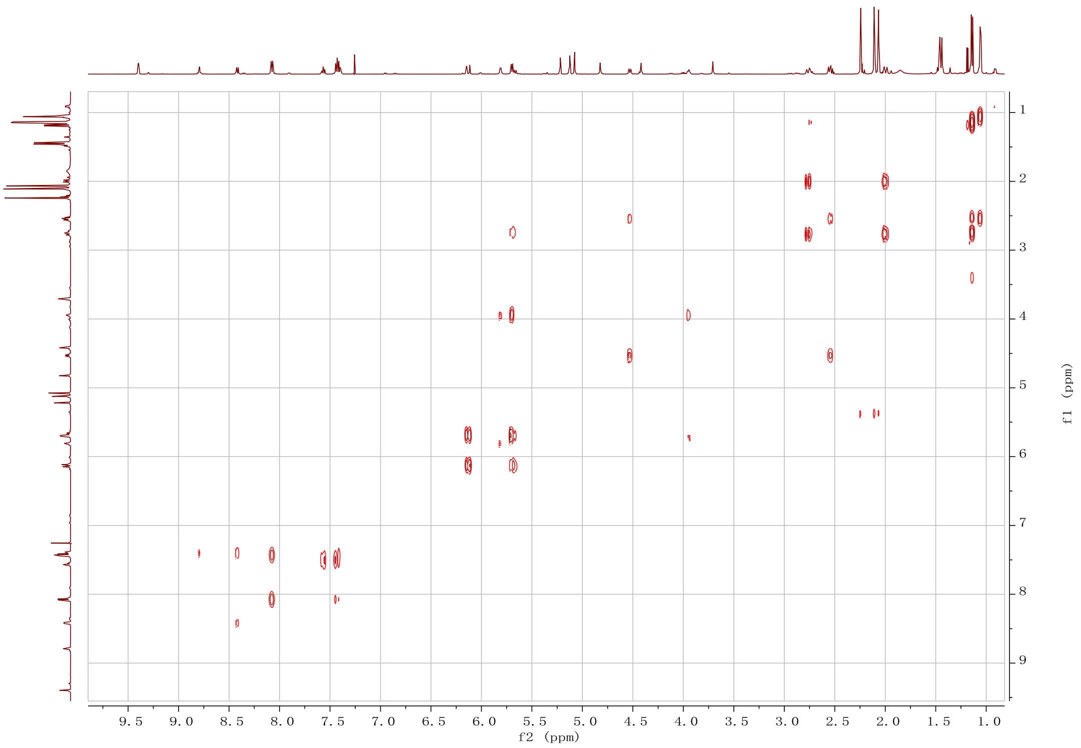

Figure S93. ^{1}H-^{1}H COSY spectrum of euphpepluone L (**10**)

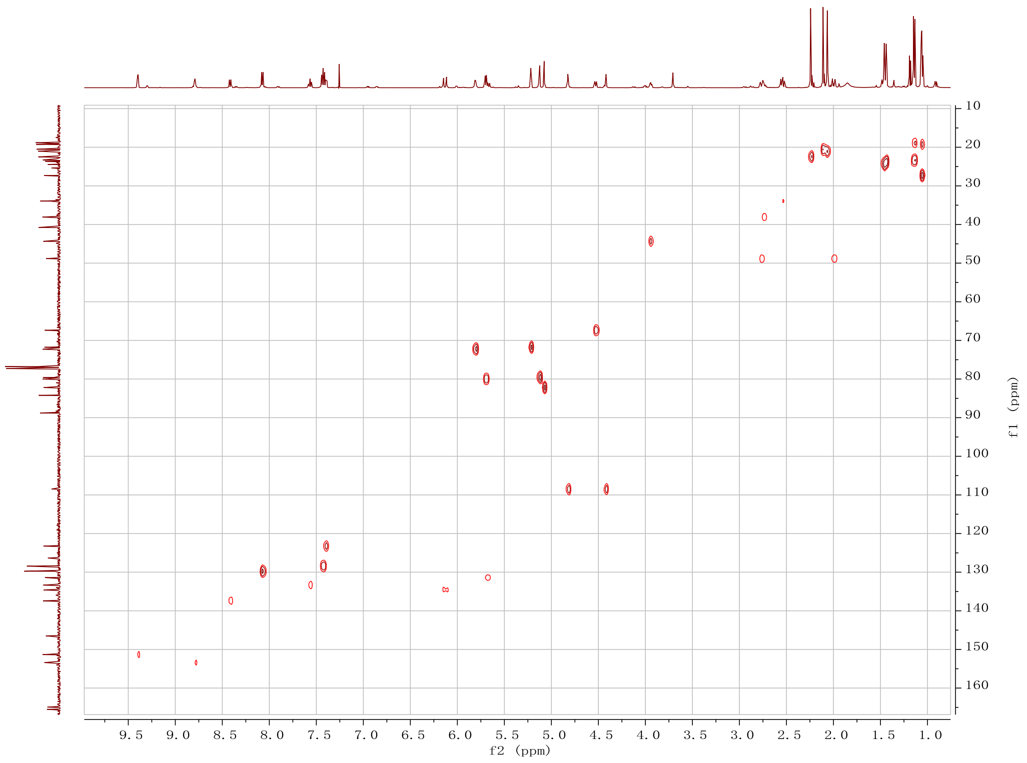

Figure S94. HSQC spectrum of euphpepluone L (**10**)

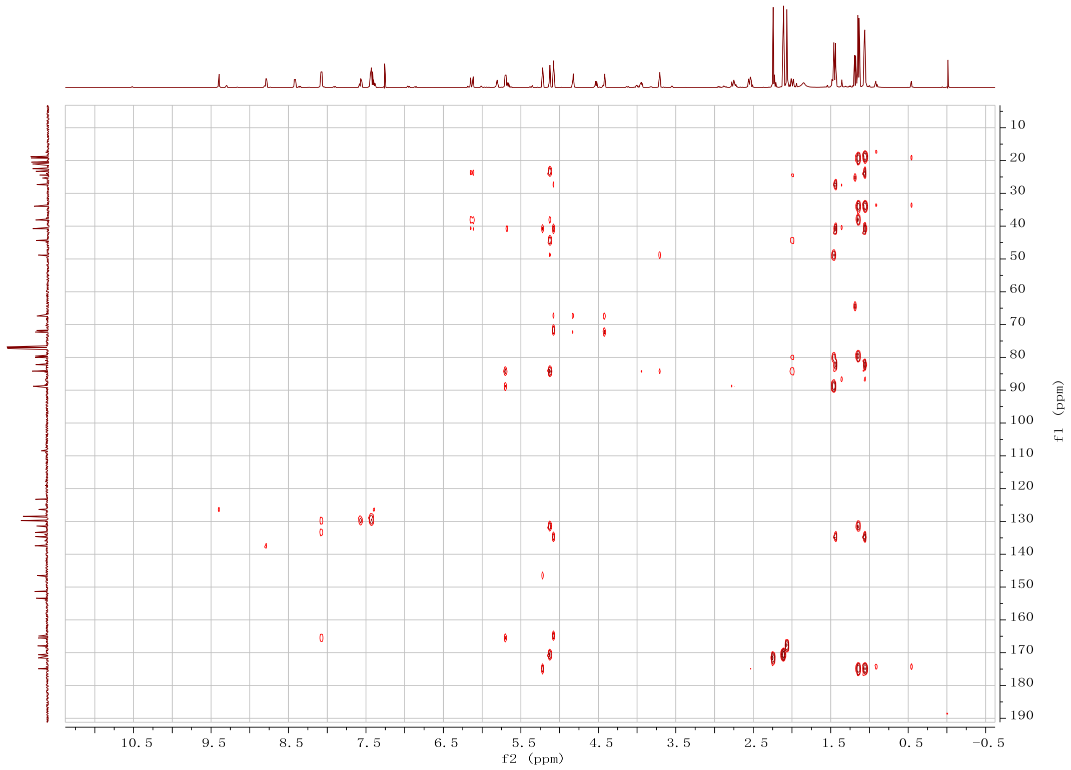

Figure S95. HMBC spectrum of euphpepluone L (**10**)

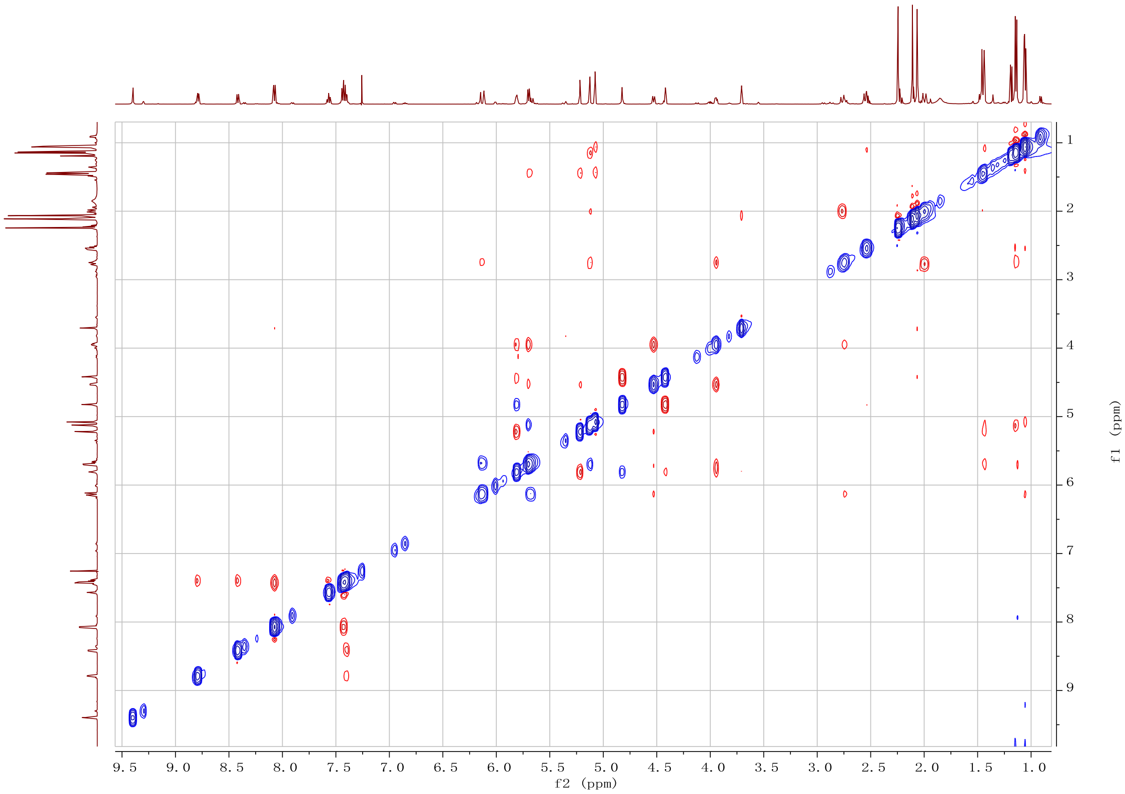

Figure S96. ROESY spectrum of euphpepluone L (**10**)

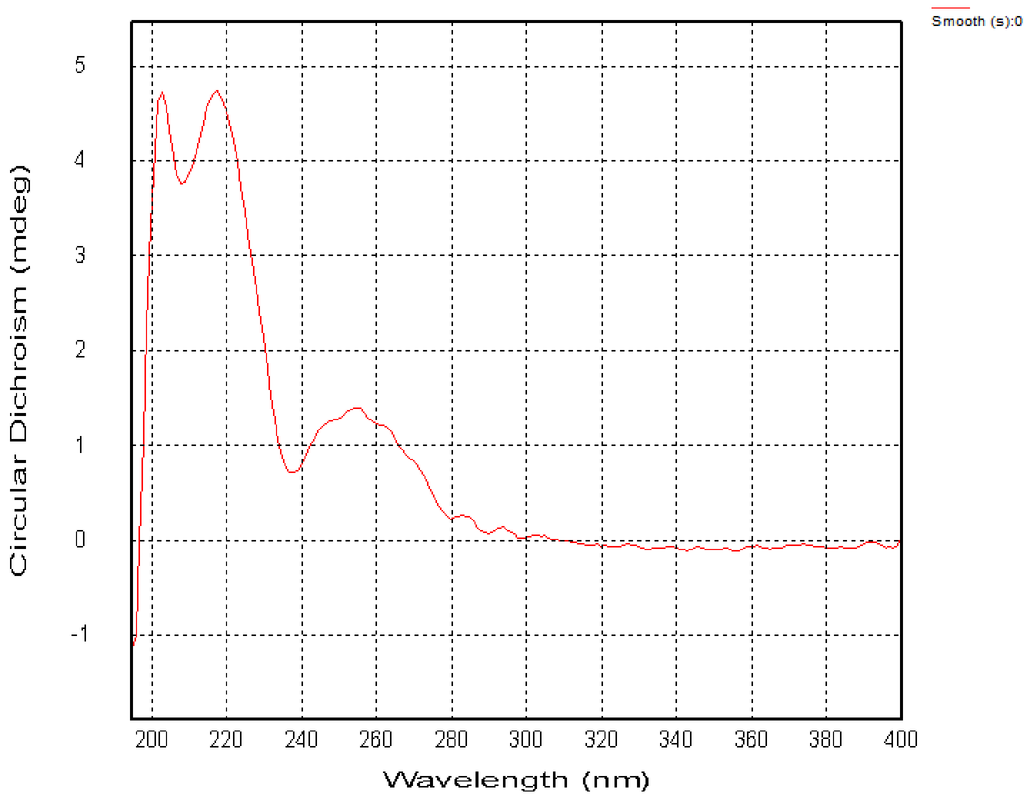

Figure S97. CD spectrum of euphpepluone L (**10**)

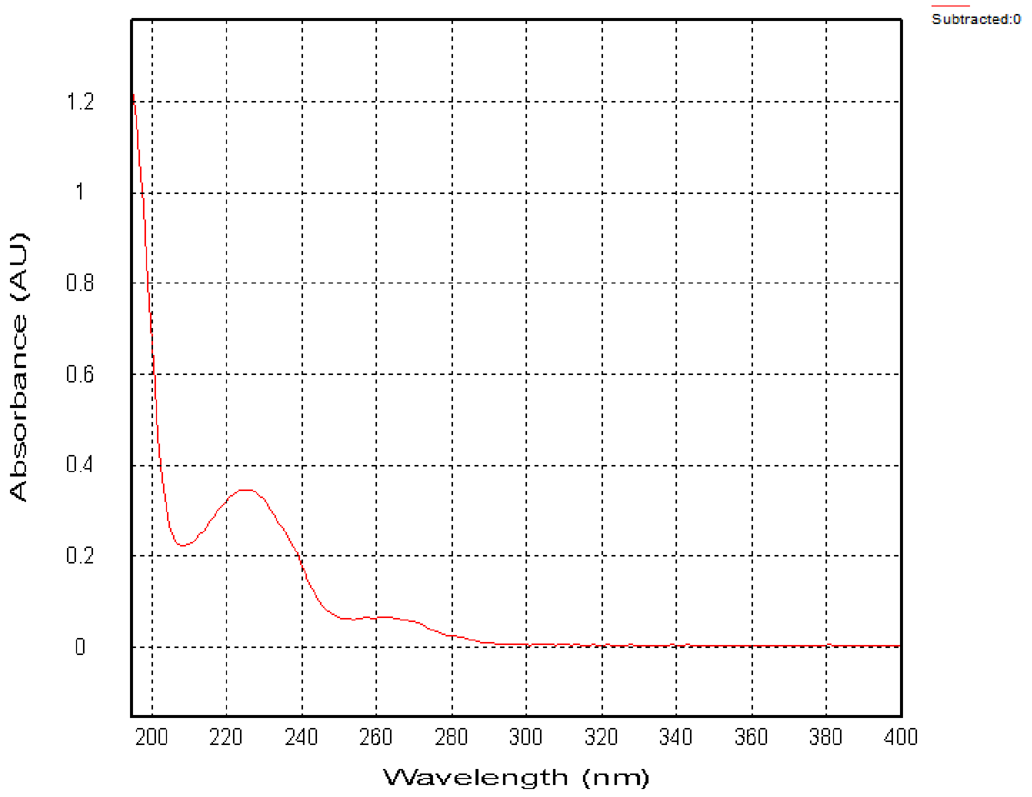

Figure S98. UV spectrum of euphpepluone L (**10**)

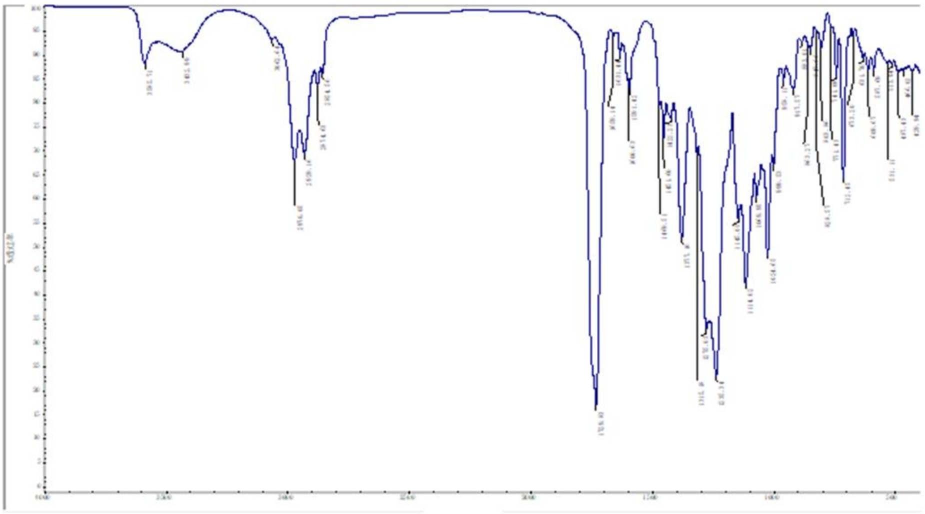

Wavenumber (cm^{-1})

Figure S99. IR spectrum of euphpepluone L (**10**)

Qualitative Analysis Report

**Data Filename**	HLQY-135.d	**Sample Name**	HLQY-135
**Sample Type**	Sample	**Position**	P1-A7
**Instrument Name**	Instrument 1	**User Name**	
**Acq Method**	s.m	**Acquired Time**	8/31/2020 4:05:00 PM
**IRM Calibration Status**	Success	**DA Method**	Default.m
**Comment**			

**Sample Group**		**Info.**
**Acquisition SW**	6200 series TOF/6500 series	
**Version**	Q-TOF B.05.01 (B5125.2)	

User Spectra

Fragmentor Voltage	Collision Energy	Ionization Mode
135	0	ESI

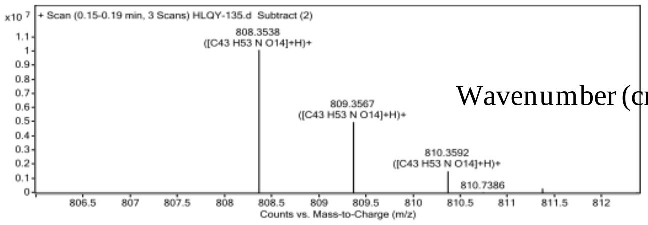

Wavenumber (cm^{-1})

Peak List

m/z	z	Abund	Formula	Ion
138.0546	1	250957.7		
274.2737	1	210727.61		
318.2999	1	236259.41		
764.327	1	226453.28		
808.3538	1	10106264	C43 H53 N O14	(M+H)+
809.3567	1	5025067.5	C43 H53 N O14	(M+H)+
810.3592	1	1573988.63	C43 H53 N O14	(M+H)+
811.3621	1	325669.66	C43 H53 N O14	(M+H)+
830.3345	1	541736.94		
831.3375	1	253886.89		

Formula Calculator Element Limits

Element	Min	Max
C	3	60
H	0	120
O	0	30
N	0	5

Formula Calculator Results

Formula	CalculatedMass	CalculatedMz	Mz	Diff. (mDa)	Diff. (ppm)	DBE
C43 H53 N O14	807.3466	808.3539	808.3538	0.10	0.12	18.0000

--- End Of Report ---

Figure S100. (+)-HRESIMS spectrum of euphpepluone L (**10**)

Table S1. ^{1}H and ^{13}C NMR Data of Euphpepluones G, K, and L in $CDCl_3$[a]

	Euphpepluone G		Euphpepluone K		Euphpepluone L	
position	δ_H (J in Hz)	δ_C	δ_H (J in Hz)	δ_C	δ_H (J in Hz)	δ_C
1a	2.66 d (14.2)	50.3	2.84 d (14.5)	49.9	2.76 d (13.9)	49.0
1b	2.13 dd (14.0, 9.9)		2.11 d (14.5)		2.00 d (13.9)	
2		88.5		88.7		88.9
3	5.86 d (5.7)	80.5	5.98 d (5.9)	81.1	5.71 d (6.3)	80.1
4	3.36 t (4.4)	44.7	3.71dd (5.5, 4.1)	45	3.94 dd (6.1, 4.3)	44.5
5	5.74 d (2.6)	71.8	5.82 d (3.5)	72.1	5.81 d (3.5)	72.4
6		144.2		143.9		146.6
7a	5.23 s	68.5	5.38 s	68.9	4.54 s	67.5
7b						
8a	4.03 d (11.0)	70.1	4.21 s	70.4	5.22 s	71.9
8b						
9	4.72 s	86.1	5.07 s	87.1	5.09 s	82.4
10		40.1		40.4		40.9
11	5.91 d (16.0)	134	6.15 d (15.9)	133.8	6.13 d (15.8)	134.7
12	5.57 dd (16.0, 9.5)	131.1	5.67 dd (16.0, 9.7)	131.7	5.69 dd (15.7, 9.7)	131.5
13	2.67 m	37.6	2.88 m	37.6	2.74 m	38.2
14	5.09 s	79.4	5.15 s	79.6	5.13 s	79.7
15		84.1		84.5		84.3
16	1.51 s	22.7	1.48 s	23.5	1.46 s	24.6
17a	4.87 s	109.4	4.79 s	109.4	4.83 s	108.6
17b	4.49 s		4.49 s		4.42 s	
18	1.00 s	27.3	1.09 s	27.5	1.07 s	27.4
19	1.28 s	23.3	1.38 s	23.4	1.44 s	23.8
20	1.13 d (7.0)	23.7	1.18 d (7.1)	23.8	1.14 d (6.9)	23.4
OAc-2						
C=O		170.7		171.0		171.7
	2.14 s	22.4	2.17 s	22.5	2.25 s	22.6
OAc-3						
C=O						
OAc-5						
C=O		168.6		168.8		168.0
	1.99 s	21.2	2.04 s	21.2	2.07 s	21.1
OAc-7						
C=O						
OAc-8						
C=O						
OAc-9						
C=O		171.9				
	2.04 s	20.6				
OAc-14						
C=O		170.5,		170.6		170.7
	2.07 s	20.7	2.11 s	20.6	2.11	20.6

OAc-15						
C=O						
OBz-3						
C=O		165.0		165.1		165.6
1'		130.1		130.1		129.7
2',6'	8.07 d (8.2)	129.8	8.06 d (8.4)	129.8	8.08 d (8.0)	129.9
3',5'	7.43 t (7.3)	128.5	7.42 t (8.0)	128.5	7.43 t (7.8)	128.6
4'	7.56 t (7.2)	133.3	7.56 t (7.4)	133.3	7.57 t (7.4)	133.5
OPrp-7						
C=O		172.0				
1''	2.36 m	27.6				
2''	1.17d (14.0)	9.1				
OTig-7						
C=O				164.3		
1''				126.2		
2''			5.77 m	141.9		
3''			1.69 dd (7.3, 1.4)	16.0		
4''			1.52 s	20.1		
OiBu-8						
C=O						175.0
1''					2.54 m	34.1
2''					1.14 d (7.0)	18.9
3''					1.06 d (6.9)	19.4
ONic-9						
C=O				165.9		164.9
2'''			9.25 d (1.4)	151.1	9.41 br s	151.1
3'''				125.3		126.6
4'''			8.34 dt (8.0, 1.7)	138.0	8.44 dt (7.9, 1.8)	137.9
5'''			7.39 dd (7.9, 4.9)	123.4	7.43 m	123.5
6'''			8.77 dd (4.7, 1.3)	153.4	8.79 d (4.8)	153.2

a: Yang, Y.; Zhou, M.; Wang, D.; Liu, X.; Ye, X.; Wang, G.; Lin, T.; Sun, C.; Ding, R.; Tian, W.; Chen, H. F., Jatrophane Diterpenoids from Euphorbia peplus as Multidrug Resistance Modulators with Inhibitory Effects on the ATR-Chk-1 Pathway. *J Nat Prod* **2021,** 84 (2), 339-351.
